# Organophotocatalytic Functionalization of 3,4-Dihydroquinoxalin-2-ones with Isoxazol-5-amines Using Visible Light

**DOI:** 10.1021/acs.joc.5c02592

**Published:** 2025-12-09

**Authors:** Salma E. Mora-Rodríguez, Jaume Rostoll-Berenguer, Selene Lagunas, Miguel A. Vázquez, Gonzalo Blay, José R. Pedro, Carlos Vila

**Affiliations:** † Departament de Química Orgànica, Facultat de Química, 16781 Universitat de València, Dr. Moliner 50, 46100 Burjassot, Spain; ‡ Departamento de Química, 14654 Universidad de Guanajuato, Noria Alta s/n, 36050 Guanajuato, Mexico; § ImX-CONACyT, Departamento de Química, Universidad de Guanajuato, Noria Alta s/n, 36050 Guanajuato, Mexico

## Abstract

An organophotocatalytic strategy for the functionalization of 3,4-dihydroquinoxalin-2-ones with isoxazol-5-amines is reported. Using commercially available organophotocatalysts under visible-light irradiation and mild reaction conditions, a series of 20 seven 3,4-dihydroquinoxalin-2­(1*H*)-one derivatives bearing a 5-aminoisoxazol-4-yl moiety at C-3 have been synthesized. The alkylation of 5-aminoisoxazole proceeds in a chemoselective manner, affording substitution exclusively at the C-4 position of this heterocycle. This process was also scaled up to 1 mmol, revealing the formation of an isoxazol-2-one derivative as a byproduct. The mechanism of this transformation has also been investigated through steady-state luminescence quenching experiments among other techniques.

## Graphical abstract

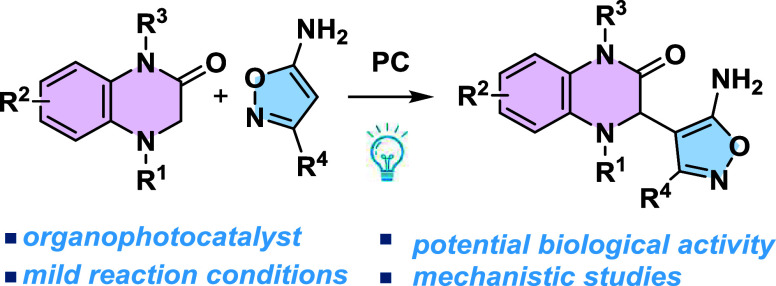

## Introduction

In recent years, synthetic photochemistry with visible light has emerged as a powerful and sustainable approach for developing new transformations in organic synthesis.^1–6^ The use of visible light as an energy source enables the activation of photocatalysts under mild conditions, thereby avoiding the harsh reaction conditions often associated with ultraviolet irradiation.^7^ This strategy offers significant advantages including broader functional group tolerance, reduced substrate degradation, and a lower environmental impact. A variety of photocatalysts, ranging from transition-metal complexes (such as those based on ruthenium and iridium) to purely organic dyes (including acridines, flavins, and eosin derivatives), can absorb photons in the visible spectrum to trigger single-electron transfer (SET) or energy transfer processes.^2,8^ These photochemical events allow the generation of radical intermediates or excited states with high reactivity under mild reaction conditions, thus unlocking synthetic pathways that are often inaccessible through purely thermal methods. The advances in the design of new photocatalysts and the mechanistic understanding of these photoinduced processes have greatly expanded the scope of visible-light photochemistry.^9^

Amines, particularly tertiary amines, have been widely used in the context of visible-light photoredox catalysis due to their ability to undergo single-electron oxidation in the presence of a suitable photocatalyst.^10,11^ Depending on the reaction conditions, the reactive intermediate may be either an α-amino radical exhibiting nucleophilic character or an iminium cation, which acts as an electrophile.^12^ This divergent yet complementary character of organic amines has led to the development of a huge library of protocols for their functionalization, either nucleophilically^13–16^ or electrophilically.^17,18^ Especially, *N*-substituted tetrahydroisoquinolines, *N,N*-dialkyl anilines, and glycine derivatives have been extensively resorted in this context.^12,19^

In contrast, 3,4-dihydroquinoxalin-2-ones are a particular class of amines that bear significant relevance in medicinal chemistry and agrochemistry (Figure 1A).^20–22^ However, the development of synthetic methodologies for the functionalization of these amines using visible-light photocatalysis has gained importance since the pioneering report of Hong in 2018 ^23^ and that of our research group in 2019.^24^

**Figure 1:**
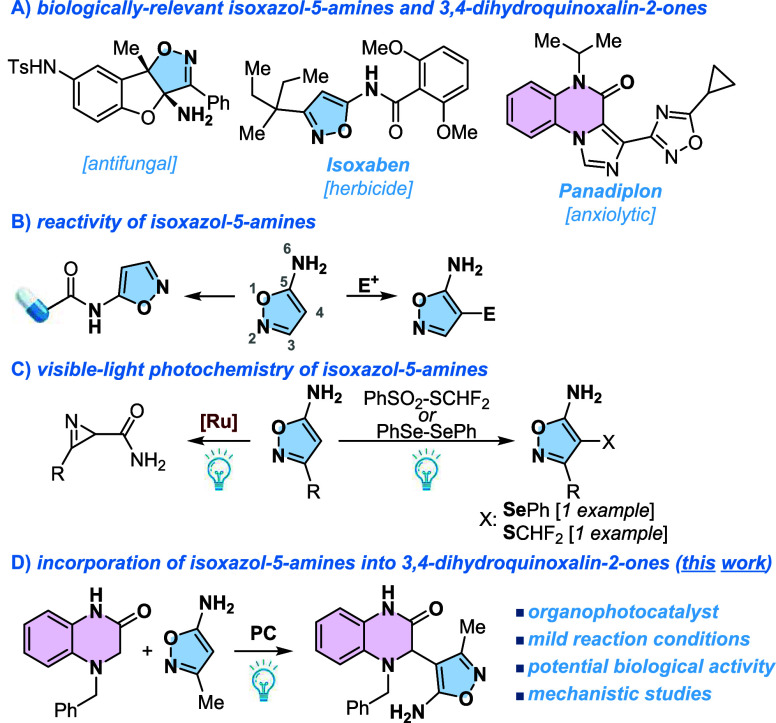
(A) Biologically relevant isoxazol-5-amines and 3,4-dihydroquinoxalin-2-ones. (B) Reactivity of isoxazol-5-amines. (C) Visible-light photochemistry of isoxazol-5-amines. (D) Incorporation of isoxazol-5-amines into 3,4-dihydroquinoxalin-2-ones.

Since then, the photochemical functionalization of these nitrogen heterocycles has gained huge interest.^25–33^ Among them, we reported the use of pyrazol-3-ones and 5-aminopyrazoles as nucleophiles in the photocatalytic C-3 functionalization of 3,4-dihydroquinoxalin-2-ones.^34^ Due to that, we were interested in expanding the application of this photochemical methodology to other nucleophilic five-member heterocycles.

In this context, we focused our attention on isoxazol-5-amines, valuable building blocks that have been employed to incorporate the isoxazole moiety into drug candidates.^35–38^ Isoxazoles are typically electron-deficient aromatic heterocycles, which make electrophilic substitution reactions challenging. One strategy to enhance the reactivity of such aromatic systems is the introduction of exocyclic electron-donating groups such as hydroxyl, alkoxy, or amino substituents. These groups increase the electron density on the aromatic ring, thereby facilitating reactions such as Friedel–Crafts alkylation or other electrophilic substitutions.^39^ However, this feature poses intrinsic chemoselectivity challenges (Figure 1B), since the exocyclic nitrogen, as well as the carbon at the 4-position, exhibits potential nucleophilicity. In fact, the amino group in isoxazol-5-amines usually serves as a synthetic handle for the incorporation of the isoxazole moiety.^35^ In contrast, the nucleophilicity of the C-4 position arises from the enamine-like structure. Numerous compounds bearing this heterocyclic motif have shown diverse biological activities, including antifungal and herbicidal properties (Figure 1A).^40–43^ However, photochemical functionalizations of this heterocycle are scarce in the literature. In 2018, Loh and co-workers reported a rearrangement of isoxazol-5-amines into 2*H*-azirinyl carboxamides using the Hoveyda–Grubbs II catalyst under blue light irradiation (Figure 1C).^44^ Regarding bimolecular methodologies, only two examples have been reported to date. In 2018, Li and co-workers achieved a C-4 difluoromethylthiolation of an isoxazol-5-amine under white light irradiation.^45^ More recently, in 2022, Choudhury and co-workers described the selenylation of various aromatic substrates including an isoxazol-5-amine.^46^

## Results and Discussion

Given the limited number of photochemical methods available for the functionalization of isoxazol-5-amines, we initiated a program aimed at functionalizing these bidentate nucleophilic heterocycles with 3,4-dihydroquinoxalin-2-ones (Figure 1D). We hypothesized that under visible-light photoredox catalysis in an aerobic atmosphere, it would be possible to generate the corresponding iminium cation from 3,4-dihydroquinoxalin-2-ones. These catalytically generated electrophiles could then undergo a polar reaction with isoxazol-5-amines. We selected 3,4-dihydroquinoxalin-2-one **1a** and isoxazol-5-amine **2a** as model substrates for the benchmark reaction.

We started the optimization campaign by reacting equimolar amounts of **1a** and **2a** without any photocatalyst in CHCl_3_ as a solvent (Table 1, entry 1). After 29 h, the C-4 functionalized isoxazol-5-amine **3aa** was isolated in 28% yields.^23,34^ To confirm the need of visible-light irradiation, we performed the same reaction in the dark, obtaining in this case no consumption of any substrate (Table 1, entry 2). Seeking to improve the yield, we screened a series of photocatalysts (Figure 2). When either Ru­(bpy)_3_Cl_2_ or *fac*-Ir­(ppy)_3_ was tested, a diminished yield was obtained, probably due to the ability of these metal-based photocatalysts to trigger nonproductive pathways (Table 1, entries 3 and 4). Actually, examination of the crude mixture by ^1^H NMR revealed the generation of an overoxidation product of **3aa**, among other unidentified compounds. This finding prompted us to carefully control the irradiation time of the reaction until 3,4-dihydroquinoxalin-2-one **1a** was completely consumed as showed by TLC. Organophotocatalysts such as eosin Y–Na_2_ (**PC1**), 4CzIPN (2,4,5,6-tetrakis­(9*H*-carbazol-9-yl) isophthalonitrile, **PC2**), or 9-mesityl-10-methylacridinium-BF_4_ (**PC3**) did not show any improvement as well (Table 1, entries 5–7). To our delight, when 2,4,6-triphenylpyrylium-BF_4_ (**PC4**) was employed, isoxazol-5-amine **3aa** was isolated in a 49% yield (Table 1, entry 8). Similarly, 9,10-phenanthrenequinone (**PC5**), which has been used by our research group previously, generated the desired product in 53% yields (Table 1, entry 9). To further improve the yield, we increased the amount of 3,4-dihydroquinoxalin-2-one **1a** to 1.3 equiv while testing **PC4** and **PC5**. Using **PC4** under these conditions, the yield increased to 54% (Table 1, entry 10), but the reaction proceeded more slowly and required 23 h for completion. However, employing **PC5** led to a lower yield (Table 1, entry 11) with a similar reaction time (6 h). Based on these results, we decided to maintain this slight excess of **1a** over **2a**. A brief solvent screening showed that the reaction outcome was largely insensitive to the choice of the solvent, although CHCl_3_ gave superior results (Table 1, entries 12–14). Regarding the reaction times, when DMF was used as a solvent, the reaction proceeded faster but afforded a slightly lower yield.

**Figure 2:**
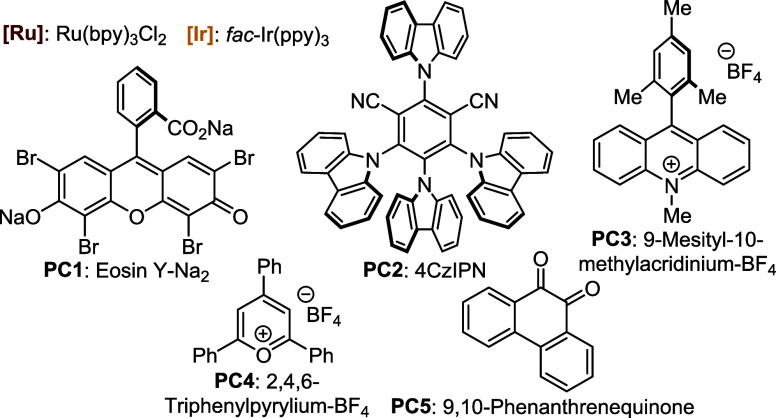
Tested photocatalysts.

**Table 1:**
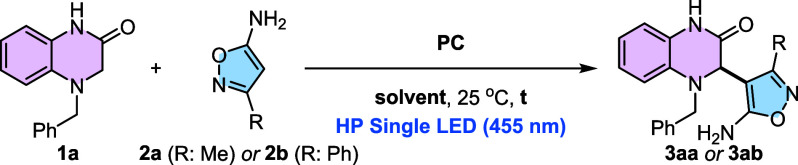
Optimization of the Reaction Conditions^a^

entry	PC (*x* mol %)	solvent	*t* (h)	yield 3 (%)^b^
1		CHCl_3_	29	28
2^c^		CHCl_3_	29	NR
3	**[Ru]** (1)	CHCl_3_	23	26
4	**[Ir]** (1)	CHCl_3_	23	17
5	**PC1** (5)	CHCl_3_	6	25
6	**PC2** (2)	CHCl_3_	4.5	10
7	**PC3** (5)	CHCl_3_	5	12
8	**PC4** (5)	CHCl_3_	8	49
9	**PC5** (5)	CHCl_3_	6	53
10^d^	**PC4** (5)	CHCl_3_	23	54
11^d^	**PC5** (5)	CHCl_3_	6	47
12^d^	**PC4** (5)	MeCN	22	50
13^d^	**PC4** (5)	DMF	8	51
14^d^	**PC4** (5)	DCM	24	47
15^d^ ^,^ ^e^	**PC4** (5)	CHCl_3_	9	55
16^c^ ^,^ ^d^	**PC5** (5)	CHCl_3_	6	55
17^f^	**PC5** (5)	CHCl_3_	24	NR
18^g^	**PC5** (5)	CHCl_3_	24	11

a Reaction conditions: **1a** (0.1 mmol), **2a** (0.1 mmol), PC (x mol %), and solvent (1 mL) under HP single LED (455 nm) irradiation.

b Yield determined after purification by column chromatography.

c Reaction performed in the dark.

d Reaction performed with 1.3 equiv of **1a.**

e Reaction performed with **2b.**

f Reaction performed with 0.13 mmol of TEMPO.

g Reaction performed under an argon atmosphere.

Finally, we were interested in testing isoxazol-5-amine bearing an aromatic ring at C-3. To this end, isoxazol-5-amine **2b** was tested under the same reaction conditions using either **PC4** or **PC5** (Table 1, entries 15–16). Gratifyingly, compound **3ab** was isolated in 55% yields in both cases.

Finally, two control experiments were conducted. First, the reaction between **1a** and **2a** was performed in the presence of 1.3 equiv of TEMPO, a known radical scavenger (Table 1, entry 17). Under these conditions, product **3aa** was not detected by means of ^1^H NMR analysis, confirming the radical nature of the photochemical process. Then, the same transformation was launched under an argon atmosphere, resulting in the formation of the expected product **3aa** in only 11% yields after 24 h of irradiation.

After developing a set of reaction parameters that are valid for isoxazol-5-amines that bear aliphatic or aromatic substituents at C-3 we were interested in exploring the generality of this transformation. First, we evaluated the effect of different substitution patterns in isoxazol-5-amine **2** (Scheme 1).

**Scheme 1:**
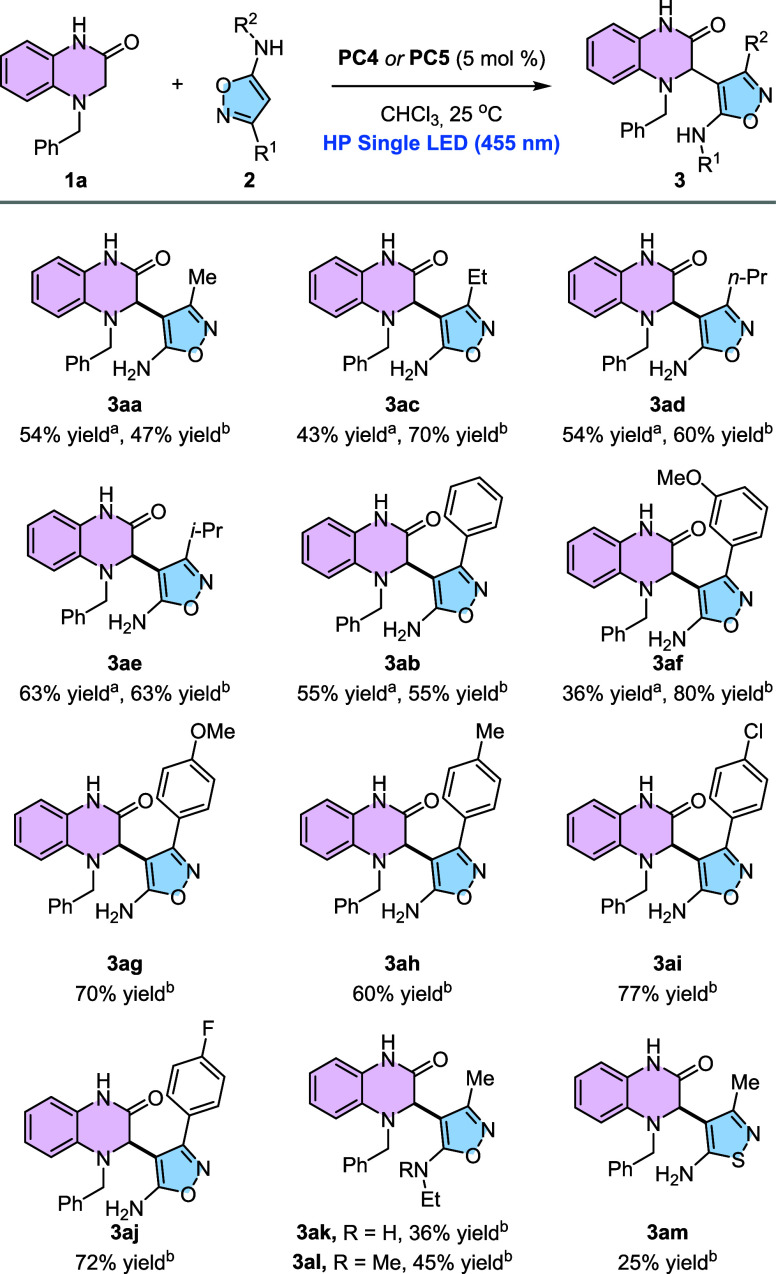
Scope of the Reaction Using Differently Substituted Isoxazol-5-Amines^a^

a Reaction conditions: **1a** (0.13 mmol), **2** (0.1 mmol), **PC** (5 mol %), CHCl_3_ (1 mL) under the irradiation of HP single LED (455 nm). The yield was determined after purification by column chromatography.
b
**PC4** was used.
c
**PC5** was used.

We decided to test both **PC4** and **PC5** as organophotocatalysts. The use of isoxazol-5-amine **2** with longer and ramificated saturated carbon chains at C-3 (ethyl, *n*-propyl, and *i*-propyl) was examined. Isoxazol-5-amine **2c**, which bears an ethyl group, delivered the expected product in 43% using **PC4**, whereas the yield was increased to 70% when **PC5** was present. This sharp difference was not observed when substrate **2d** or **2e** was used. Then, we moved our attention to isoxazol-5-amines bearing aromatic substituents at C-3. Benchmark substrate **2b** delivered the desired product in 55% yields, regardless of the photocatalyst employed. However, when a 3-methoxybenzene substituent was present (substrate **2f**), the expected product was isolated in 36% yields with **PC4** and in 80% yields with **PC5**. This result led us to test more C-3-arometic isoxazol-5-amines with only with **PC5**. Strong (OMe) or moderate (Me) electron-donating substituents at the *para* position of the aromatic substituent in isoxazol-5-amines did not affect the outcome of the reaction, since the expected products **3ag** and **3ah** were isolated in 70% and 60% yields, respectively. The same behavior was observed in isoxazol-5-amines containing electron-withdrawing halogenated groups. Specifically, products **3ai** and **3aj** bearing either a *p*-Cl or a *p*-F were obtained in 77% and 72% yields, confirming the robustness of the transformation to aromatic substituents with opposite electronic features. This high tolerability observed in isoxazol-5-amines led us to explore the effect that the substitution of the amine nitrogen has in the reactivity. Pleasingly, substrate **2k** bearing a *N*-ethyl group was able to generate the desired product **3ak** in 36% yields. In the same sense, the use of *N,N*-ethylmethyl isoxazol-5-amine **2m** permitted us to obtain the expected product **3al** in 45% yields. Finally, we also attempted to extend our methodology to isothiazol-5-amine derivatives. When 3-methylisothiazol-5-amine (**1m**) was used as a substrate, the yield was lower (25%), probably due to decreased reactivity compared to **1a.**

After studying the scope of the reaction with different isoxazol-5-amines, we turned our attention to the motif of 3,4-dihydroquinoxalin-2-one (Scheme 2). First, we wanted to know how the benzylic substituent at the aminic nitrogen affects the reactivity. To this end, 3,4-dihydroquinoxalines bearing either an electron-withdrawing (**1b**, *p*-CF_3_) or an electron-donating group (**1c**, *p*-OMe) were subjected to the reaction conditions by using **PC5**. Pleasingly, product **3bb** was generated in 96% yields, whereas product **3cb** was isolated in 66% yields. Besides, a 3,4-dihydroquinoxalin-2-one bearing a heteroaromatic benzylic substituent (**1d**, 2-thiophene) was tested, although the expected product **3db** was isolated in only 40% yields. Substitution at this position was further investigated. Compound **1e** bearing a methyl ester group was also interrogated, obtaining the corresponding product **3eb** in 41% yields. As a checkpoint, the same reaction was repeated but using **PC4** instead of **PC5**, and in this case, product **3eb** was only observed in 19% yields, confirming the superior performance of **PC5** over **PC4**. We also tested substrate **1f**, which bears a methyl substituent at N-1. To our delight, product **3fb** was isolated in a 32% yield. In the same line, *N,N*-Dibenzyl substrate **1g** delivered the expected product **3gb** in 55% yields. The substitution at the parental aromatic ring in 3,4-dihydriquinoxalin-2-ones was also interrogated. The presence of a weak electron-donating group such as a methyl substituent at C-7 afforded product **3hb** in 52% yields. In contrast, substrates bearing strongly electron-donating or weak electron-withdrawing groups at this position, such as a methoxy or a bromine atom, delivered products **3ib** and **3jb** in 78 and 70% yields, respectively. The introduction of a fluorine atom at C-6 resulted in a comparatively lower yield of **3kb** (45%). In dihydroquinoxalinones substituted at C-5, electron-donating groups (Me) afforded higher yields (58%) than electron-withdrawing substituents (Cl), with the latter providing **3mb** in only 37% yields. In this case, the reduced efficiency may also stem from increased steric hindrance in close proximity to the nitrogen atom involved in the reaction. Finally, disubstituted dihydroquinoxalinones were examined. 3,4-Dihydroquinoxalin-2-one **1n**, bearing a 6,7-dichloro substitution pattern, delivered product **3nb** in 24% yields, whereas 6,7-dimethyl-3,4-dihydroquinoxalin-2-one **1o** was converted into **3ob** in 47% yields.

**Scheme 2:**
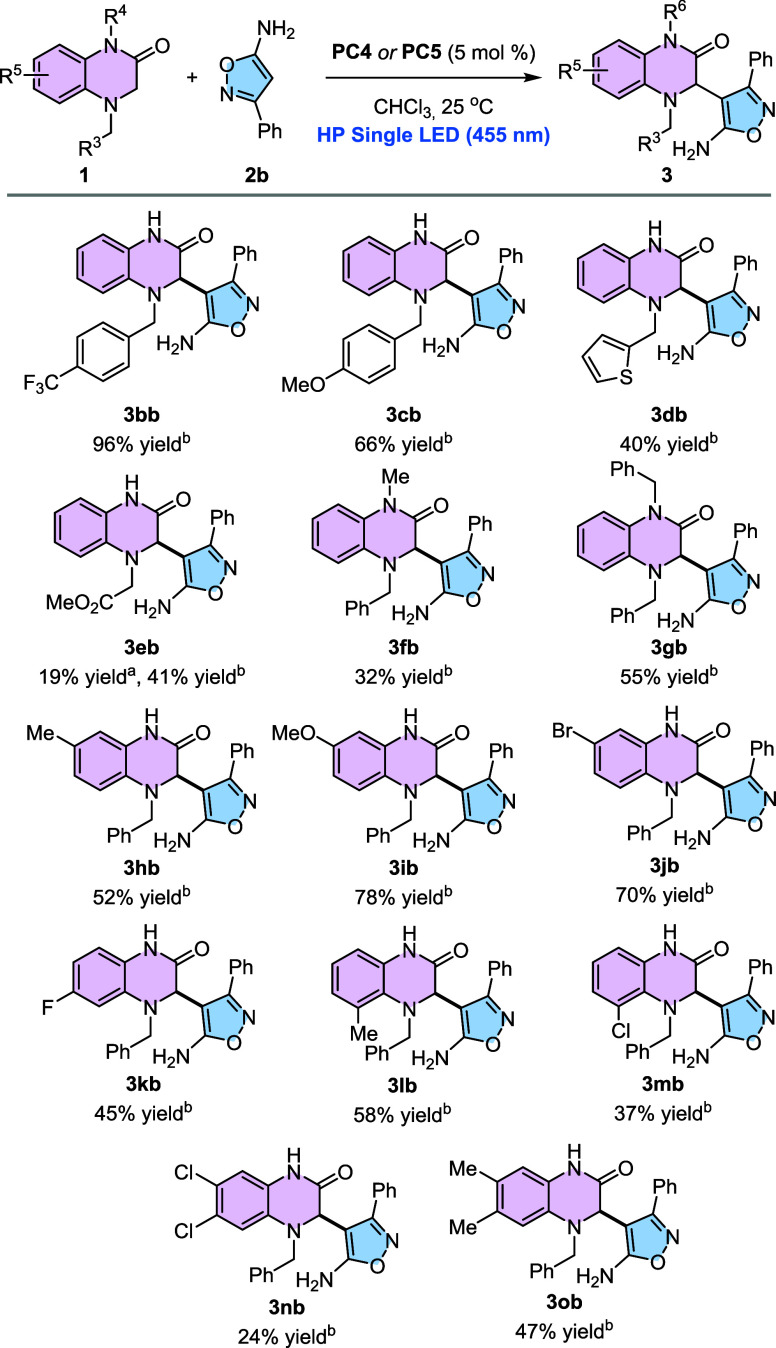
Scope of the Reaction Using Differently Substituted 3,4-Dihydroquinoxalin-2-ones^a^

a Reaction conditions: **1** (0.13 mmol), **2b** (0.1 mmol), **PC** (5 mol %), CHCl_3_ (1 mL) under the irradiation of HP single LED (455 nm). Yields were determined after purification by column chromatography.
b
**PC4** was used.
c
**PC5** was used.

After examining the scope of the reaction, we tested the scalability of the process (Scheme 3). To this end, the reaction was carried out using 1.3 mmol of **1a** and 1 mmol of **2a** and was irradiated using the same setup. When the transformation was quenched after 89 h, product **3aa** was isolated in a modest 47% yield. Seeking to improve the conversion, we extended the reaction time to 144 h. However, in this case, we observed the formation of the desired product in a lower 13% yield, whereas the overoxidation product **4** was isolated in 35% yields. This served also to identify and to fully characterize the product whose formation pathway prevents the obtention of higher yields of products **3.**

**Scheme 3:**
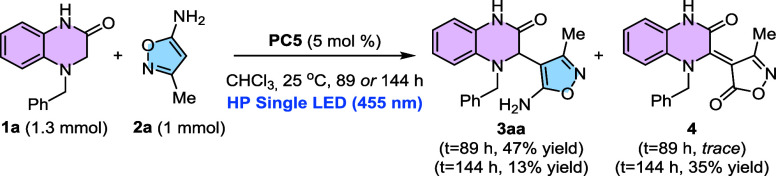
Scale-Up of the Reaction

With the aim of proposing a reaction mechanism, we performed steady-state luminescence quenching experiments to identify the species that interact with the excited state photocatalyst. However, since 9,10-phenanthrenedione (**PC5**) does not exhibit fluorescence, we performed these studies using 2,4,6-triphenylpyrylium-BF_4_ (**PC4**) as the photocatalyst (see details in the SI). Although both 3,4-dihydroquinoxalin-2-one **1a** and isoxazol-5-amine **2a** can quench the excited state of **PC4**, the latter delivers a higher Stern–Volmer constant. Consequently, under these specific conditions, it can be assumed that 3,4-dihydroquinoxaline-2-one **1a** interacts bimolecularly with the excited state of **PC4**. On the one hand, in the previous work of the research group, we have determined that the reduction potential (*E*_1/2_^0^) of **1a** is +0.77 V vs SCE.^27^ On the other hand, it is reported that the reduction potential (*E*_1/2_^0^) of **PC4** in the excited state is +2.55 V vs SCE.^8^ Consequently, the interaction between these two species is thermodynamically favorable and most likely occurs via a single-electrontransfer (SET) process. Additionally, the presence of H_2_O_2_ in the reaction mixture was confirmed by the I_2_–starch experiment (see the SI for further details).

With all of these information, including the control experiments (Table 1, entries 2, 17, and 18), we decided to propose a reaction mechanism (Figure 3). First, the SET between **PC4** and 3,4-dihydroquinoxalin-2-one **1a** results in the formation of the corresponding radical cation (**A**) of **1a** and the reduced form of the photocatalyst (**B**). Then, **B** can react via an SET with molecular oxygen to recover its initial oxidation state with the concomitant formation of the superoxide radical anion. Proton transfer from **A** to the superoxide radical anion generates α-amino radical **C** as well as the hydroperoxide radical. Finally, another SET process between **C** and the hydroperoxide radical yields the electrophilic iminium cation **D** and the hydroperoxide anion. The presence of isoxazol-5-amine **2a** in the reaction media allows the formation of the expected desired product **3aa** via an *aza*-Fridel–Crafts reaction with **D**. In the absence of an external photocatalyst, product **3aa** was obtained in 28% yields (Table 1, entry 1), consistent with observations reported by Hong^23^ and by our group.^34^ This result suggests that substrate **1a** itself could act as a photosensitizer, gradually being excited under visible-light irradiation. The excited species transfers energy to ground-state molecular oxygen (^**3**^ **O**_**2**_), producing singlet oxygen (^**1**^ **O**_**2**_) and regenerating **1a**. The generated ^**1**^ **O**_**2**_ can subsequently oxidize **1a** through a single-electron transfer process, forming a radical cation (**A**) and superoxide (**O**_**2**_^**·‑**^), which then participate in further reaction steps.

**Figure 3:**
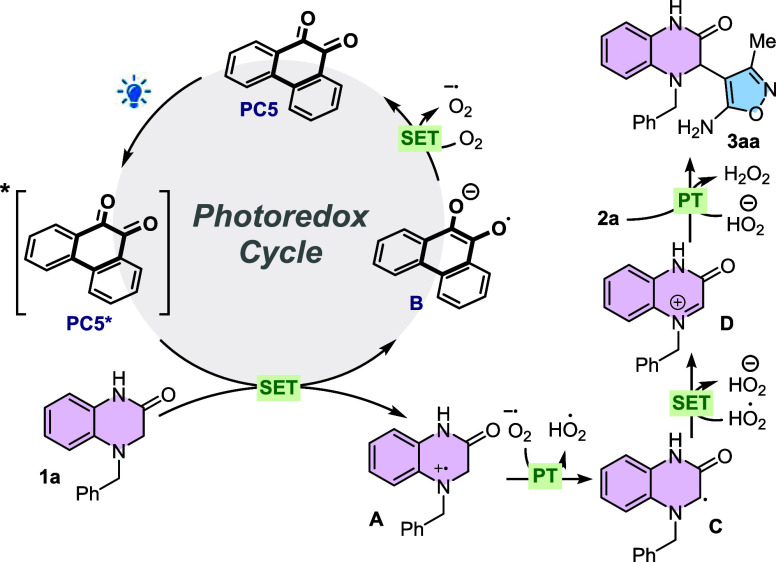
Proposed photochemical mechanism for the reaction between **1a** and **2a**.

## Conclusions

In conclusion, we have shown that isoxazol-5-amines **2** can be functionalized under visible-light photochemical conditions. Specifically, we have developed a photoredox methodology for the functionalization of 3,4-dihydroquinoxalin-2-one **1** with isoxazol-5-amine **2** using visible light as the energy source. Under the applied reaction conditions, the alkylation of isoxazol-5-amines proceeds with complete chemoselectivity, leading to C-4-substituted products. Reaction optimization revealed the occurrence of an overoxidation pathway of the desired product **3**, which significantly reduced the yields. Examination of the substrate scope demonstrated that a broad range of substituents and photosensitive functional groups is well-tolerated under the reaction conditions. The methodology was further evaluated on a larger scale, where the efficiency decreased relative to small-scale reactions due to the need for prolonged irradiation times, which in turn promoted the formation of the overoxidized byproduct **4**. Further studies to extend the applicability of isoxazol-5-amine **2** in visible-light photochemistry are currently underway in our laboratory.

## Supplementary Material



## Data Availability

The data underlying this study are available in the published article, in its Supporting Information, and openly available in DRYAD at 10.5061/dryad.rfj6q57pz.

## Notes

Experimental procedures, characterization data, and NMR spectra (PDF)

## References

[1] Visible Light Photocatalysis in Organic Chemistry; Stephenson, C. R. J. ; Yoon, T. P. , Eds.; Wiley-VCH Verlag GmbH & Co: Weinheim, Germany, 2018.

[2] Prier C. K., Rankic D. A., MacMillan D. W. C. (2013). Visible
Light Photoredox Catalysis
with Transition Metal Complexes: Applications in Organic Synthesis. Chem. Rev..

[3] Shaw M. H., Twilton J., MacMillan D. W. C. (2016). Photoredox
Catalysis in Organic Chemistry. J. Org. Chem..

[4] Marzo L., Pagire S. K., Reiser O., König B. (2018). Visible-Light
Photocatalysis: Does It Make a Difference in Organic Synthesis?. Angew. Chem., Int. Ed..

[5] Schultz D. M., Yoon T. P. (2014). Solar Synthesis:
Prospects in Visible Light Photocatalysis. Science.

[6] Akita M., Ceroni P., Stephenson C. R. J., Masson G. (2023). Progress in Photocatalysis
for Organic Chemistry. J. Org. Chem..

[7] Hoffmann N. (2008). Photochemical
Reactions as Key Steps in Organic Synthesis. Chem. Rev..

[8] Romero N. A., Nicewicz D. A. (2016). Organic Photoredox Catalysis. Chem. Rev..

[9] Buzzetti L., Crisenza G. E. M., Melchiorre P. (2019). Mechanistic
Studies in Photocatalysis. Angew. Chem., Int.
Ed..

[10] Hu J., Wang J., Nguyen T. H., Zheng N. (2013). The Chemistry of Amine
Radical Cations Produced by Visible Light Photoredox Catalysis. Beilstein J. Org. Chem..

[11] Huang C., Ye Z.-M., Qin Y.-S., You G.-P., Wei Z., Cai H. (2025). Radical α-C–H Alkylation and Heteroarylation of Benzyl
Anilines Enabled by Organic Photoredox Catalysis. Org. Lett..

[12] Beatty J. W., Stephenson C. R. J. (2015). Amine Functionalization via Oxidative
Photoredox Catalysis:
Methodology Development and Complex Molecule Synthesis. Acc. Chem. Res..

[13] Freeman D. B., Furst L., Condie A. G., Stephenson C. R. J. (2012). Functionally
Diverse Nucleophilic Trapping of Iminium Intermediates Generated Utilizing
Visible Light. Org. Lett..

[14] Condie A. G., González-Gómez J. C., Stephenson C. R. J. (2010). Visible-Light
Photoredox Catalysis: Aza-Henry Reactions via C–H Functionalization. J. Am. Chem. Soc..

[15] Rueping M., Vila C., Koenigs R. M., Poscharny K., Fabry D. C. (2011). Dual Catalysis: Combining Photoredox
and Lewis Base
Catalysis for Direct Mannich Reactions. Chem.
Commun..

[16] Pan Y., Kee C. W., Chen L., Tan C.-H. (2011). Dehydrogenative
Coupling Reactions Catalysed by Rose Bengal Using Visible Light Irradiation. Green Chem..

[17] Miyake Y., Nakajima K., Nishibayashi Y. (2012). Visible-Light-Mediated Utilization
of α-Aminoalkyl Radicals: Addition to Electron-Deficient Alkenes
Using Photoredox Catalysts. J. Am. Chem. Soc..

[18] Kohls P., Jadhav D., Pandey G., Reiser O. (2012). Visible Light Photoredox
Catalysis: Generation and Addition of *N*-Aryltetrahydroisoquinoline-Derived
α-Amino Radicals to Michael Acceptors. Org. Lett..

[19] Holmberg-Douglas N., Nicewicz D. A. (2022). Photoredox-Catalyzed C-H Functionalization Reactions. Chem. Rev..

[20] Kristoffersen T., Hansen J. H. (2017). 3,4-Dihydroquinoxalin-2-Ones:
Recent Advances in Synthesis
and Bioactivities (Microreview). Chem. Heterocycl.
Compd..

[21] Lattanzi A. (2022). 3,4-Dihydroquinoxalin-2-One
Privileged Motif: A Journey from Classical Chiral Tools Based Synthesis
to Modern Catalytic Enantioselective Strategies. Tetrahedron Chem..

[22] Tang A. H., Franklin S. R., Himes C. S., Ho P. M. (1991). Behavioral
Effects
of U-78875, a Quinoxalinone Anxiolytic with Potent Benzodiazepine
Antagonist Activity. J. Pharmacol. Exp. Ther..

[23] Akula P. S., Hong B.-C., Lee G.-H. (2018). Visible-Light-Induced C­(Sp3)–H
Activation for a C-C Bond Forming Reaction of 3,4-Dihydroquinoxalin-2­(1
H)-One with Nucleophiles Using Oxygen with a Photoredox Catalyst or
under Catalyst-Free Conditions. RSC Adv..

[24] Rostoll-Berenguer J., Blay G., Muñoz M. C., Pedro J. R., Vila C. (2019). A Combination
of Visible-Light Organophotoredox Catalysis and Asymmetric Organocatalysis
for the Enantioselective Mannich Reaction of Dihydroquinoxalinones
with Ketones. Org. Lett..

[25] Rostoll-Berenguer J., Blay G., Pedro J. R., Vila C. (2023). Catalytic Nucleophilic
and Electrophilic Functionalization of Dihydroquinoxalin-2-ones. ChemCatChem.

[26] Xiong W., Qin W.-B., Zhao Y.-S., Fu K.-Z., Liu G.-K. (2022). Direct
C­(Sp3)–H Difluoromethylation via Radical-Radical Cross-Coupling
by Visible-Light Photoredox Catalysis. Org.
Chem. Front..

[27] Rostoll-Berenguer J., Blay G., Pedro J. R., Vila C. (2020). Photocatalytic Giese
Addition of 1,4-Dihydroquinoxalin-2-Ones to Electron-Poor Alkenes
Using Visible Light. Org. Lett..

[28] Hota S. K., Singh G., Murarka S. (2024). Direct C-H alkylation of 3,4-dihydroquinoxaline-2-ones
with N-(acyloxy)­phthalimides via radical–radical cross coupling. Chem. Commun..

[29] Rostoll-Berenguer J., Martín-López M., Blay G., Pedro J. R., Vila C. (2022). Radical Addition of Dihydroquinoxalin-2-Ones to Trifluoromethyl Ketones
under Visible-Light Photoredox Catalysis. J.
Org. Chem..

[30] Rostoll-Berenguer J., García-García V., Blay G., Pedro J. R., Vila C. (2023). Organophotoredox 1,6-Addition of 3,4-Dihydroquinoxalin-2-Ones to *Para* -Quinone Methides Using Visible Light. ACS Org. Inorg. Au.

[31] Pan C., Chen D. (2024). Photocatalytic Consecutive
Photoinduced Electron Transfer-Enabled
C­(Sp3)–H Pyridylation of Dihydroquinoxalin-2-Ones. J. Org. Chem..

[32] Jiang Y.-F., Ouyang W.-T., Ji H.-T., Hou J.-C., Li T., Luo Q.-X., Wu C., Ou L.-J., He W.-M. (2024). Phototriggered
Self-Catalyzed Phosphorylation of 3,4-Dihydroquinoxalin-2­(1 *H*)-Ones with Diarylphosphine Oxides in EtOH. J. Org. Chem..

[33] Jia L., Lu Y., Chen Y., Zhong Y., Zhao F., Zhou Y. (2024). Visible-Light-Induced
Metal- and Photosensitizer-Free C­(Sp3)–H Phosphorylation of
3,4-Dihydroquinoxalin-2­(1 H)-Ones with Diphenylphosphine Oxide. J. Org. Chem..

[34] Rostoll-Berenguer J., Sierra-Molero F. J., Blay G., Pedro J. R., Vila C. (2022). Photocatalytic
Functionalization of Dihydroquinoxalin-2-Ones with Pyrazolones. Adv. Synth. Catal..

[35] Hamama W. S., Ibrahim M. E., Zoorob H. H. (2013). Advances
in the Chemistry of Aminoisoxazole. Synth. Commun..

[36] Zhu J., Mo J., Lin H., Chen Y., Sun H. (2018). The Recent Progress
of Isoxazole in Medicinal Chemistry. Bioorg.
Med. Chem..

[37] Sysak A., Obmińska-Mrukowicz B. (2017). Isoxazole Ring as a
Useful Scaffold
in a Search for New Therapeutic Agents. Eur.
J. Med. Chem..

[38] Barmade M. A., Murumkar P. R., Kumar Sharma M., Ram Yadav M. (2016). Medicinal
Chemistry Perspective of Fused Isoxazole Derivatives. Curr. Top. Med. Chem..

[39] Blay G., Monleón A., Montesinos-Magraner M., Sanz-Marco A., Vila C. (2024). Asymmetric electrophilic
functionalization of amino-substituted heteroaromatic
compounds: a convenient tool for the enantioselective synthesis of
nitrogen heterocycles. Chem. Commun..

[40] Yan Y., Bao A., Wang Y., Xie X., Wang D., Deng Z., Wang X., Cheng W., Li W., Zhang X., Tang X. (2024). Design, Synthesis, Antifungal Activity,
and Molecular Docking Studies
of Novel Chiral Isoxazoline-Benzofuran-Sulfonamide Derivatives. J. Agric. Food Chem..

[41] Yan Y., Bao A., Li M., Xie X., Li W., Zhang X. (2023). Highly Enantioselective
[3 + 2] Annulation of 4-Amino-Isoxazoles with Quinone Monoimines to
Access Structurally Diverse Isoxazoline Fused Dihydrobenzofurans and
Antifungal Evaluation. J. Mol. Struct..

[42] Cabanne F., Lefebvre A., Scalla R. (1987). Behaviour
of the Herbicide EL-107
in Wheat and Rape Grown under Controlled Conditions. Weed Res..

[43] Vaughn, K. C. Cellulose Biosynthesis Inhibitor Herbicides. In Herbicide Classes in Development; Böger, P. ; Wakabayashi, K. ; Hirai, K. , Eds.; Springer Berlin Heidelberg: Berlin, Heidelberg, 2002; pp 139–150.

[44] Ge Y., Sun W., Pei B., Ding J., Jiang Y., Loh T.-P. (2018). Hoveyda-Grubbs
II Catalyst: A Useful Catalyst for One-Pot Visible-Light-Promoted
Ring Contraction and Olefin Metathesis Reactions. Org. Lett..

[45] Li J., Zhu D., Lv L., Li C.-J. (2018). Radical Difluoromethylthiolation
of Aromatics Enabled by Visible Light. Chem.
Sci..

[46] Ali D., Parvin T., Choudhury L. H. (2022). Visible Light-Mediated C­(Sp^2^)–H Selenylation of Amino Pyrazole and Amino Uracils in the
Presence of Rose Bengal as an Organophotocatalyst. J. Org. Chem..

