# Supplementary material for: Organophotocatalytic Functionalization of 3,4-Dihydroquinoxalin-2-ones with Isoxazol-5-amines Using Visible Light
Source: J Org Chem. 2025 Dec 9;90(50):18045–51. doi: 10.1021/acs.joc.5c02592 (PMC12723677; doi:10.1021/acs.joc.5c02592)

Organophotocatalytic Functionalization of 3,4-Dihydroquinoxalin-2-ones with Isoxazol-5-amines using visible light

Salma E. Mora-Rodríguez[ab], Jaume Rostoll-Berenguer[a], Selene Lagunas[c], Miguel A. Vázquez[b], Gonzalo Blay[a]* José R. Pedro[a] and Carlos Vila[a]*

[a]*Departament de Química Orgànica, Facultat de Química, Universitat de València, Dr. Moliner 50, 46100 Burjassot, Spain*

[b]*Departamento de Química, Universidad de Guanajuato, Noria Alta s/n, 36050 Guanajuato, Mexico*

[c]*ImX-CONACyT. Departamento de Química, Universidad de Guanajuato, Noria Alta s/n, Gto. 36050 Guanajuato, Mexico*

Carlos.Vila@uv.es

Gonzalo.Blay@uv.es

Table of contents

General Experimental Methods S2
Synthetic Procedures S3
Characterization of the Products S5
Mechanistic Investigations S19
Unsuccessful examples S22
References S23
NMR Spectra S24

General Experimental Methods

Unless otherwise noted, reactions were carried out in regular 5 mL-vials. Reactions were monitored by TLC analysis using Merck Silica Gel 60 F-254 thin layer plates. Flash column chromatography was performed on Merck silica gel 60, 0.040-0.063 mm.

NMR spectra were run at 300 MHz (Bruker Avance III 300) for ^{1}H and at 75 MHz for ^{13}C NMR using residual non-deuterated solvent as internal standard ($CDCl_3$: 7.26 and 77.00 ppm respectively; DMSO-d_6: 2.50 and 39.52 ppm respectively, CD_3OD: 3.31 and 49.00 ppm, respectively) and at 282 MHz for ^{19}F NMR. In some spectra recorded in DMSO-d_6, a singlet appears at 8.31 ppm, corresponding to residual $CHCl_3$. Chemical shifts are given in ppm. The carbon type was determined by DEPT experiments.

High resolution mass spectra (ESI) were recorded on a AB SCIEX Triple TOFTM spectrometer equipped with an electrospray source with a capillary voltage of 4.5 kV(ESI).

3,4-Dihydroquinoxalin-2-ones **1** were prepared following reported procedures.[1,2] Isoxazol-5-amines **2** and photocatalysts were purchased and used as received. *N*-(3-methylisoxazol-5-yl)acetamide, *N*-ethyl-3-methylisoxazol-5-amine, *N*-ethyl-*N*,3-dimethylisoxazol-5-amine were prepared following reported procedures.[3,4]

Photochemical Setup: HP Single LED (455 nm). – Description: All the components for the photochemical setup were purchased from Farnell Electronics. Five HP Single LEDs (455 nm) (int. ref. 3583117), assembled with a graphite heat sink pad (int. ref. 3583131), were stuck over an extruded aluminium heat sink (int. ref. 4621931) using thermally conductive epoxy adhesive (int. ref. 2917612). DC power supply was provided by Bench Power Supply (int. ref. 3410526). The setup is placed in a thermostat controlled room and the heat is dissipated using a fan.

Synthetic Procedures

Synthesis of *N*-ethyl-*N*,3-dimethylisoxazol-5-amine (2l) [4]

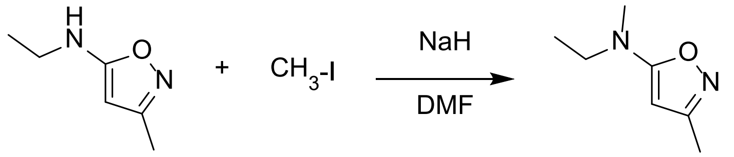

To a suspension of NaH (0.114 g, 60% in oil, 2.85 mmol) in DMF (4 mL) at 0 ℃ was added N-ethyl-3-methylisoxazol-5-amine **2k** (0.150 g, 1.19 mmol) and stirred for 30 min. After 30 minutes, iodomethane (0.111mL, 1.78 mmol) was added at 0 ℃ and the reaction mixture was stirred overnight at room temperature. After completion of the reaction, the mixture was quenched with water and extracted with EtOAc. The combined organic layer was washed with brine, dried over anhydrous Na_2SO_4, filtered, and evaporated under reduced pressure. The crude product was then purified by column chromatography using hexane/ethyl acetate (from 95:05 to 80:20) as eluents, affording the desired pure compound **2l** (colorless oil, 105.2 mg, 0.75 mmol, 63% yield).

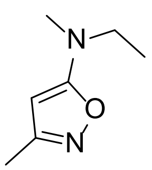

^{1}H NMR (300 MHz, $CDCl_3$) δ 4.71 (s, 1H), 3.32 (q, *J* = 7.1 Hz, 2H), 2.91 (s, 3H), 2.15 (s, 3H), 1.15 (t, *J* = 7.1 Hz, 3H) ppm.

^{13}C {1H} NMR (75 MHz, $CDCl_3$) δ 170.4 (C), 161.6 (C), 77.2 (CH), 45.9 (CH_2), 35.6 (CH_3), 11.9 (CH_3), 11.8 (CH_3) ppm.

HRMS (ESI+) *m/z:* $[M + H]^+$ Calcd for $C_7H_{13}N_2O^+$ 141.1022; found 141.1025.

Functionalization of 3,4-Dihydroquinoxalin-2-ones with Isoxazol-5-amines

1. General Procedure 1 (GP-1) for the Functionalization of 3,4-Dihydroquinoxalin-2-ones with Isoxazol-5-amines at 0.1 mmol scale.

In a 10 mL test tube, the corresponding 3,4-dihydroquinoxalin-2-one (**1**, 0.13 mmol, 1.3 equiv.), the corresponding isoxazole-5-amine (**2**, 0.10 mmol, 1.0 equiv.), and 9,10-phenanthrenequinone (**PC5**, 0.005 mmol, 5 mol %) were combined. Chloroform (1 mL, 0.1 M) was then added, and the reaction mixture was positioned 1 cm above a high-power single LED (455 nm). The progress of the reaction was monitored periodically by thin-layer chromatography (TLC). Upon complete consumption of the 3,4-dihydroquinoxalin-2-one **1**, irradiation was stopped, and the mixture was concentrated under reduced pressure. The crude product was then purified by column chromatography using either hexane/ethyl acetate (from 90:10 to 70:30) or dichloromethane/ethyl acetate (from 90:10 to 60:40) as eluents, affording the desired pure compound **3**.

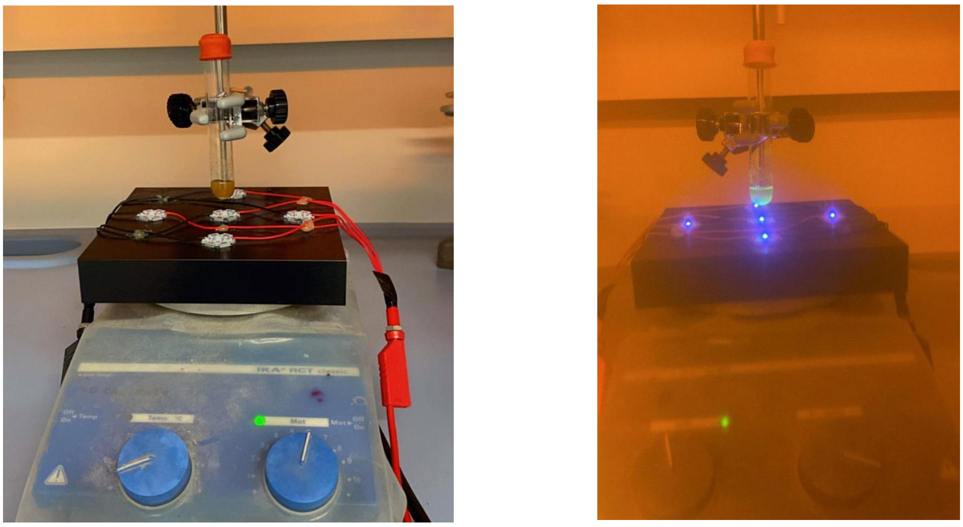

Figure S1: Typical reaction setup.

2. Specific Procedure (SP) Functionalization of 3,4-Dihydroquinoxalin-2-one **1a** with Isoxazol-5-amine **2a** at 1 mmol scale.

In a 50 mL round bottom flask, 3,4-dihydroquinoxalin-2-one **1a** (1.3 mmol, 1.3 equiv., 310 mg), isoxazol-5-amine **2a** (1 mmol, 1 equiv., 98 mg), 9,10-phenanthrenequinone (**PC5**, 5 mol %, 10 mg) are added. Next, the reaction mixture is dissolved in $CHCl_3$ (10 mL, 0.1 M) and stirred under the irradiation of a HP Single LED (455 nm) while being cooled with a fan to keep the temperature at approximately 25 ^{o}C. The progress of reaction was monitored by TLC and directly purified by column chromatography using mixtures of hexane:AcOEt as eluent to obtain the desired product **3aa** and also the oxidation product **4**. When the reaction time was 144 hours, **3aa** was obtained in 13% yield (43.6 mg, 0.13 mmol) and **4** was obtained in 35% yield (116.9 mg, 0.35 mmol). When the reaction time was 89 h, **3aa** was obtained in 47% yield (157.4 mg, 0.47 mmol).

Characterization of the Products

3-(5-Amino-3-methylisoxazol-4-yl)-4-benzyl-3,4-dihydroquinoxalin-2(1*H*)-one (3aa).

Using 4-benzyl-3,4-dihydroquinoxalin-2-one (**1a**, 31.0 mg, 0.13 mmol, 1.3 equiv.) and 3-methylisoxazol-5-amine (**2a**, 9.8 mg, 0.1 mmol, 1 equiv.), according to GP-1, compound **3aa** (orange oil) was purified by column chromatography using hexane:EtOAc mixtures (from 8:2 to 5:5). It was obtained in 54% yield (18.1 mg, 0.054 mmol) using **PC4** and in 47% yield (15.9 mg, 0.047 mmol) using **PC5**.

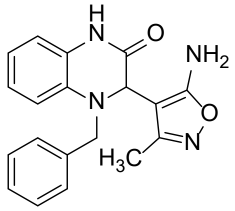

^{1}H NMR (300 MHz, CDCl$_3$) δ 8.41 (s, 1H), 7.45 – 7.20 (m, 5H), 7.09 – 6.93 (m, 1H), 6.87 – 6.76 (m, 3H), 4.85 – 4.66 (m, 2H), 4.19 (s, 2H), 4.06 (d, *J* = 15.0 Hz, 1H), 1.87 (s, 3H).

^{13}C{^{1}H} NMR (75 MHz, CDCl$_3$) δ 166.2 (C), 166.0 (C), 160.7 (C), 135.6 (C), 134.2 (C), 128.9 (CH), 127.9 (CH), 125.0 (CH), 124.8 (C), 119.4 (CH), 116.0 (CH), 112.2 (CH), 89.4 (C), 55.2 (CH), 51.1 (CH$_2$), 10.9 (CH$_3$) ppm.

HRMS (ESI+) *m/z:* [M + H]$^+$ Calcd for $C_{19}H_{19}N_4O_2^+$ 335.1503; found 335.1491.

3-(5-Amino-3-phenylisoxazol-4-yl)-4-benzyl-3,4-dihydroquinoxalin-2(1*H*)-one (3ab)

Using 4-benzyl-3,4-dihydroquinoxalin-2-one (**1a**, 31.0 mg, 0.13 mmol, 1.3 equiv.) and 3-phenylisoxazol-5-amine (**2b**, 16 mg, 0.1 mmol, 1 equiv.), according to GP-1, compound **3ab** (amorphous yellow solid) was purified by column chromatography using hexane:EtOAc mixtures (from 8:2 to 5:5). It was obtained in 55% yield (21.9 mg, 0.0554 mmol) using **PC4** and in 55% yield (21.7 mg, 0.0548 mmol) using **PC5**.

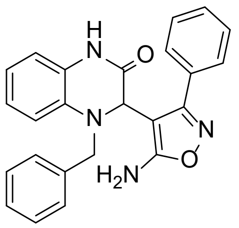

^{1}H NMR (300 MHz, CDCl$_3$) δ 9.15 (s, 1H), 7.62 – 7.55 (m, 2H), 7.38 – 7.30 (m, 3H), 7.16 – 7.09 (m, 3H), 7.15-7.11 (m, 2H), 6.93 (ddd, *J* = 8.1, 7.0, 2.0 Hz, 1H), 6.85 – 6.74 (m, 2H), 6.63 (d, *J* = 8.1 Hz, 1H), 4.96 (s, 1H), 4.50 (d, *J* = 15.4 Hz, 1H), 4.29 (s, 2H), 3.93 (d, *J* = 15.4 Hz, 1H) ppm.

^{13}C{^{1}H} NMR (75 MHz, CDCl$_3$) δ 166.9 (C), 166.0 (C), 164.5 (C), 135.6 (C), 134.1 (C), 129.4 (CH), 128.9 (C), 128.8 (CH), 128.6 (CH), 128.5 (CH), 127.4 (CH), 127.4 (CH), 125.0 (CH), 124.5 (C), 119.3 (CH), 115.8 (CH), 112.3 (CH), 89.7 (C), 54.8 (CH), 50.8 (CH$_2$) ppm.

HRMS (ESI+) *m/z:* [M + H]$^+$ Calcd for $C_{24}H_{21}N_4O_2^+$ 397.1659; found 397.1647.

3-(5-Amino-3-ethylisoxazol-4-yl)-4-benzyl-3,4-dihydroquinoxalin-2(*1H*)-one (3ac).

Using 4-benzyl-3,4-dihydroquinoxalin-2-one (**1a**, 31.0 mg, 0.13 mmol, 1.3 equiv.) and 3-ethylisoxazol-5-amine (**2c**, 11 mg, 0.1 mmol, 1 equiv.), according to GP-1, compound **3ac** (brown oil) was purified by column chromatography using hexane:EtOAc mixtures (from 8:2 to 5:5). It was obtained in 43% yield (15.1 mg, 0.0432 mmol) using **PC4** and in 70% yield using **PC5** (24.4 mg, 0.07 mmol).

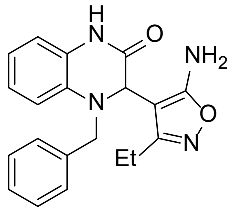

^{1}H NMR (300 MHz, $CDCl_3$) δ 9.07 (s, 1H), 7.41 – 7.19 (m, 5H), 6.99 (ddd, *J* = 8.9, 5.7, 3.2 Hz, 1H), 6.85 – 6.73 (m, 3H), 4.76 (s, 1H), 4.71 (d, *J* = 15.2 Hz, 1H), 4.15 (s, 2H), 4.07 (d, *J* = 15.2 Hz, 1H), 2.47 – 2.12 (m, 2H), 1.12 (t, *J* = 7.5 Hz, 3H).

$^{13}C\{^1H\}$ NMR (75 MHz, $CDCl_3$) δ 166.3 (C), 166.1 (C), 165.6 (C), 135.9 (C), 134.3 (C), 129.1 (CH), 128.0 (CH), 127.9 (CH), 125.1 (CH), 124.9 (C), 119.6 (CH), 116.1 (CH), 112.3 (CH), 89.1 (C), 55.3 (CH), 51.2 (CH_2), 19.2 (CH_2), 11.6 (CH_3).

HRMS (ESI+) *m/z:* $[M + H]^+$ Calcd for $C_{20}H_{21}N_4O_2^+$ 349.1659; found 349.1674.

3-(5-Amino-3-propylisoxazol-4-yl)-4-benzyl-3,4-dihydroquinoxalin-2(*1H*)-one (3ad)

Using 4-benzyl-3,4-dihydroquinoxalin-2-one (**1a**, 31.0 mg, 0.13 mmol, 1.3 equiv.) and 3-propylisoxazol-5-amine (**2d**, 12.6 mg, 0.1 mmol, 1 equiv.), according to GP-1, compound **3ad** (amorphous orange solid) was purified by column chromatography using hexane:EtOAc mixtures (from 8:2 to 5:5). It was obtained in 54% yield (20.2 mg, 0.054 mmol) using **PC4** and in 60% yield (21.7 mg, 0.06 mmol) using **PC5**.

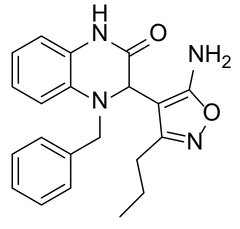

^{1}H NMR (300 MHz, CD_3OD) δ 7.46 – 7.18 (m, 5H), 6.99 – 6.86 (m, 2H), 6.81 – 6.65 (m, 2H), 4.98 (s, 1H), 4.62 (d, *J* = 16.1 Hz, 1H), 4.20 (d, *J* = 16.1 Hz, 1H), 2.24 – 2.05 (m, 2H), 1.55 – 1.32 (m, 2H), 0.75 (t, *J* = 7.4 Hz, 3H).

$^{13}C\{^1H\}$ NMR (75 MHz, CD_3OD) δ 170.0 (C), 167.9 (C), 165.0 (C), 138.4 (C), 135.6 (C), 129.7 (CH), 128.4 (CH), 128.3 (CH), 126.8 (C), 125.4 (CH), 119.9 (CH), 116.7 (CH), 113.8 (CH), 89.4 (C), 57.0 (CH), 52.2 (CH_2), 28.6 (CH_2), 22.0 (CH_2), 14.3 (CH_3).

HRMS (ESI+) *m/z:* $[M + H]^+$ Calcd for $C_{21}H_{23}N_4O_2^+$ 363.1816; found 363.1814.

3-(5-Amino-3-isopropylisoxazol-4-yl)-4-benzyl-3,4-dihydroquinoxalin-2(1*H*)-one (3ae)

Using 4-benzyl-3,4-dihydroquinoxalin-2-one (**1a**, 31.0 mg, 0.13 mmol, 1.3 equiv.) and 3-isopropylisoxazol-5-amine (**2e**, 12.6 mg, 0.1 mmol, 1 equiv.), according to GP-1, compound **3ae** (amorphous orange solid) was purified by column chromatography using hexane:EtOAc mixtures (from 8:2 to 5:5). It was obtained in 63% yield (23.7 mg, 0.063 mmol) using **PC4** and in 63% yield (23.5 mg, 0.063 mmol) using **PC5**.

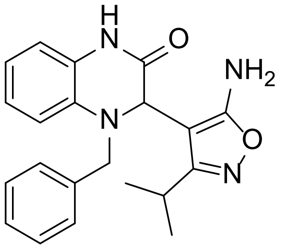

^{1}H NMR (300 MHz, $CDCl_3$) δ 9.47 (s, 1H), 7.37 – 7.23 (m, 6H), 6.98 (ddd, *J* = 7.9, 6.8, 2.2 Hz, 1H), 6.85 – 6.75 (m, 3H), 4.83 (s, 1H), 4.71 (d, *J* = 15.5 Hz, 1H), 4.14 – 4.07 (m, 3H), 2.58 (hept, *J* = 6.9 Hz, 1H), 1.24 (d, *J* = 6.9 Hz, 3H), 1.13 (d, *J* = 6.9 Hz, 3H) ppm.

^{13}C {1H} NMR (75 MHz, $CDCl_3$) δ 169.4 (C), 166.0 (C), 166.0 (C), 135.8 (C), 134.16 (C), 128.9 (CH), 127.7 (CH), 127.5 (CH), 125.0 (C), 124.8 (CH) , 119.4 (CH), 116.0 (CH), 112.2 (C), 88.5 (CH), 55.1 (CH), 50.9 (CH_2), 25.8 (CH), 21.8 (CH_3), 21.0 (CH_3) ppm.

HRMS (ESI+) *m/z:* $[M + H]^+$ Calcd for $C_{21}H_{23}N_4O_2^+$ 363.1816; found 363.1799.

3-(5-Amino-3-(3-methoxyphenyl)isoxazol-4-yl)-4-benzyl-3,4-dihydroquinoxalin-2(1*H*)-one (3af)

Using 4-benzyl-3,4-dihydroquinoxalin-2-one (**1a**, 31.0 mg, 0.13 mmol, 1.3 equiv.) and (3-methoxyphenyl)isoxazol-5-amine (**2f**, 19 mg, 0.1 mmol, 1 equiv.), according to GP-1, compound **3af** (brown oil) was purified by column chromatography using hexane:EtOAc mixtures (from 8:2 to 5:5). It was obtained in 36% yield (15.3 mg, 0.036 mmol) using **PC4** and in 80% yield (34.2 mg, 0.08 mmol) using **PC5**.

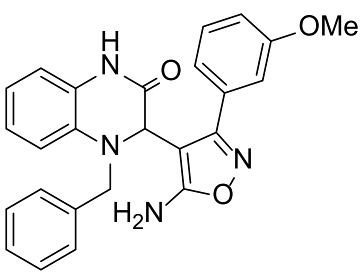

^{1}H NMR (300 MHz, $CDCl_3$) δ 9.77 (s, 1H), 7.29 – 7.21 (m, 3H), 7.16 – 7.09 (m, 3H), 7.09 – 7.01 (m, 2H), 6.96 – 6.88 (m, 2H), 6.83 (dd, *J* = 7.7, 1.7 Hz, 1H), 6.77 (dd, *J* = 7.4, 1.2 Hz, 1H), 6.64 (dd, *J* = 8.2, 1.2 Hz, 1H), 5.00 (s, 1H), 4.51 (d, *J* = 15.3 Hz, 1H), 4.30 (s, 2H), 3.94 (d, *J* = 15.3 Hz, 1H), 3.76 (s, 3H) ppm.

^{13}C{^{1}H} NMR (75 MHz, $CDCl_3$) δ 167.0 (C), 166.3 (C), 164.4 (C), 159.5 (C), 135.6 (C), 134.1 (C), 130.1 (C), 129.6 (CH), 128.6 (CH), 127.4 (CH), 125.0 (CH), 124.6 (C), 121.2 (CH), 119.3 (CH), 116.1 (CH), 116.0 (CH), 113.7 (CH), 112.3 (CH), 89.7 (C), 55.3 (CH_3), 54.7 (CH), 50.9 (CH_2), 29.7 (CH_2) ppm.

HRMS (ESI+) *m/z:* $[M + H]^+$ Calcd for $C_{25}H_{23}N_4O_3^+$ 427.1765; found 427.1746.

3-(5-Amino-3-(4-methoxyphenyl)isoxazol-4-yl)-4-benzyl-3,4-dihydroquinoxalin-2(*1H*)-one (3ag)

Using 4-benzyl-3,4-dihydroquinoxalin-2-one (**1a**, 31.0 mg, 0.13 mmol, 1.3 equiv.) and (3-methoxyphenyl)isoxazol-5-amine (**2g**, 19 mg, 0.1 mmol, 1 equiv.) and **PC5**, according to GP-1, compound **3ag** (brown oil, 70% yield, 29.8 mg, 0.07 mmol) was purified by column chromatography using hexane:EtOAc mixtures (from 8:2 to 5:5).

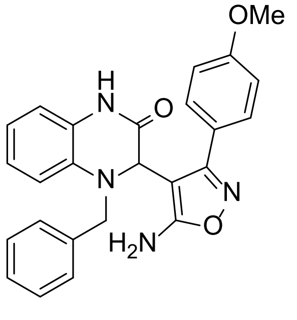

^{1}H NMR (300 MHz, DMSO-*d*$_6$) δ 10.54 (s, 1H), 7.33 (d, *J* = 8.8 Hz, 2H), 7.24 – 7.07 (m, 5H), 6.78 (d, *J* = 8.8 Hz, 2H), 6.74 – 6.65 (m, 2H), 6.62-6.57 (m, 1H), 6.52 (s, 1H), 6.44 (d, *J* = 7.8 Hz, 1H), 5.09 (s, 1H), 4.44 (d, *J* = 16.4 Hz, 1H), 4.10 (d, *J* = 16.4 Hz, 1H), 3.73 (s, 3H).

^{13}C{^{1}H} NMR (75 MHz, DMSO) δ 169.6 (C), 165.9 (C), 163.0 (C), 160.2 (C), 138.1 (C), 134.5 (C), 129.8 (CH), 128.7 (CH), 127.2 (CH), 127.1 (CH), 126.5 (C), 123.4 (CH), 122.3 (C), 118.6 (CH), 115.4 (C), 113.9 (CH), 113.0 (CH), 87.5 (C), 56.46 (CH), 55.57 (CH_3), 51.1 (CH_2).

HRMS (ESI+) *m/z:* $[M + H]^+$ Calcd for $C_{25}H_{23}N_4O_3^+$ 427.1765; found 427.1773.

3-(5-Amino-3-(*p*-tolyl)isoxazol-4-yl)-4-benzyl-3,4-dihydroquinoxalin-2(1*H*)-one (3ah)

Using 4-benzyl-3,4-dihydroquinoxalin-2-one (**1a**, 31.0 mg, 0.13 mmol, 1.3 equiv.) and 3-(*p*-tolyl)isoxazol-5-amine (**2h**, 17.4 mg, 0.1 mmol, 1 equiv.) and **PC5**, according to GP-1, compound **3ah** (amorphous orange solid, 60% yield, 24.7 mg, 0.06 mmol) was purified by column chromatography using hexane:EtOAc mixtures (from 8:2 to 5:5).

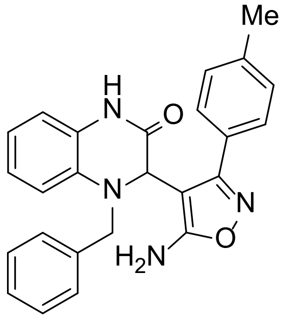

^{1}H NMR (300 MHz, $CDCl_3$) δ 9.39 (s, 1H), 7.49 (d, *J* = 8.0 Hz, 2H), 7.12 (d, *J* = 7.8 Hz, 5H), 7.04 (d, *J* = 5.3 Hz, 2H), 6.92 (t, *J* = 7.5 Hz, 1H), 6.82-6.75 (m, 2H), 6.62 (d, *J* = 8.0 Hz, 1H), 4.98 (s, 1H), 4.49 (d, *J* = 15.5 Hz, 1H), 4.25 (s, 2H), 3.93 (d, *J* = 15.5 Hz, 1H), 2.36 (s, 3H).

^{13}C{^{1}H} NMR (75 MHz, $CDCl_3$) δ 166.8 (C), 166.1 (C) 164.5 (C), 139.4 (C), 135.7 (C), 134.1 (C), 129.2 (CH), 128.7 (CH), 128.6 (CH), 127.3 (CH), 127.3 (CH), 125.9 (C), 124.9 (CH), 124.5 (C), 119.2 (CH), 115.8 (CH), 112.3 (CH), 89.7 (C), 54.9 (CH), 50.8 (CH_2), 21.3 (CH_3).

HRMS (ESI+) *m/z:* $[M + H]^+$ Calcd for $C_{25}H_{23}N_4O_2^+$ 411.1816; found 411.1823.

3-(5-Amino-3-(4-chlorophenyl)isoxazol-4-yl)-4-benzyl-3,4-dihydroquinoxalin-2(1*H*)-one (3ai)

Using 4-benzyl-3,4-dihydroquinoxalin-2-one (**1a**, 31.0 mg, 0.13 mmol, 1.3 equiv.) and 3-(4-chlorophenyl)isoxazol-5-amine (**2i**, 19.4 mg, 0.1 mmol, 1 equiv.) and **PC5**, according to GP-1, compound **3ai** (amorphous white solid 77% yield, 33.2 mg, 0.077

mmol) was purified by column chromatography using hexane:EtOAc mixtures (from 8:2 to 5:5).

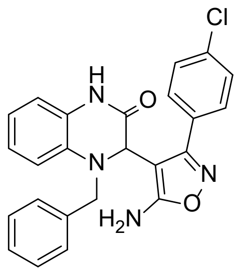

^{1}H NMR (300 MHz, DMSO-*d*$_6$) δ 10.54 (s, 1H), 7.37 – 7.10 (m, 9H), 6.78 – 6.65 (m, 3H), 6.64 – 6.55 (m, 2H), 6.46 – 6.39 (m, 1H), 5.13 (s, 1H), 4.44 (d, *J* = 16.4 Hz, 1H), 4.15 (d, *J* = 16.4 Hz, 1H) ppm.

^{13}C{^{1}H} NMR (75 MHz, DMSO-*d*$_6$) δ 169.4 (C), 165.4 (C), 161.8 (C), 137.5 (C), 133.8 (C), 129.7 (CH), 128.4 (C), 128.3 (CH), 127.9 (CH), 126.7 (CH), 126.6 (CH), 125.9 (C), 122.9 (CH), 118.0 (CH), 114.8 (CH), 112.5 (CH), 87.3 (C), 55.8 (CH), 50.6 (CH_2) ppm.

HRMS (ESI+) *m/z:* $[M + H]^+$ Calcd for $C_{24}H_{20}ClN_4O_2^+$ 431.1269; found 431.1279.

3-(5-Amino-3-(4-fluorophenyl)isoxazol-4-yl)-4-benzyl-3,4-dihydroquinoxalin-2(1*H*)-one (3aj)

Using 4-benzyl-3,4-dihydroquinoxalin-2-one (**1a**, 31.0 mg, 0.13 mmol, 1.3 equiv.) and 3-(4-fluorophenyl)isoxazol-5-amine (**2j**, 17.8 mg, 0.1 mmol, 1 equiv.) and **PC5**, according to GP-1, compound **3aj** (amorphous white solid, 72% yield, 29.9 mg, 0.072 mmol) was purified by column chromatography using hexane:EtOAc mixtures (from 8:2 to 5:5).

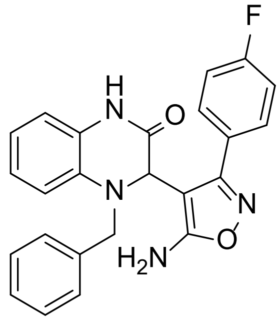

^{1}H NMR (300 MHz, DMSO-*d*$_6$) δ 10.54 (s, 1H), 7.41 – 7.28 (m, 2H), 7.23 – 7.09 (m, 5H), 7.07 – 6.97 (m, 2H), 6.76 – 6.49 (m, 5H), 6.43 (d, *J* = 8.0, 1H), 5.11 (s, 1H), 4.44 (d, *J* = 16.4 Hz, 1H), 4.14 (d, *J* = 16.4 Hz, 1H) ppm.

^{13}C{^{1}H} NMR (75 MHz, DMSO-*d*$_6$) δ 169.3 (C), 165.4 (C), 162.4 (C-F, d, *J* = 245.8 Hz), 162.0 (C), 137.5 (C), 133.8 (C), 130.2 (CH, d, *J* = 8.5 Hz), 128.3 (CH), 126.7 (CH), 126.6 (CH), 126.0 (CH, d, *J* = 3.1 Hz), 125.9 (CH), 122.9 (C), 118.1 (C), 115.0 (CH), 114.8 (CH), 114.7 (CH), 112.4 (CH), 87.4 (C), 55.8 (CH), 50.5 (CH_2) ppm.

^{19}F NMR (282 MHz, DMSO-*d*$_6$) δ **-**113.14.

HRMS (ESI+) *m/z:* $[M + H]^+$ Calcd for $C_{24}H_{20}FN_4O_2^+$ 415.1565; found 415.1557.

4-Benzyl-3-(5-(ethylamino)-3-methylisoxazol-4-yl)-3,4-dihydroquinoxalin-2(1*H*)-one (3ak)

Using 4-benzyl-1-methyl-3,4-dihydroquinoxalin-2(1*H*)-one (**1a**, 31 mg, 0.13 mmol, 1.3 equiv.) and *N*-ethyl-3-methylisoxazol-5-amine (**2k**, 12.6 mg, 0.1 mmol, 1 equiv.) and **PC5**, according to GP-1, compound **3ak** (orange oil, 36% yield, 13.2 mg, 0.036 mmol) was purified by column chromatography using hexane:EtOAc mixtures (from 8:2 to 5:5).

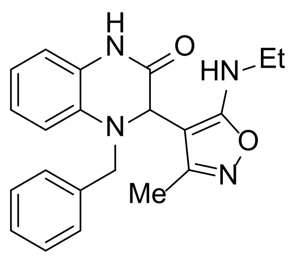

^{1}H NMR (300 MHz, $CDCl_3$) δ 9.15 (s, 1H), 7.40 – 7.26 (m, 5H), 7.02 (ddd, *J* = 8.1, 6.1, 3.0 Hz, 1H), 6.91 – 6.82 (m, 2H), 6.79 (d, *J* = 8.1 Hz, 1H), 4.79 – 4.62 (m, 2H), 4.03 (d, *J* = 14.8 Hz, 1H), 3.89 (t, *J* = 5.4 Hz, 1H), 3.29 – 3.03 (m, 2H), 1.86 (s, 3H), 0.91 (t, *J* = 7.2 Hz, 3H).

^{13}C{^{1}H} NMR (75 MHz, $CDCl_3$) δ 166.8 (C), 166.2 (C), 160.5 (C), 135.7 (C), 134.4 (C), 128.9 (CH), 127.9 (CH), 127.8 (CH), 124.9 (C), 124.9 (CH), 119.4 (CH), 115.9 (CH), 112.1 (CH), 86.9 (C), 55.2 (CH), 50.9 (CH_2), 37.6 (CH_2), 14.9 (CH_3), 10.8 (CH_3).

HRMS (ESI+) *m/z:* $[M + H]^+$ Calcd for $C_{21}H_{23}N_4O_2^+$ 363.1816; found 363.1825.

4-Benzyl-3-(5-(ethyl(methyl)amino)-3-methylisoxazol-4-yl)-3,4-dihydroquinoxalin-2(1*H*)-one (3al)

Using 4-benzyl-1-methyl-3,4-dihydroquinoxalin-2(1*H*)-one (**1a**, 31 mg, 0.13 mmol, 1.3 equiv.) and *N*-ethyl-*N*,3-dimethylisoxazol-5-amine (**2l**, 14 mg, 0.1 mmol, 1 equiv.) and **PC5**, according to GP-1, compound **3al** (yellow oil, 45% yield, 17.1 mg, 0.045 mmol) was purified by column chromatography using hexane:EtOAc mixtures (from 8:2 to 5:5)

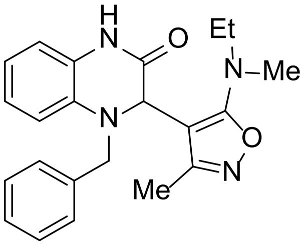

^{1}H NMR (300 MHz, $CDCl_3$) δ 9.09 (s, 1H), 7.26 – 7.20 (m, 4H), 7.14 – 7.07 (m, 2H), 6.86 (ddd, *J* = 8.1, 7.0, 2.0 Hz, 1H), 6.74 – 6.60 (m, 3H), 5.05 (s, 1H), 4.68 – 4.57 (m, 1H), 4.02 (d, *J* = 16.4 Hz, 1H), 3.23 (qd, *J* = 7.1, 2.2 Hz, 2H), 2.77 (s, 3H), 1.72 (s, 3H), 0.99 (t, *J* = 7.1 Hz, 3H).

^{13}C{^{1}H} NMR (75 MHz, $CDCl_3$) δ 168.9 (C), 166.9 (C), 161.3 (C), 136.3 (C), 134.5 (C), 128.7 (CH), 127.3 (CH), 127.3 (CH), 124.5 (CH), 124.4 (C), 118.5 (CH), 115.5 (CH), 112.2 (CH), 89.3 (C), 55.4 (CH), 49.6 (CH_2), 47.5 (CH_2), 37.0 (CH_3), 12.6 (CH_3), 11.3 (CH_3).

HRMS (ESI+) *m/z:* $[M + H]^+$ Calcd for $C_{22}H_{25}N_4O_2^+$ 377.1972; found 377.1988.

3-(5-amino-3-methylisothiazol-4-yl)-4-benzyl-3,4-dihydroquinoxalin-2(1*H*)-one (3am).

Using 4-benzyl-3,4-dihydroquinoxalin-2-one (**1a**, 31.0 mg, 0.13 mmol, 1.3 equiv.) and 3-methylisoxazol-5-amine (**2m**, 11.5 mg, 0.1 mmol, 1 equiv.), according to GP-1, compound 3al (yellow oil, 24% yield, 8.3 mg, 0.024 mmol) was purified by column chromatography using hexane:EtOAc mixtures (from 8:2 to 5:5).

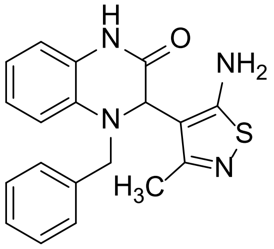

^{1}H NMR (300 MHz, CDCl$_3$) δ 8.95 (s, 1H), 7.34 – 7.27 (m, 3H), 7.18 (dd, J = 7.8, 1.9 Hz, 2H), 6.97 (ddd, J = 8.2, 5.8, 3.0 Hz, 1H), 6.83–6.73 (m, 3H), 5.13 (s, 1H), 4.75 (d, J = 16.0 Hz, 1H), 4.51 (s, 2H), 4.06 (d, J = 16.1 Hz, 1H), 1.99 (s, 3H) ppm.

^{13}C{^{1}H} NMR (75 MHz, CDCl$_3$) δ 169.4 (C), 166.2 (C), 166.0 (C), 135.8 (C), 134.3 (C), 128.9 (CH), 127.6 (CH), 127.5 (CH), 124.9 (CH), 124.2 (C), 118.9 (CH), 115.9 (CH), 112.9 (C), 112.0 (CH), 57.7 (CH), 50.3 (CH$_2$), 19.2 (CH$_3$) ppm.

HRMS (ESI+) *m/z:* [M + H]$^+$ Calcd for $C_{19}H_{19}N_4OS^+$ 351.1274; found 351.1267.

3-(5-Amino-3-phenylisoxazol-4-yl)-4-(4-(trifluoromethyl)benzyl)-3,4-dihydroquinoxalin-2(1*H*)-one (3bb)

Using 4-(4-(trifluoromethyl)benzyl)-3,4-dihydroquinoxalin-2(1*H*)-one (**1b**, 39.8 mg, 0.13 mmol, 1.3 equiv.) and 3-phenylisoxazol-5-amine (**1b**, 16 mg, 0.1 mmol, 1 equiv.) and **PC5**, according to GP-1, compound **3bb** (amorphous white solid, 96% yield, 44.7 mg, 0.096 mmol) was purified by column chromatography using hexane:EtOAc mixtures (from 8:2 to 5:5

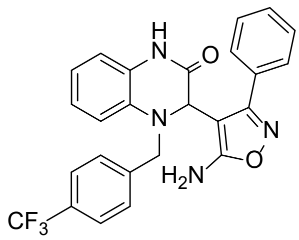

^{1}H NMR (300 MHz, DMSO-*d*$_6$) δ 10.58 (s, 1H), 7.52 (d, *J* = 8.1 Hz, 2H), 7.36 – 7.15 (m, 7H), 6.76 – 6.56 (m, 5H), 6.40 (d, *J* = 8.1, 1H), 5.11 (s, 1H), 4.56 (d, *J* = 17.0 Hz, 1H), 4.23 (d, *J* = 17.0 Hz, 1H) ppm.

^{13}C{^{1}H} NMR (75 MHz, DMSO-*d*$_6$) δ 169.3 (C), 165.4 (C), 162.8 (C), 142.6 (C), 133.7 (C), 129.5 (C), 128.9 (CH), 128.0 (CH), 127.9 (CH), 127.4 (CH), 126.1 (C), 125.6 (C, q, $J_{C\text{-}F}$ = 4.2 Hz), 123.0 (CH), 118.5 (CH), 115.0 (CH), 112.4 (CH), 86.8 (C), 79.2 (C), 55.8 (CH), 50.1 (CH$_2$) ppm.

^{19}F NMR (282 MHz, DMSO-*d*$_6$) δ -63.10 (s).

HRMS (ESI+) *m/z:* [M + H]$^+$ Calcd for $C_{25}H_{20}F_3N_4O_2^+$ 465.1533; found 465.1549.

3-(5-Amino-3-phenylisoxazol-4-yl)-4-(4-methoxybenzyl)-3,4-dihydroquinoxalin-2(1*H*)-one (3cb)

Using 4-(4-methoxybenzyl)-3,4-dihydroquinoxalin-2(1*H*)-one (**1c**, 34.9 mg, 0.13 mmol, 1.3 equiv.) and 3-phenylisoxazol-5-amine (**2b**, 16 mg, 0.1 mmol, 1 equiv.) and **PC5**, according to GP-1, compound **3cb** (amorphous orange solid, 66% yield, 28.2 mg, 0.066 mmol) was purified by column chromatography using hexane:EtOAc mixtures (from 8:2 to 5:5).

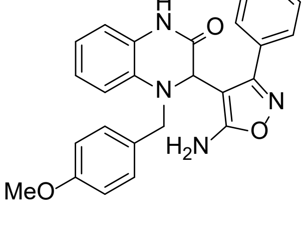

^{1}H NMR (300 MHz, CDCl$_3$) δ 8.52 (s, 1H), 7.64 – 7.57 (m, 2H), 7.41 – 7.27 (m, 3H), 7.00 – 6.88 (m, 3H), 6.82 – 6.75 (m, 2H), 6.69 (d, *J* = 8.1 Hz, 1H), 6.64 – 6.56 (m, 2H), 4.93 (s, 1H), 4.48 (d, *J* = 14.8 Hz, 1H), 4.21 (s, 2H), 3.83 (d, *J* = 14.7 Hz, 1H), 3.72 (s, 3H) ppm

^{13}C{^{1}H} NMR (75 MHz, CDCl$_3$) δ 167.0 (C), 166.2 (C), 164.6 (C), 159.0 (C), 134.4 (C), 129.5 (CH), 129.1 (C), 129.0 (CH), 128.6 (CH), 127.3 (C), 125.1 (CH), 124.7 (C), 119.4 (CH), 115.9 (CH), 114.1 (CH), 112.4 (CH), 89.7 (C), 55.3 (CH$_3$), 54.2 (CH), 50.2 (CH$_2$) ppm

HRMS (ESI+) *m/z:* [M + H]$^+$ Calcd for $C_{25}H_{23}N_4O_3^+$ 427.1765; found 427.1755.

3-(5-Amino-3-phenylisoxazol-4-yl)-4-(thiophen-2-ylmethyl)-3,4-dihydroquinoxalin-2(*1H*)-one (3db)

Using 4-(thiophen-2-ylmethyl)-3,4-dihydroquinoxalin-2(1*H*)-one (**1d**, 31.8 mg, 0.13 mmol, 1.3 equiv.) and 3-phenylisoxazol-5-amine (**2b**, 16 mg, 0.1 mmol, 1 equiv.) and **PC5**, according to GP-1, compound **3db** (orange oil, 40% yield, 16.2 mg, 0.04 mmol) was purified by column chromatography using hexane:EtOAc mixtures (from 8:2 to 5:5)

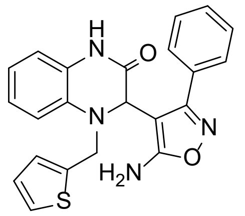

^{1}H NMR (300 MHz, DMSO-*d*$_6$) δ 10.51 (s, 1H), 7.50 – 7.21 (m, 8H), 6.86 – 6.72 (m, 3H), 6.70 – 6.61 (m, 3H), 5.04 (s, 1H), 4.65 (d, *J* = 16.4 Hz, 1H), 4.35 (d, *J* = 16.5 Hz, 1H) ppm.

^{13}C{^{1}H} NMR (75 MHz, DMSO-*d*$_6$) δ 169.3 (C), 165.2 (C), 162.9 (C), 140.4 (C), 133.5 (C), 129.8 (C), 128.1 (CH), 128.0 (CH), 126.5 (CH), 126.1 (C), 125.9 (CH), 125.0 (CH), 122.9 (CH), 118.6 (CH), 115.0 (CH), 112.6 (CH), 86.7 (C), 79.1 (C), 55.4 (CH), 46.1 (CH$_2$) ppm.

HRMS (ESI+) *m/z:* [M + H]$^+$ Calcd for $C_{22}H_{19}N_4O_2S^+$ 403.1223; found 403.1208.

Methyl 2-(2-(5-amino-3-phenylisoxazol-4-yl)-3-oxo-3,4-dihydroquinoxalin-1(2*H*)-yl) acetate (3eb)

Using methyl 2-(3-oxo-3,4-dihydroquinoxalin-1(2*H*)-yl)acetate (**1e**, 22 mg, 0.13 mmol, 1.3 equiv.) and 3-phenylisoxazol-5-amine (**2b**, 19 mg, 0.1 mmol, 1 equiv.) and **PC5**, according to GP-1, compound **3eb** (amorphous orange solid, 41% yield, 15.9 mg, 0.041 mmol) was purified by column chromatography using hexane:EtOAc mixtures (from 8:2 to 5:5).

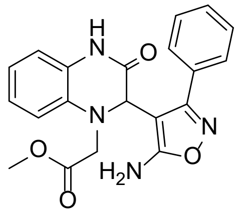

^{1}H NMR (300 MHz, DMSO-d_6) δ 10.45 (s, 1H), 7.55 – 7.47 (m, 2H), 7.37 – 7.23 (m, 3H), 6.83 (ddd, *J* = 8.0, 5.7, 3.3 Hz, 1H), 6.75 (s, 2H), 6.72 – 6.64 (m, 2H), 6.55 (d, *J* = 8.0 Hz, 1H), 5.09 (s, 1H), 4.13 (d, *J* = 18.2 Hz, 1H), 3.82 (d, *J* = 18.2 Hz, 1H), 3.51 (s, 3H) ppm.

^{13}C {^{1}H} NMR (75 MHz, DMSO) δ 169.6 (C), 169.2 (C), 164.8 (C), 162.6 (C), 133.7 (C), 129.5 (C), 128.6 (CH), 127.7 (CH), 127.6 (CH), 125.9 (C), 122.5 (CH), 118.4 (CH), 114.5 (CH), 111.7 (CH), 84.8 (C), 55.0 (CH_3), 51.2 (CH), 47.2 (CH_2) ppm.

HRMS (ESI+) *m/z:* $[M + H]^+$ Calcd for $C_{20}H_{19}N_4O_4^+$ 379.1401; found 379.1419.

3-(5-Amino-3-phenylisoxazol-4-yl)-4-benzyl-1-methyl-3,4-dihydroquinoxalin-2(1*H*)-one (3fb)

Using 4-benzyl-1-methyl-3,4-dihydroquinoxalin-2(1*H*)-one (**1f**, 32.8 mg, 0.13 mmol, 1.3 equiv.) and 3-phenylisoxazol-5-amine (**2b**, 16 mg, 0.1 mmol, 1 equiv.) and **PC5**, according to GP-1, compound **3fb** (brown oil, 32% yield, 13.3 mg, 0.032 mmol) was purified by column chromatography using hexane:EtOAc mixtures (from 8:2 to 5:5).

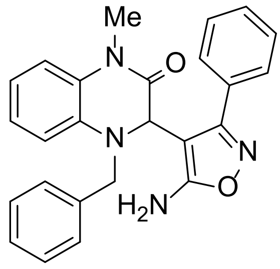

^{1}H NMR (300 MHz, $CDCl_3$) δ 7.60 – 7.52 (m, 2H), 7.38 – 7.27 (m, 3H), 7.15 – 7.09 (m, 3H), 7.08 – 7.01 (m, 2H), 7.01 – 6.85 (m, 3H), 6.65 (dd, *J* = 7.9, 1.5 Hz, 1H), 4.97 (s, 1H), 4.52 – 4.42 (m, 1H), 4.14 (s, 2H), 3.90 (d, *J* = 15.2 Hz, 1H), 3.45 (s, 3H).

^{13}C{^{1}H} NMR (75 MHz, $CDCl_3$) δ 166.7 (C), 165.2 (C), 164.6 (C), 135.6 (C), 129.3 (CH), 128.9 (C), 128.8 (CH), 128.6 (CH), 128.4 (CH), 127.4 (CH), 127.4 (CH), 124.6 (CH), 119.4 (CH), 114.8 (CH), 112.7 (CH), 89.8 (C), 55.1 (CH), 51.2 (CH_2), 29.3 (CH_3).

HRMS (ESI+) *m/z:* $[M + H]^+$ Calcd for $C_{25}H_{23}N_4O_2^+$ 411.1816; found 411.1819.

3-(5-Amino-3-phenylisoxazol-4-yl)-1,4-dibenzyl-3,4-dihydroquinoxalin-2(1*H*)-one (3gb)

Using 1,4-dibenzyl-3,4-dihydroquinoxalin-2(1*H*)-one (**1g**, 42.7 mg, 0.13 mmol, 1.3 equiv.) and 3-phenylisoxazol-5-amine (**2b**, 16 mg, 0.1 mmol, 1 equiv.) and **PC5**, according to GP-1, compound **3gb** (brown oil, 55% yield, 26.9 mg, 0.055 mmol) was purified by column chromatography using hexane:EtOAc mixtures (from 8:2 to 5:5).

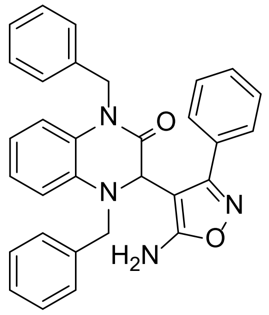

^{1}H NMR (300 MHz, $CDCl_3$) δ 7.68 (dd, *J* = 7.9, 1.7 Hz, 2H), 7.40 – 7.27 (m, 8H), 7.14-7.11 (m, 3H), 7.06 – 7.03 (m, 2H), 6.97 – 6.90 (m, 2H), 6.76 (td, *J* = 7.7, 1.3 Hz, 1H), 6.68 (d, *J* = 8.0, 1H), 5.57 (d, *J* = 16.1 Hz, 1H), 5.08 (s, 1H), 4.98 (d, *J* = 16.1 Hz, 1H), 4.49 (d, *J* = 15.0 Hz, 1H), 4.10 (s, 2H), 3.92 (d, *J* = 15.0 Hz, 1H).

^{13}C{^{1}H} NMR (75 MHz, $CDCl_3$) δ 166.8 (C), 165.4 (C), 164.9 (C), 136.2 (C), 135.8 (C), 135.5 (C), 129.4 (CH), 129.0 (CH), 128.9 (CH), 128.8 (C), 128.6 (C), 128.5 (CH), 128.5 (CH), 127.7 (C), 127.4 (CH), 127.4 (CH), 127.4 (CH), 126.6 (C), 126.3 (CH), 124.8 (CH), 119.5 (CH), 115.7 (CH), 112.8 (CH), 89.7 (C), 55.1 (CH), 51.3 (CH_2), 46.3 (CH_2).

HRMS (ESI+) *m/z:* $[M + H]^+$ Calcd for $C_{31}H_{27}N_4O_2^+$ 487.2129; found 487.2134.

3-(5-amino-3-phenylisoxazol-4-yl)-4-benzyl-7-methyl-3,4-dihydroquinoxalin-2(1*H*)-one (3hb)

Using 4-benzyl-7-methyl-3,4-dihydroquinoxalin-2(1*H*)-one (**1h**, 31.3 mg, 0.13 mmol, 1.3 equiv.) and 3-phenylisoxazol-5-amine (**2b**, 16 mg, 0.1 mmol, 1 equiv.) and **PC5**, according to GP-1, compound **3hb** (orange oil, 52% yield, 21.5 mg, 0.052 mmol) was purified by column chromatography using hexane:EtOAc mixtures (from 8:2 to 5:5)

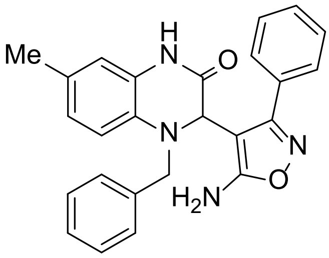

^{1}H NMR (300 MHz, DMSO-d_6) δ 9.54 (s, 1H), 7.61 (dd, *J* = 8.0, 1.6 Hz, 2H), 7.37 – 7.27 (m, 4H), 7.12 (dt, *J* = 5.3, 1.9 Hz, 2H), 7.06 – 7.00 (m, 2H), 6.73 (ddd, *J* = 8.2, 2.0, 0.8 Hz, 1H), 6.66 – 6.62 (m, 1H), 6.54 (d, *J* = 8.2 Hz, 1H), 4.93 (s, 1H), 4.48 (d, *J* = 15.1 Hz, 1H), 4.32 (s, 2H), 3.90 (d, *J* = 15.3 Hz, 1H), 2.22 (s, 3H).

^{13}C{^{1}H} NMR (75 MHz, DMSO) δ 166.9 (C), 166.5 (C), 164. (C), 135.8 (C), 131.8 (C), 129.4 (CH), 129.0 (C), 128.9 (C), 128.8 C(H), 128.6 (CH), 128.5 (CH), 128.4 (CH), 127.9 (C), 127.4 (CH), 127.3 (CH), 125.3 (CH), 124.7 (C), 89.5 (CH), 54.8 (CH), 50.9 (CH_2), 20.3 (CH_3).

HRMS (ESI+) *m/z:* $[M + H]^+$ Calcd for $C_{25}H_{23}N_4O_2^+$ 411.1816; found 411.1810.

3-(5-amino-3-phenylisoxazol-4-yl)-4-benzyl-7-methoxy-3,4-dihydroquinoxalin-2(1*H*)-one (3ib)

Using 4-benzyl-7-methoxy-3,4-dihydroquinoxalin-2(1*H*)-one (**1i**, 34.9 mg, 0.13 mmol, 1.3 equiv.) and 3-phenylisoxazol-5-amine (**2b**, 16 mg, 0.1 mmol, 1 equiv.) and **PC5**, according to GP-1, compound **3ib** (orange oil, 78% yield, 33.5 mg, 0.078 mmol) was purified by column chromatography using hexane:EtOAc mixtures (from 8:2 to 5:5)

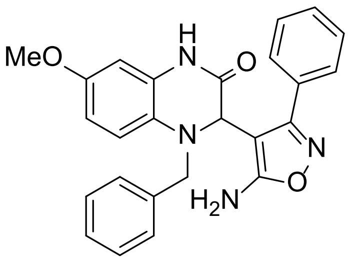

^{1}H NMR (300 MHz, DMSO-*d*$_6$) δ 8.31 (s, 1H), 7.39 (dd, J = 8.2, 1.5 Hz, 2H), 7.36–7.29 (m, 1H), 7.26 (d, J = 7.5 Hz, 2H), 7.23 – 7.07 (m, 5H), 6.71–6.48 (m, 3H), 6.18 (dd, J = 8.5, 2.5 Hz, 1H), 6.01 (d, J = 2.5 Hz, 1H), 5.08 (s, 1H), 4.43 (d, J = 16.3 Hz, 1H), 4.09 (d, J = 16.3 Hz, 1H), 3.53 (s, 3H).

^{13}C{^{1}H} NMR (75 MHz, DMSO) δ 169.2 (C), 164.6 (C), 162.9 (C), 155.6 (C), 137.6 (C), 134.9 (C), 129.7 (C), 128.9 (CH), 128.3 (CH), 128.1 (CH), 128.0 (CH), 126.8 (CH), 126.7 (CH), 119.7 (C), 115.2 (CH), 102.1 (CH), 99.8 (CH), 87.5 (C), 55.8 (CH), 54.9 (CH_3), 50.8 (CH_2).

HRMS (ESI+) *m/z:* $[M + H]^+$ Calcd for $C_{25}H_{23}N_4O_3^+$ 427.1765; found 427.1759.

3-(5-Amino-3-phenylisoxazol-4-yl)-4-benzyl-7-bromo-3,4-dihydroquinoxalin-2(1*H*)-one (3jb)

Using 4-benzyl-7-bromo-3,4-dihydroquinoxalin-2(1*H*)-one (**1j**, 41.2 mg, 0.13 mmol, 1.3 equiv.) and 3-phenylisoxazol-5-amine (**2b**, 19 mg, 0.1 mmol, 1 equiv.) and **PC5**, according to GP-1, compound **3jb** (orange oil, 70% yield, 33.3 mg, 0.07 mmol) was purified by column chromatography using hexane:EtOAc mixtures (from 8:2 to 5:5)

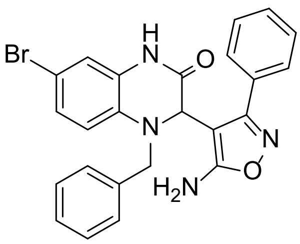

^{1}H NMR (300 MHz, DMSO-*d*$_6$) δ 10.55 (s, 1H), 7.36 – 7.25 (m, 1H), 7.25 – 7.11 (m, 9H), 6.87 (s, 2H), 6.77 (dd, *J* = 8.6, 2.3 Hz, 1H), 6.54 (d, *J* = 2.4 Hz, 1H), 6.25 (d, *J* = 8.7 Hz, 1H), 5.22 (s, 1H), 4.38 (d, *J* = 16.6 Hz, 1H), 4.20 (d, *J* = 16.6 Hz, 1H).

^{13}C{^{1}H} NMR (75 MHz, DMSO) δ 169.4 (C), 165.5 (C), 162.7 (C), 137.3 (C), 132.8 (C), 129.5 (C), 128.6 (CH), 128.4 (CH), 128.0 (CH), 127.8 (CH), 127.5 (C), 126.8 (CH), 126.6 (CH), 124.8 (CH), 116.9 (CH), 113.9 (CH), 108.8 (C), 87.4 (C), 56.0 (CH), 50.2 (CH_2).

HRMS (ESI+) *m/z:* $[M + H]^+$ Calcd for $C_{24}H_{20}BrN_4O_2^+$ 475.0764; found 475.0775.

3-(5-amino-3-phenylisoxazol-4-yl)-4-benzyl-6-fluoro-3,4-dihydroquinoxalin-2(1*H*)-one (3kb)

Using 4-benzyl-6-fluoro-3,4-dihydroquinoxalin-2(1*H*)-one (**1k**, 33.5 mg, 0.13 mmol, 1.3 equiv.) and 3-phenylisoxazol-5-amine (**2b**, 16 mg, 0.1 mmol, 1 equiv.) and **PC5**, according to GP-1, compound **3kb** (orange oil, 45% yield, 18.8 mg, 0.045 mmol) was purified by column chromatography using hexane:EtOAc mixtures (from 8:2 to 5:5)

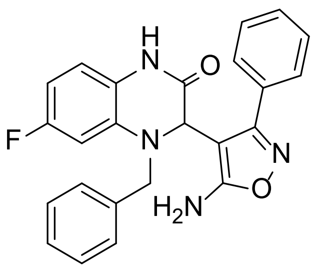

^{1}H NMR (300 MHz, DMSO-*d*$_6$) δ 10.52 (s, 1H), 7.30 – 7.11 (m, 10H), 6.77 (s, 2H), 6.50 (dd, J = 8.5, 5.9 Hz, 1H), 6.30 (td, J = 8.5, 2.6 Hz, 1H), 6.16 (dd, J = 11.7, 2.6 Hz, 1H), 5.20 (s, 1H), 4.43 (d, J = 16.6 Hz, 1H), 4.15 (d, J = 16.6 Hz, 1H) ppm.

^{13}C{^{1}H} NMR (75 MHz, DMSO-*d*$_6$) δ 167.0 (C), 164.3 (C), 162.4 (C), 158.2 (C-F, d, J = 235.5 Hz), 136.8 (C), 134.6 (C, d, J = 10.9 Hz), 129.2 (C), 128.6 (CH), 128.1 (CH), 127.8 (CH) , 127.6 (CH) , 126.5 (CH), 126.4 (CH), 121.7 (C, d, J = 1.9 Hz), 114.9 (CH, d, J = 10.4 Hz), 102.85 (CH, d, J = 22.6 Hz), 99.0 (CH, d, J = 28.2 Hz), 87.4 (C) , 55.3 (CH), 50.3 (CH$_2$) ppm.

^{19}F{^{1}H} NMR (282 MHz, DMSO-*d*$_6$) δ **-**120.14.

HRMS (ESI+) *m/z:* [M + H]$^+$ Calcd for $C_{24}H_{20}FN_4O_2^+$ 415.1565; found 415.1572.

3-(5-amino-3-phenylisoxazol-4-yl)-4-benzyl-5-methyl-3,4-dihydroquinoxalin-2(1*H*)-one (3lb)

Using 4-benzyl-5-methyl-3,4-dihydroquinoxalin-2(1*H*)-one (**1l**, 31.3 mg, 0.13 mmol, 1.3 equiv.) and 3-phenylisoxazol-5-amine (**2b**, 16 mg, 0.1 mmol, 1 equiv.) and **PC5**, according to GP-1, compound **3hb** (orange oil, 58% yield, 23.8 mg, 0.058 mmol) was purified by column chromatography using hexane:EtOAc mixtures (from 8:2 to 5:5)

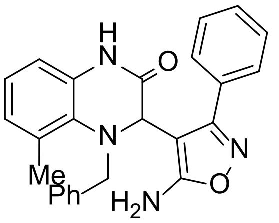

^{1}H NMR (300 MHz, DMSO-*d*$_6$) 10.33 (s, 1H), 7.49–7.30 (m, 5H), 7.24–7.12 (m, 3H), 6.99-6.96 (m, 2H), 6.92 (t, J = 7.7 Hz, 1H), 6.77 (d, J = 7.1 Hz, 1H), 6.60 (d, J = 7.8 Hz, 1H), 5.91 (s, 2H), 4.64 (s, 1H), 3.95 (d, J = 14.0 Hz, 1H), 3.84 (d, J = 14.0 Hz, 1H), 1.86 (s, 3H).

^{13}C{^{1}H} NMR (75 MHz, DMSO-d$_6$) δ 166.9 (C), 166.7 (C), 163.4 (C), 136.7 (C), 133.4 (C), 133.2 (C), 131.7 (C), 129.9 (C), 129.0 (CH), 128.2 (CH), 128.1 (CH), 128.0 (CH), 127.4 (CH), 125.1 (CH), 124.8 (CH), 113.6 (CH), 113.5 (CH), 86.0 (C), 57.1 (CH), 56.2 (CH$_2$), 16.8 (CH$_3$).

HRMS (ESI+) *m/z:* [M + H]$^+$ Calcd for $C_{25}H_{23}N_4O_2^+$ 411.1816; found 411.1808.

3-(5-Amino-3-phenylisoxazol-4-yl)-4-benzyl-5-chloro-3,4-dihydroquinoxalin-2(1*H*)-one (3mb)

Using 4-benzyl-5-chloro-3,4-dihydroquinoxalin-2(1*H*)-one (**1m**, 35.5 mg, 0.13 mmol, 1.3 equiv.) and 3-phenylisoxazol-5-amine (**2b**, 16 mg, 0.1 mmol, 1 equiv.) and **PC5**, according to GP-1, compound **3mb** (yellow oil, 37% yield, 15.9 mg, 0.037 mmol) was purified by column chromatography using hexane:EtOAc mixtures (from 8:2 to 5:5)

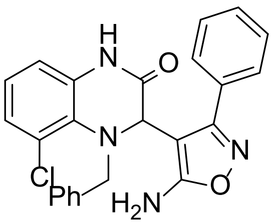

^{1}H NMR (300 MHz, CDCl$_3$) δ 8.72 (s, 1H), 7.60–7.45 (m, 2H), 7.42–7.21 (m, 10H), 7.13 (dd, J = 8.1, 1.4 Hz, 1H), 7.03 (t, J = 8.0 Hz, 1H), 6.53 (dd, J = 7.8, 1.4 Hz, 1H), 4.61 (s, 1H), 4.27 (d, J = 13.8 Hz, 1H), 4.02 (d, J = 13.8 Hz, 1H).

^{13}C{^{1}H} NMR (75 MHz, CDCl$_3$) δ 167.3 (C), 166.6 (C), 163.8 (C), 136.1 (C), 133.8 (C), 131.0 (C), 130.0 (C), 129.5 (CH), 129.2 (CH), 129.1 (CH), 129.0 (C), 128.6 (CH), 128.2 (CH), 127.8 (CH), 126.2 (CH), 125.5 (CH), 114.9 (CH), 86.4 (C), 56.4 (CH), 55.4 (CH$_2$).

HRMS (ESI+) *m/z:* [M + H]$^+$ Calcd for $C_{24}H_{20}ClN_4O_2^+$ 431.1269; found 431.1275.

3-(5-Amino-3-phenylisoxazol-4-yl)-4-benzyl-6,7-dichloro-3,4-dihydroquinoxalin-2(1*H*)-one (3nb)

Using 4-benzyl-6,7-dichloro-3,4-dihydroquinoxalin-2(1*H*)-one (**1n**, 39.9 mg, 0.13 mmol, 1.3 equiv.) and 3-phenylisoxazol-5-amine (**2b**, 16 mg, 0.1 mmol, 1 equiv.) and **PC5**, according to GP-1, compound **3nb** (amorphous yellow solid, 24% yield, 11.3 mg, 0.024 mmol) was purified by column chromatography using hexane:EtOAc mixtures (from 8:2 to 5:5).

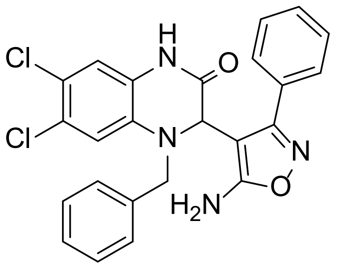

^{1}H NMR (300 MHz, DMSO-*d$_6$*) δ 10.64 (s, 1H), 7.31 – 7.24 (m, 2H), 7.23 (s, 1H), 7.22 – 7.17 (m, 3H), 7.15 – 7.16 (m, 1H), 7.14 – 7.09 (m, 3H), 6.98 (s, 2H), 6.47 (s, 1H), 6.38 (s, 1H), 5.29 (s, 1H), 4.42 (d, J = 16.7 Hz, 1H), 4.21 (d, J = 16.7 Hz, 1H).

^{13}C{^{1}H} NMR (75 MHz, DMSO) δ 169.8 (C), 165.5 (C), 163.1 (C), 137.3 (C), 133.9 (C), 129.9 (C), 129.0 (CH), 128.9 (CH), 128.6 (CH), 128.1 (CH), 127.5 (CH), 127.2 (CH), 126.3 (CH), 124.4 (C), 118.9 (C), 115.8 (CH), 113.1 (CH), 88.3 (C), 56.2 (CH), 51.1 (CH$_2$).

HRMS (ESI+) *m/z:* [M + H]$^+$ Calcd for $C_{24}H_{19}Cl_2N_4O_2^+$ 465.0880; found 465.0868.

3-(5-Amino-3-phenylisoxazol-4-yl)-4-benzyl-6,7-dimethyl-3,4-dihydroquinoxalin-2(1*H*)-one (3ob)

Using 4-benzyl-6,7-dimethyl-3,4-dihydroquinoxalin-2(1*H*)-one (**1o**, 34.6 mg, 0.13 mmol, 1.3 equiv.) and 3-phenylisoxazol-5-amine (**2b**, 16 mg, 0.1 mmol, 1 equiv.) and **PC5**, according to GP-1, compound **3ob** (amorphous red solid, 46% yield, 19.5 mg, 0.046 mmol) was purified by column chromatography using hexane:EtOAc mixtures (from 8:2 to 5:5)

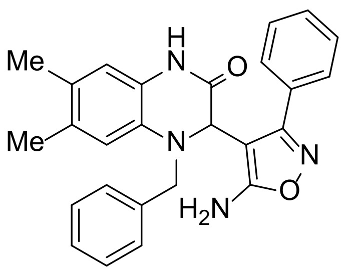

^{1}H NMR (300 MHz, $CDCl_3$) δ 9.30 (s, 1H), 7.65 – 7.58 (m, 2H), 7.40 – 7.27 (m, 3H), 7.14 – 7.07 (m, 3H), 7.06 – 6.97 (m, 2H), 6.60 (s, 1H), 6.45 (s, 1H), 4.89 (s, 1H), 4.50 (d, *J* = 15.2 Hz, 1H), 4.29 (s, 2H), 3.87 (d, *J* = 15.2 Hz, 1H), 2.13 (d, *J* = 1.6 Hz, 3H), 2.12 (s, 3H).

$^{13}C\{^1H\}$ NMR (75 MHz, $CDCl_3$) δ 166.9 (C), 166.2 (C), 164.5 (C), 135.9 (C), 132.9 (C), 132.01 (C), 129.3 (CH), 129.01 (C), 128.9 (CH), 128.6 (CH), 128.5 (CH), 128.4 (CH), 127.51 (CH), 127.4 (C), 127.3 (CH), 122.4 (C), 117.1 (CH), 113.7 (CH), 89.6 (C), 54.5 (CH), 50.7 (CH_2), 19.7 (CH_3), 18.7 (CH_3).

HRMS (ESI+) *m/z:* $[M + H]^+$ Calcd for $C_{26}H_{25}N_4O_2^+$ 425.1972; found 425.1977.

4-(1-benzyl-3-oxo-3,4-dihydroquinoxalin-2(*1H*)-ylidene)-3-methylisoxazol-5(*4H*)-one (4)

Using 4-benzyl-1-methyl-3,4-dihydroquinoxalin-2(1*H*)-one (**1a**, 310 mg, 1.3 mmol, 1.3 equiv.) and 3-phenylisoxazol-5-amine (**2a**, 160 mg, 1 mmol, 1 equiv.), according to SP, compound **4** (amorphous orange solid, 35% yield, 11.8 mg, 0.035 mmol) was purified by column chromatography using hexane:EtOAc mixtures (from 8:2 to 5:5).

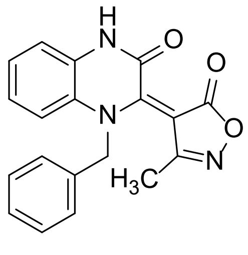

^{1}H NMR (300 MHz, $CDCl_3$) δ 9.20 (s, 1H), 7.43 (dd, *J* = 7.9, 1.5 Hz, 1H), 7.40 – 7.26 (m, 5H), 7.17 (ddd, *J* = 7.9, 7.4, 1.5 Hz, 1H), 6.89 – 6.73 (m, 2H), 4.37 (s, 2H), 2.43 (s, 3H).

$^{13}C\{^1H\}$ NMR (75 MHz, $CDCl_3$) δ 161.9 (C), 141.9 (C), 138.6 (C), 128.7 (CH), 128.3 (CH), 127.4 (CH), 127.4 (CH), 125.0 (CH), 121.8 (C), 118.3 (CH), 113.4 (CH), 95.8 (C), 48.3 (CH_2), 13.4 (CH_3).

HRMS (ESI+) *m/z:* $[M + H]^+$ Calcd for $C_{19}H_{16}N_3O_3^+$ 334.1186; found 334.1197.

Mechanistic Investigations

Luminescence Quenching Experiments

Freshly distilled DCM over CaH_2 was degassed by sonication and N_2 bubbling simultaneously. The measurement solutions were prepared from stock solutions of **1a** (32 mM), **2a** (32 mM), and **PC4** (0.4 mM) in anhydrous and degassed DCM. Table S1 shows the concentration of each analyte in the measurement solutions for all the experiences.

*Table S1: Concentration of **1a**, **2a** and **PC4** in each solution.*

Solution	[1a] (mM)	[2a] (mM)	[PC4] (mM)
Blank	-	-	0,02
A1	4,8	-	0,02
A2	9,6	-	0,02
A3	14,4	-	0,02
B1	-	4,8	0,02
B2	-	9,6	0,02
B3	-	14,4	0,02

All the emission spectra were obtained using a Jasco FP-750 Spectrofluorometer selecting 450 nm as the excitation wavelength.

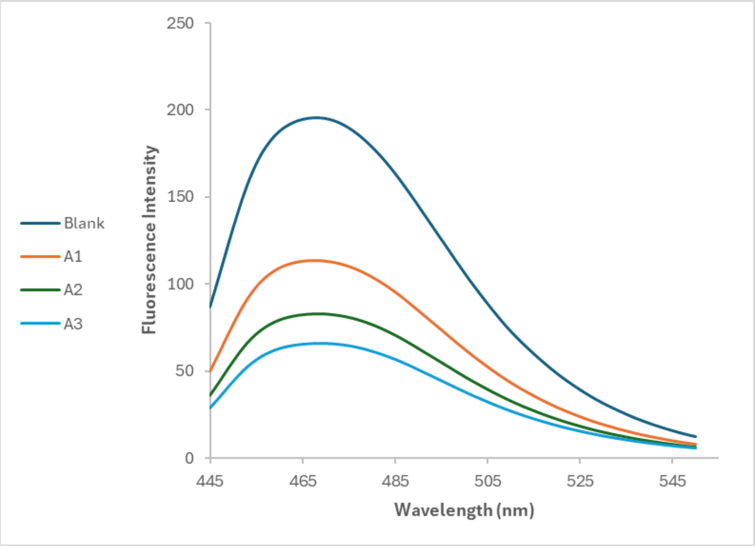

*Figure S2: Emission spectra of solutions containing 0.02 mM of **PC5** and increasing amounts of **1a**.*

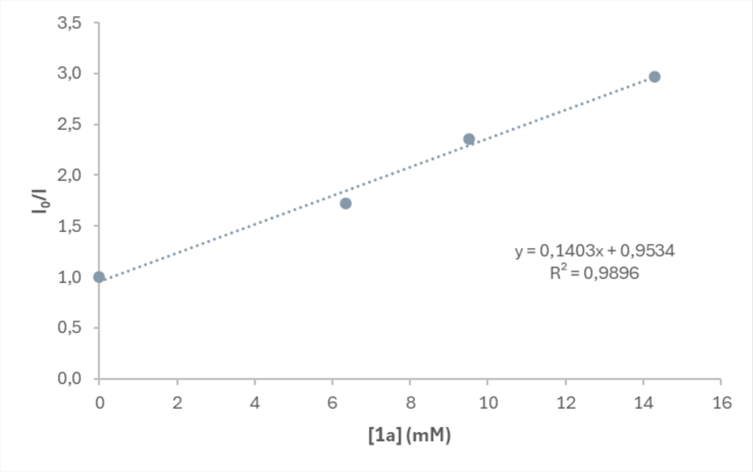

*Figure S3: Stern-Volmer plot of I_0/I against [**1a**] and fitting a linear regression model.*

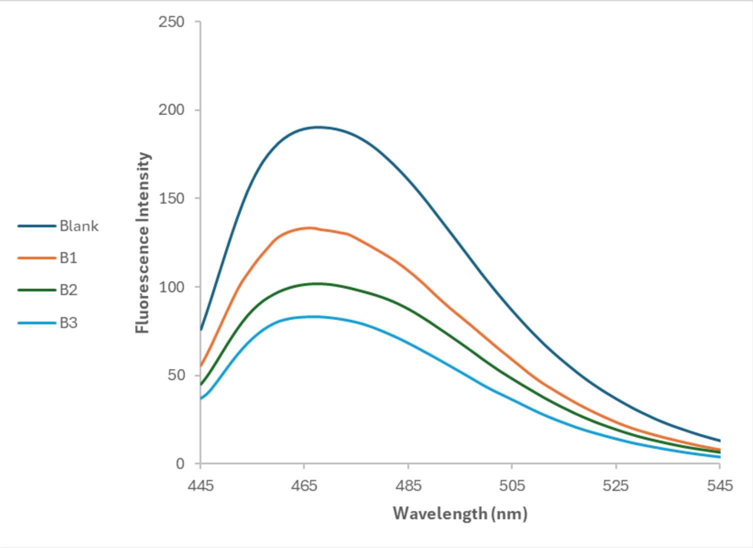

*Figure S4: Emission spectra of solutions containing 0.02 mM of **PC5** and increasing amounts of **2a**.*

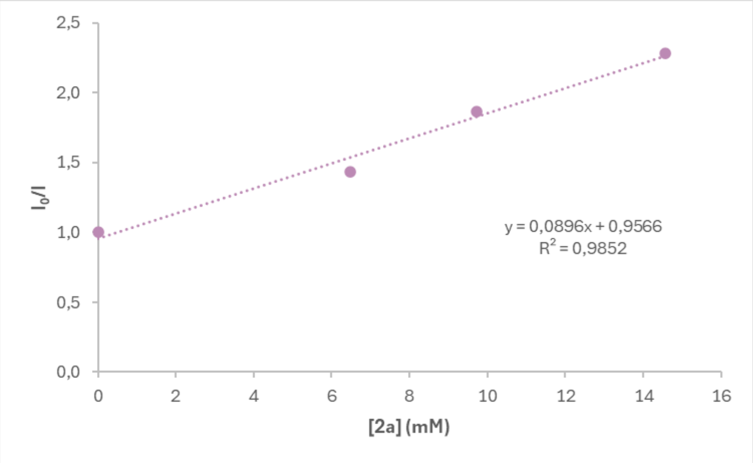

*Figure S5: Stern-Volmer plot of I_0/I against [**2a**] and fitting a linear regression model.*

Detection of H_2O_2 by iodide oxidation

The reaction between **1a** and **2a** was performed using either **PC4** or **PC5** under normal conditions. After 9 hours of irradiation, the organic phase was extracted with water (4x15 mL). The combined aqueous phases were passed through a pad of activated charcoal. Then, H2SO4 (1N, 6 mL) and KI (0.5 M, 10 mL) were added and homogenized. Finally, a starch solution (1%, 3 mL) was also added under stirring. After 10 seconds, a pale blue color appeared.

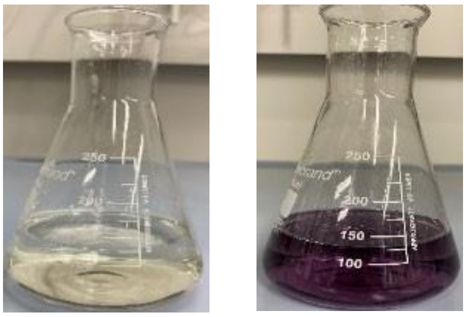

Figure S6: Reaction conducted with PC5.

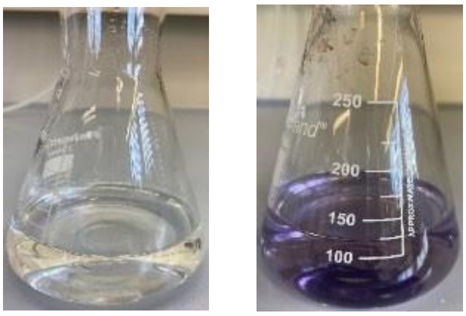

Figure S7 : Reaction conducted with PC4.

Unsuccessful examples

Unsuccessful Examples

No conversion to the desired product was observed; instead, oxidation products from dihydroquinoxalin-2-one were detected, and the unreacted 5-aminoisoxazole starting material was recovered.

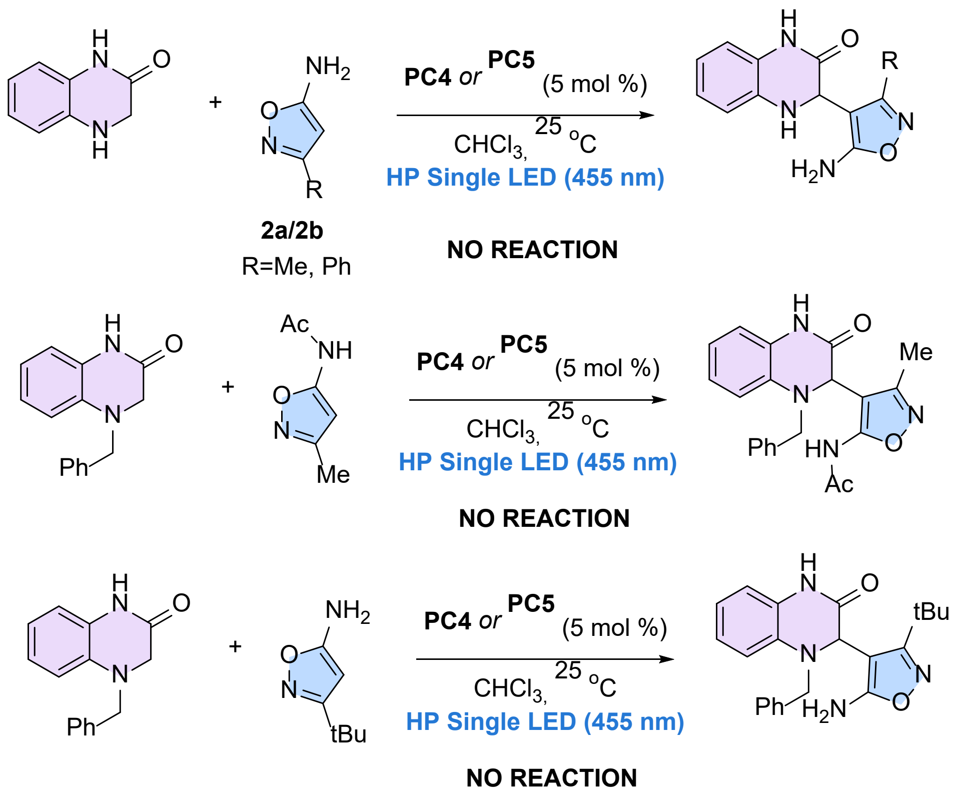

Scheme S1. Unsuccessful examples

References

[1] Rostoll-Berenguer, J.; Blay, G.; M. Carmen Muñoz; Pedro, J. R.; Vila, C. A Combination of Visible-Light Organophotoredox Catalysis and Asymmetric Organocatalysis for the Enantioselective Mannich Reaction of Dihydroquinoxalinones with Ketones. *Org. Lett.* **2019**, *21*, 6011–6015.

[2] Rostoll-Berenguer, J.; Blay, G.; Pedro, J. R.; Vila, C. Photocatalytic Giese Addition of 1,4-Dihydroquinoxalin-2-Ones to Electron-Poor Alkenes Using Visible Light. *Org. Lett.* **2020**, *22*, 8012–8017.

[3] Liu, H.; Yan, Y.; Li, M.; Zhang, X. An enantioselective aza-Friedel–Crafts reaction of 5-aminoisoxazoles with isatin-derived N-Boc ketimines. *Org. Biomol. Chem.* **2021**, *19*, 3820-3824.

[4] Luo, W.; Guo, H.; Qiu, X.; Min, M.; Zhang, L.; Zhu, H.; Zhou, J. Organocatalytic Atroposelective Construction of Pentatomic Heterobiaryl Diamines through Arylation of 5-Aminoisoxazoles with Azonaphthalenes. *Org. Lett.* **2024**, *26*, 2564-2568.

NMR Spectra

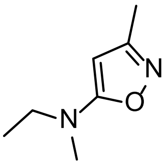

2l
^{1}H-NMR ($CDCl_3$, 300 MHz)
^{13}C{^{1}H} NMR ($CDCl_3$, 75 MHz)

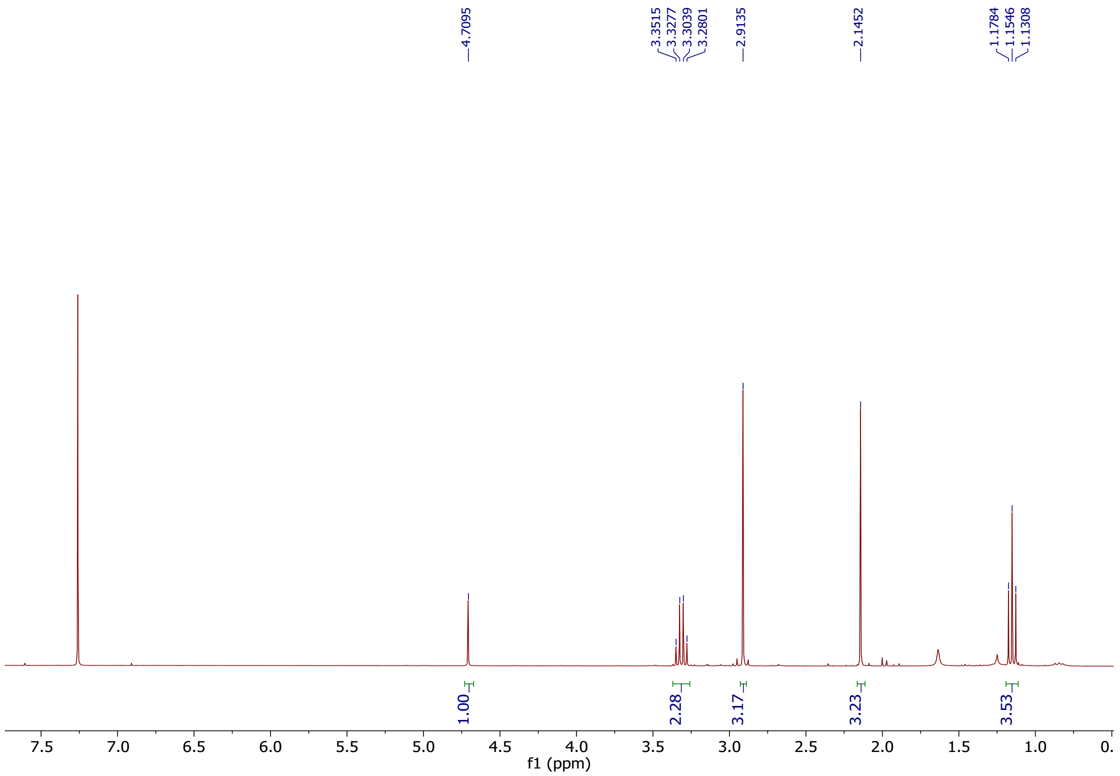

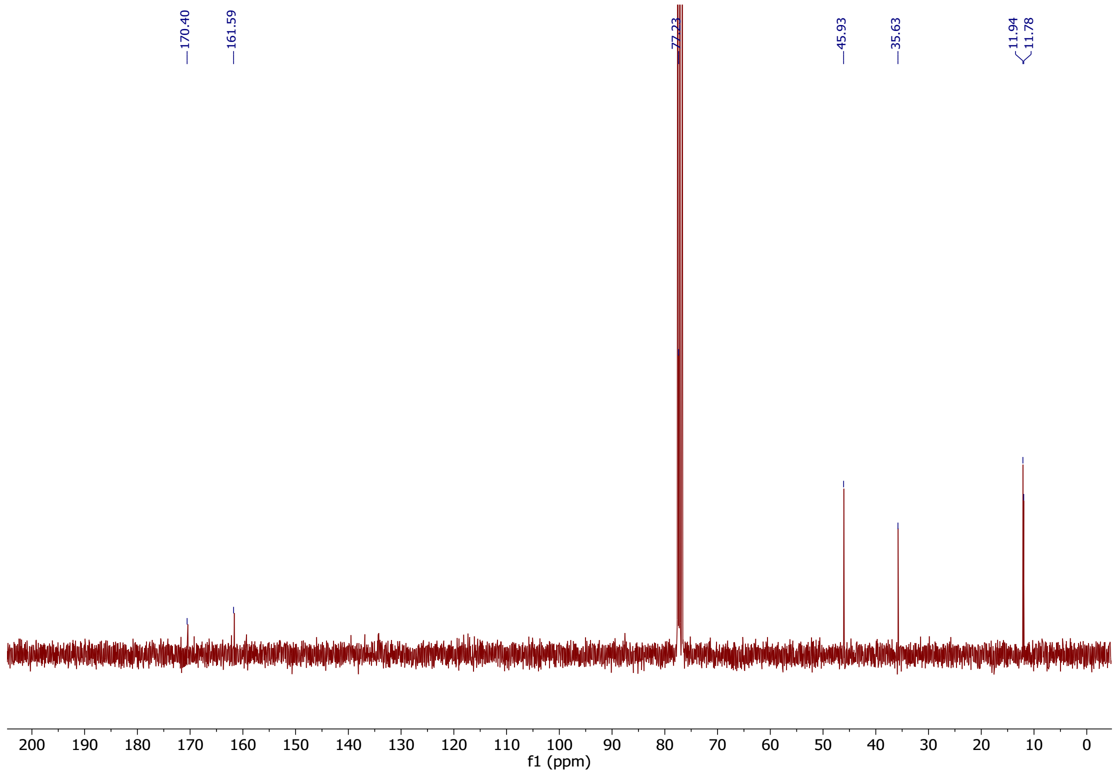

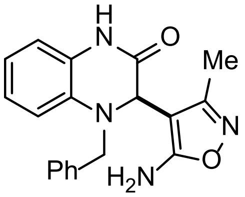

3aa

^{1}H-NMR ($CDCl_3$, 300 MHz)

$^{13}C\{^{1}H\}$ NMR ($CDCl_3$, 75 MHz)

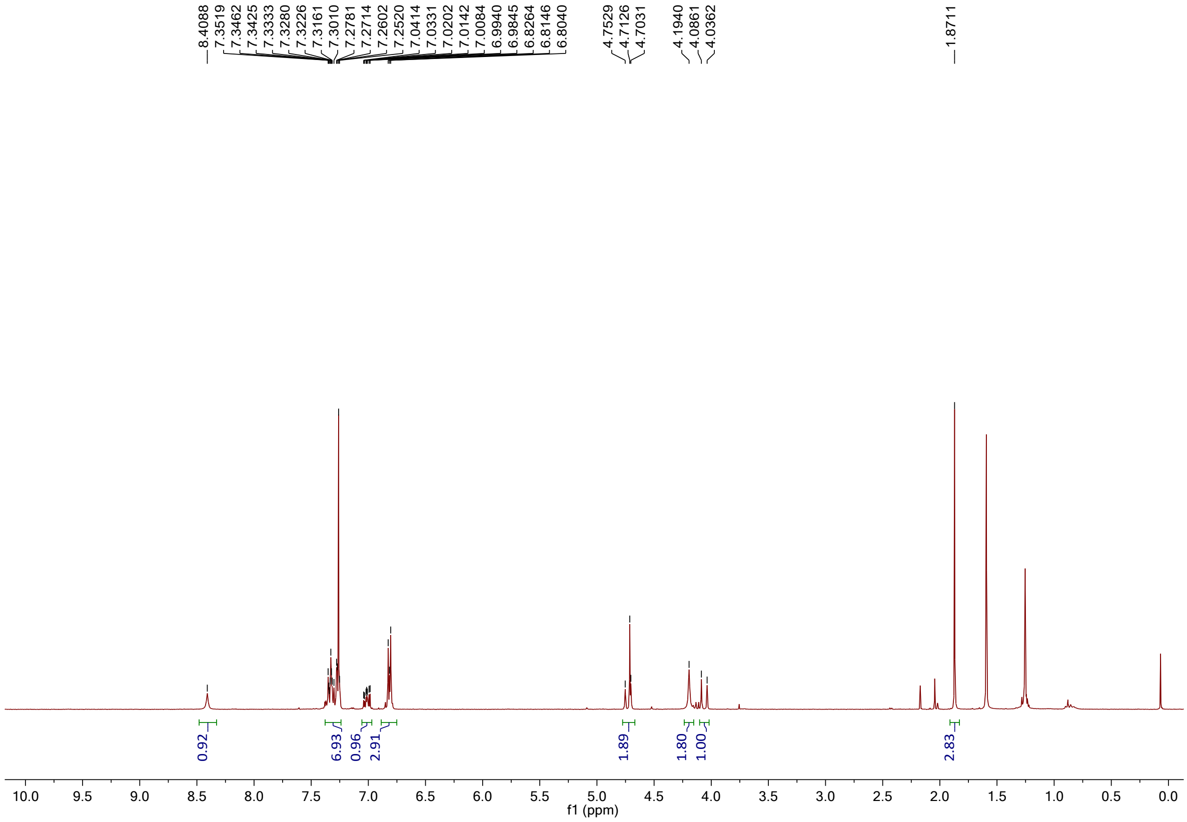

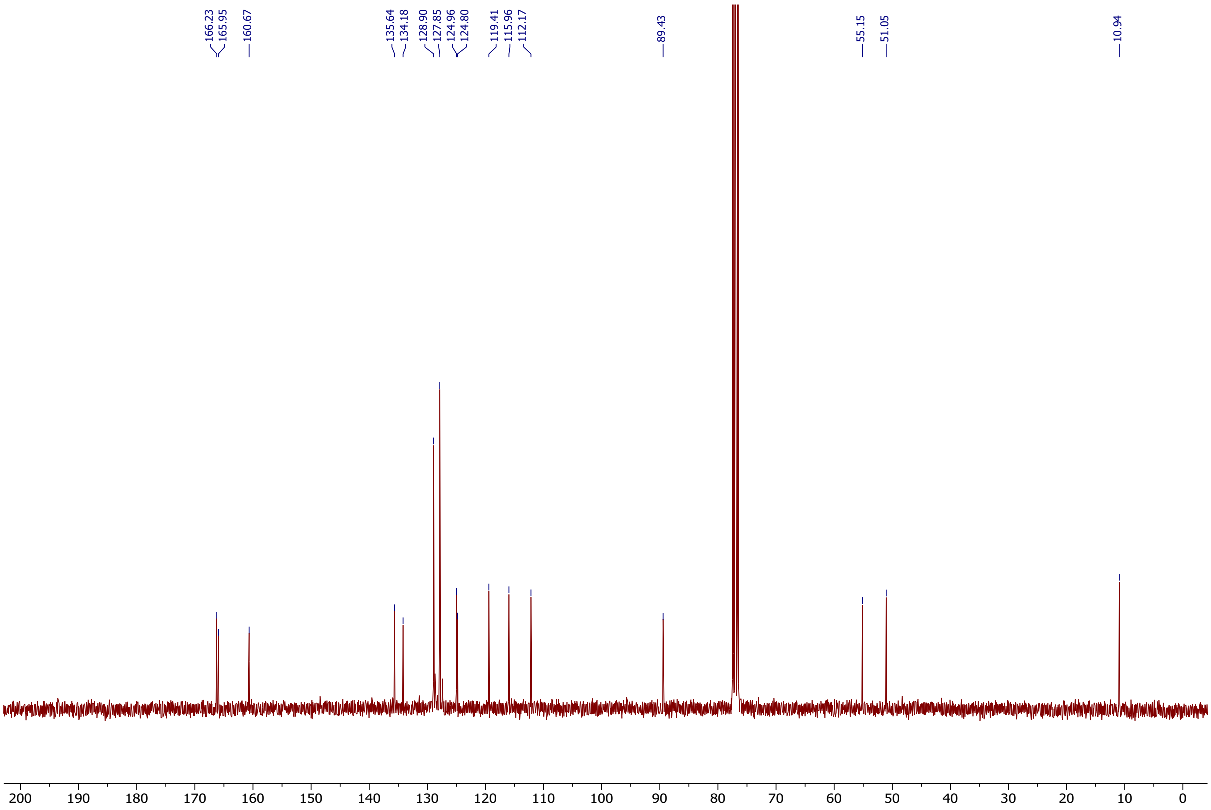

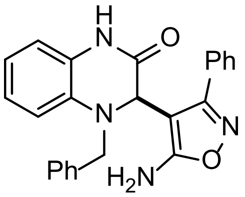

3ab

^{1}H-NMR ($CDCl_3$, 300 MHz)

$^{13}C\{^{1}H\}$ NMR ($CDCl_3$, 75 MHz)

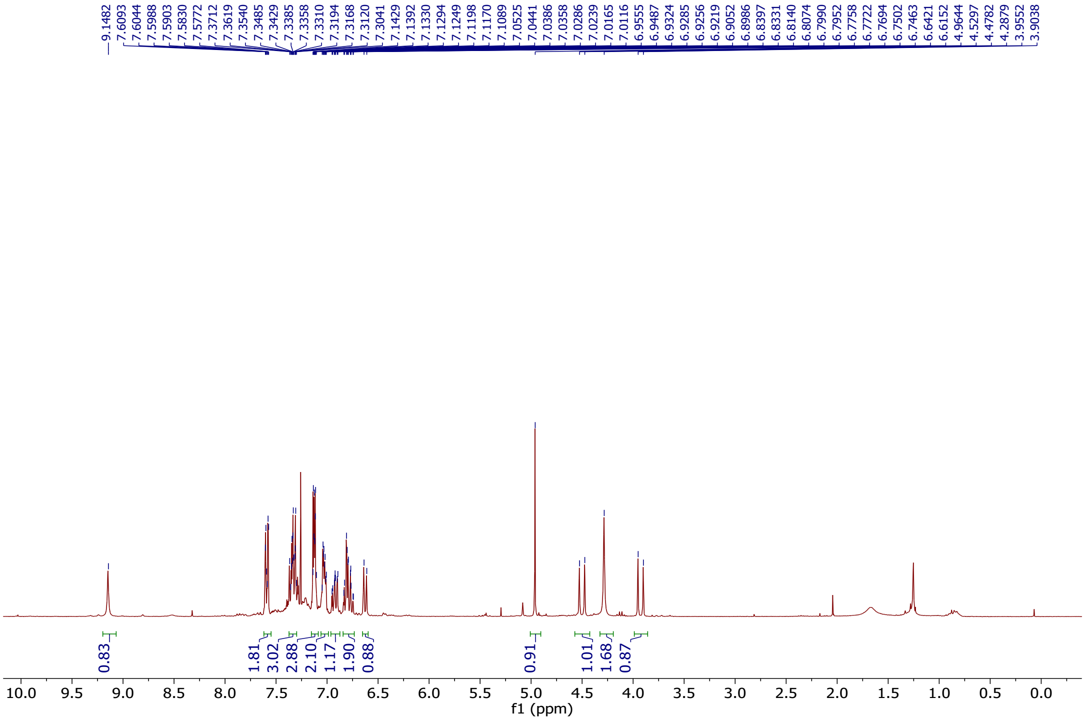

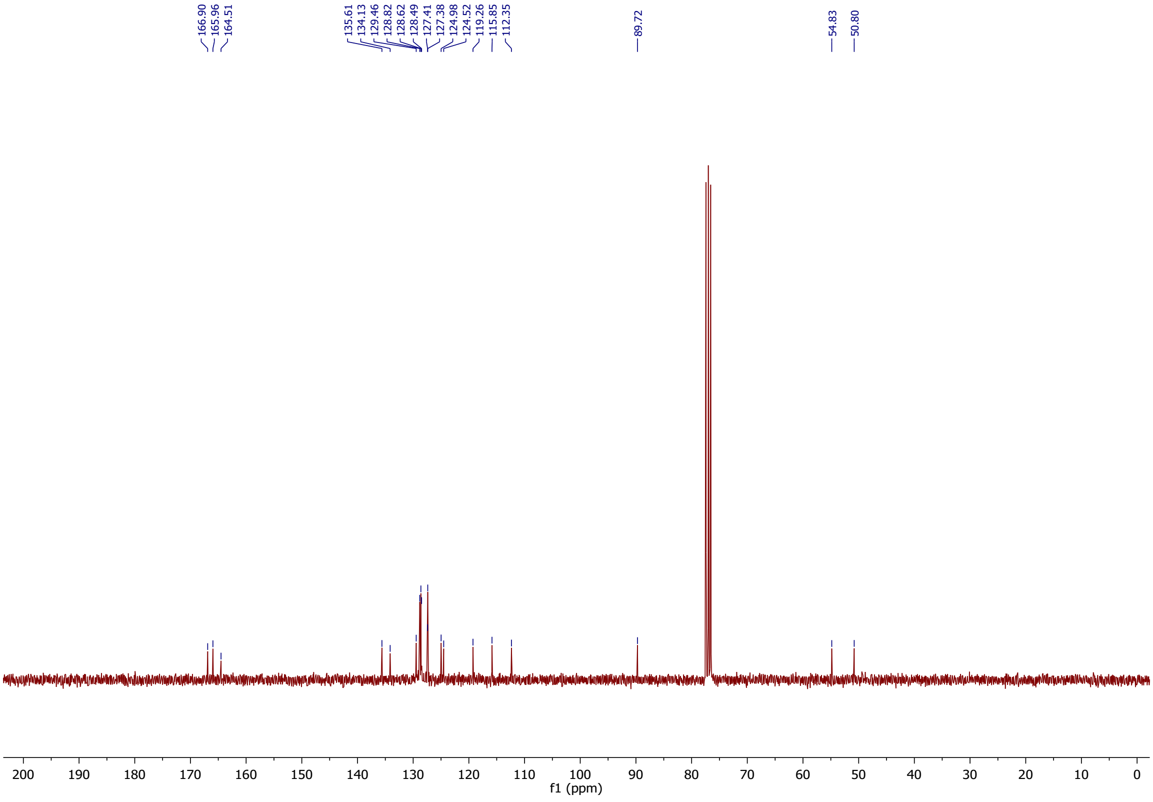

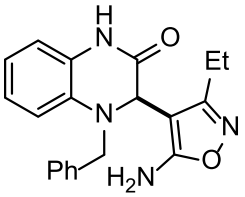

3ac

^{1}H-NMR ($CDCl_3$, 300 MHz)

$^{13}C\{^1H\}$ NMR ($CDCl_3$, 75 MHz)

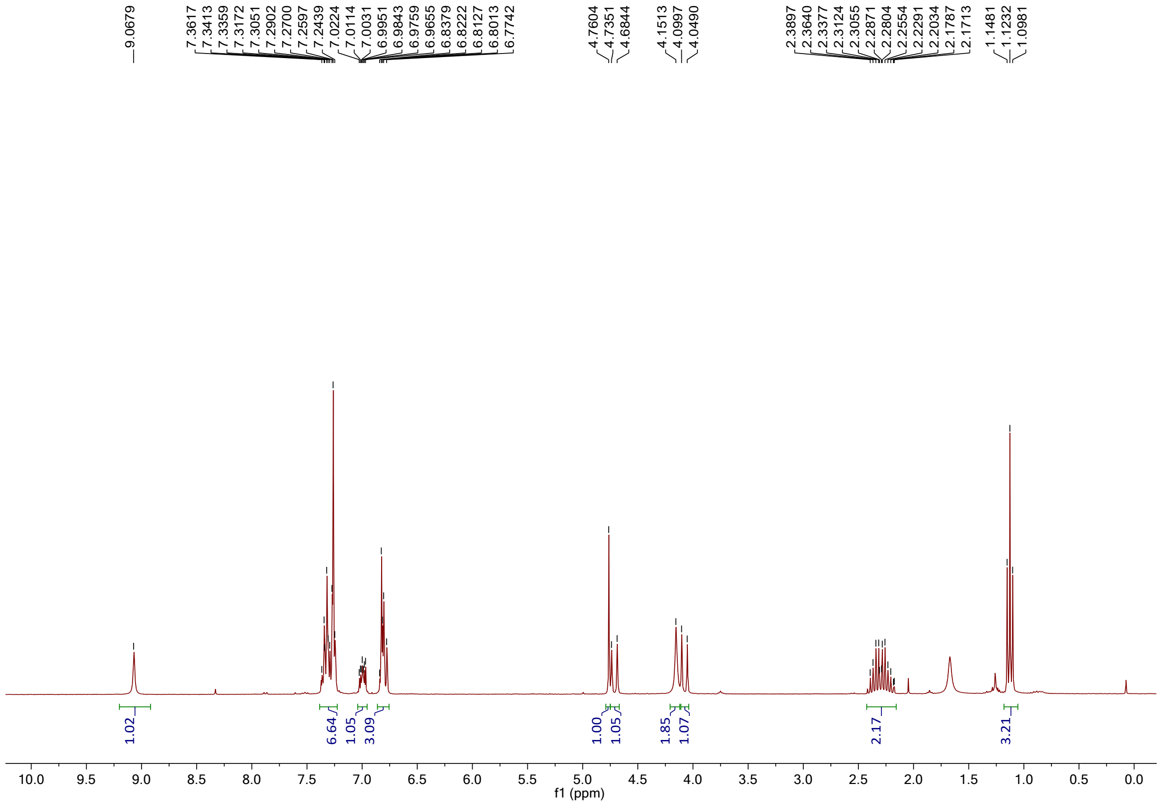

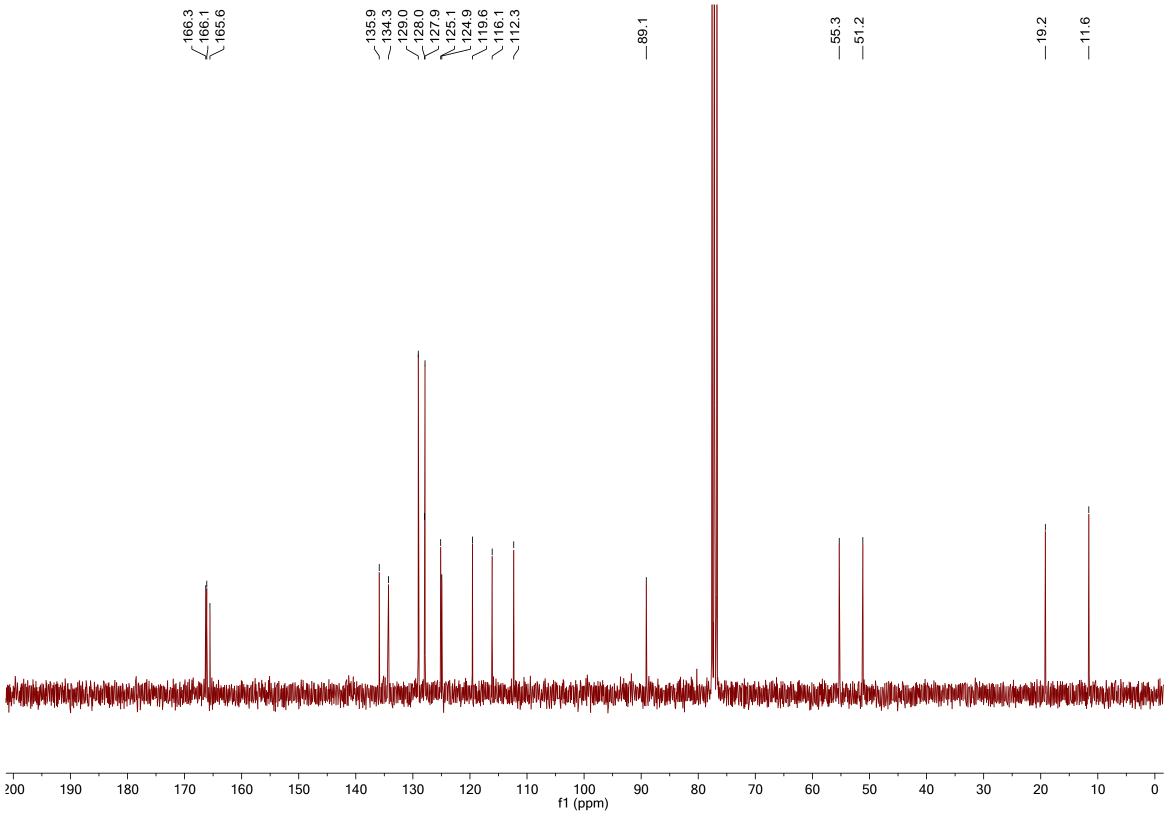

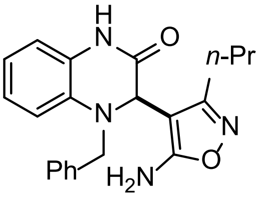

3ad

^{1}H-NMR (CD_3OD, 300 MHz)

^{13}C{^{1}H} NMR (CD_3OD, 75 MHz)

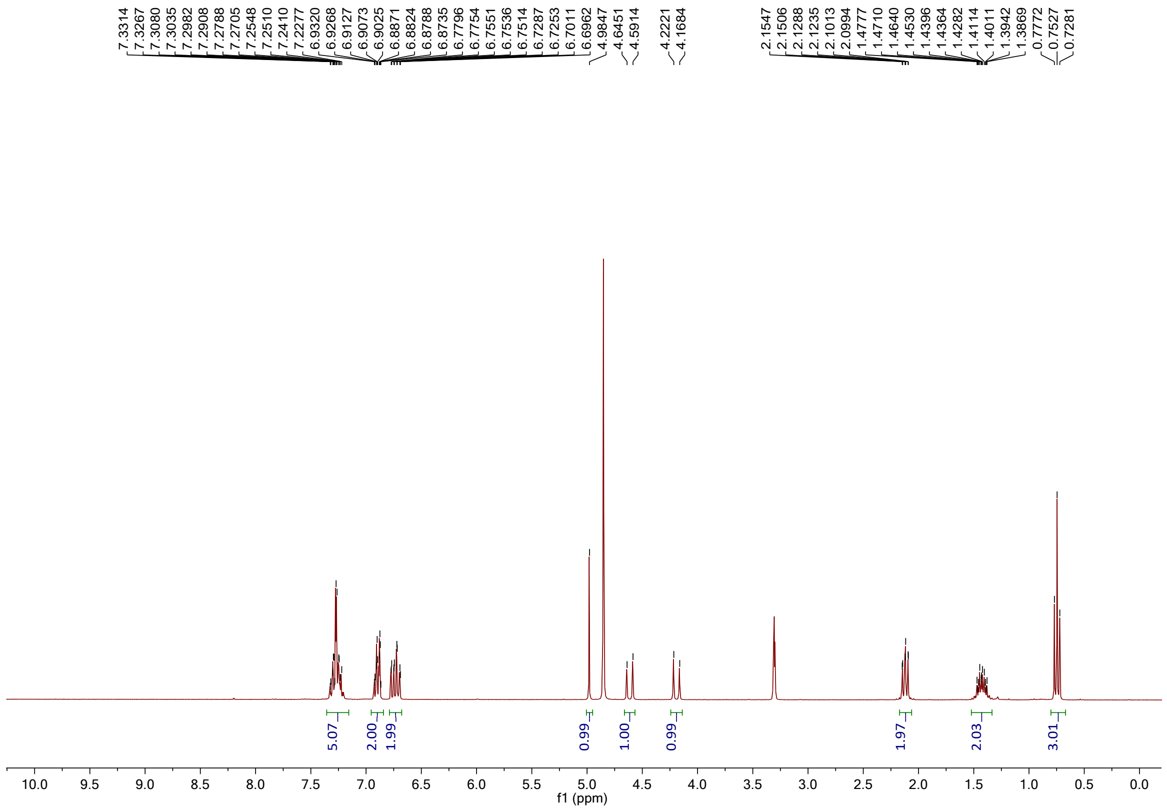

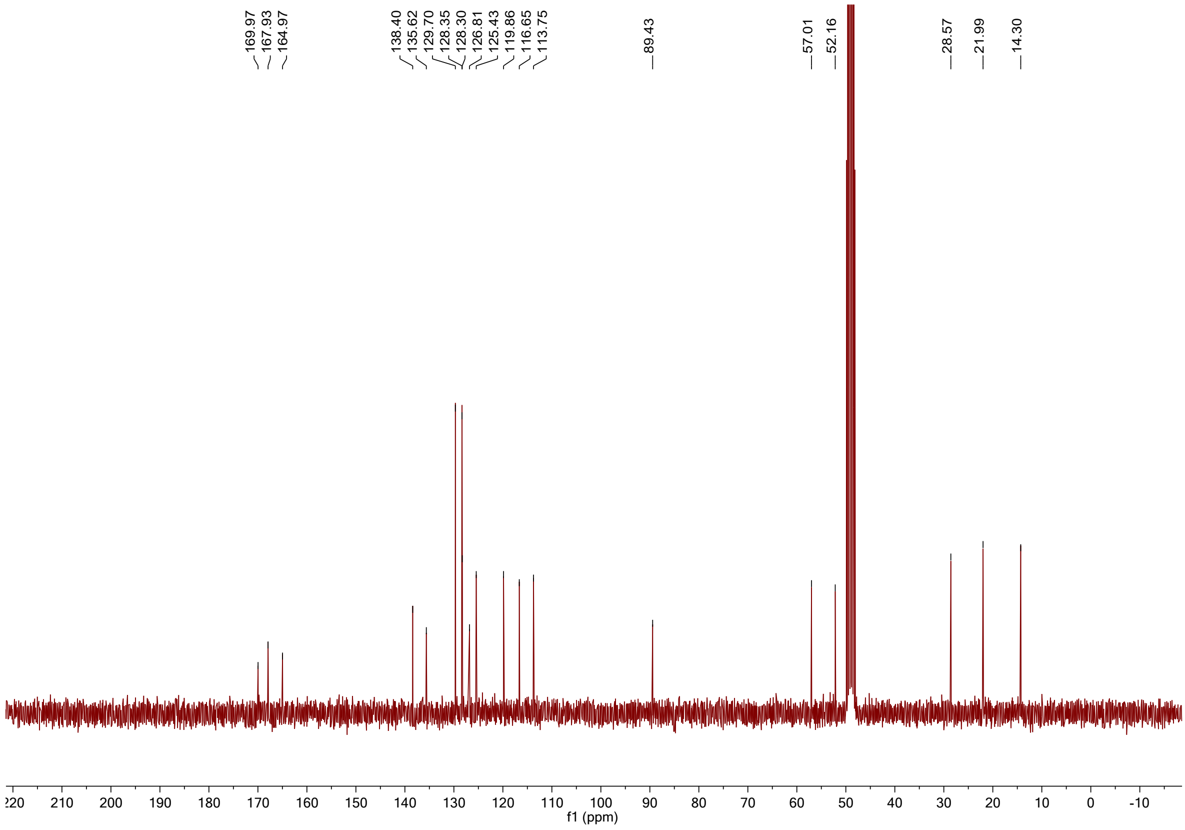

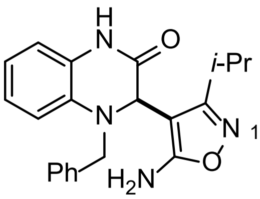

3ae

^{1}H-NMR ($CDCl_3$, 300 MHz)

$^{13}C\{^{1}H\}$ NMR ($CDCl_3$, 75 MHz)

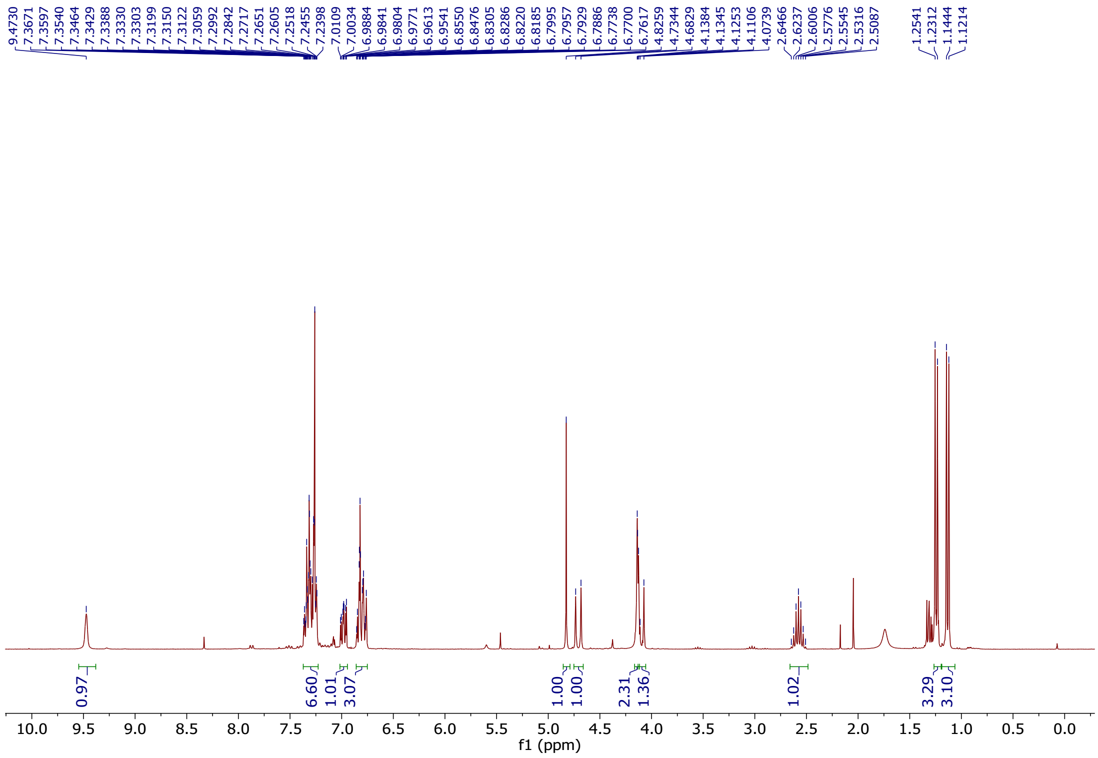

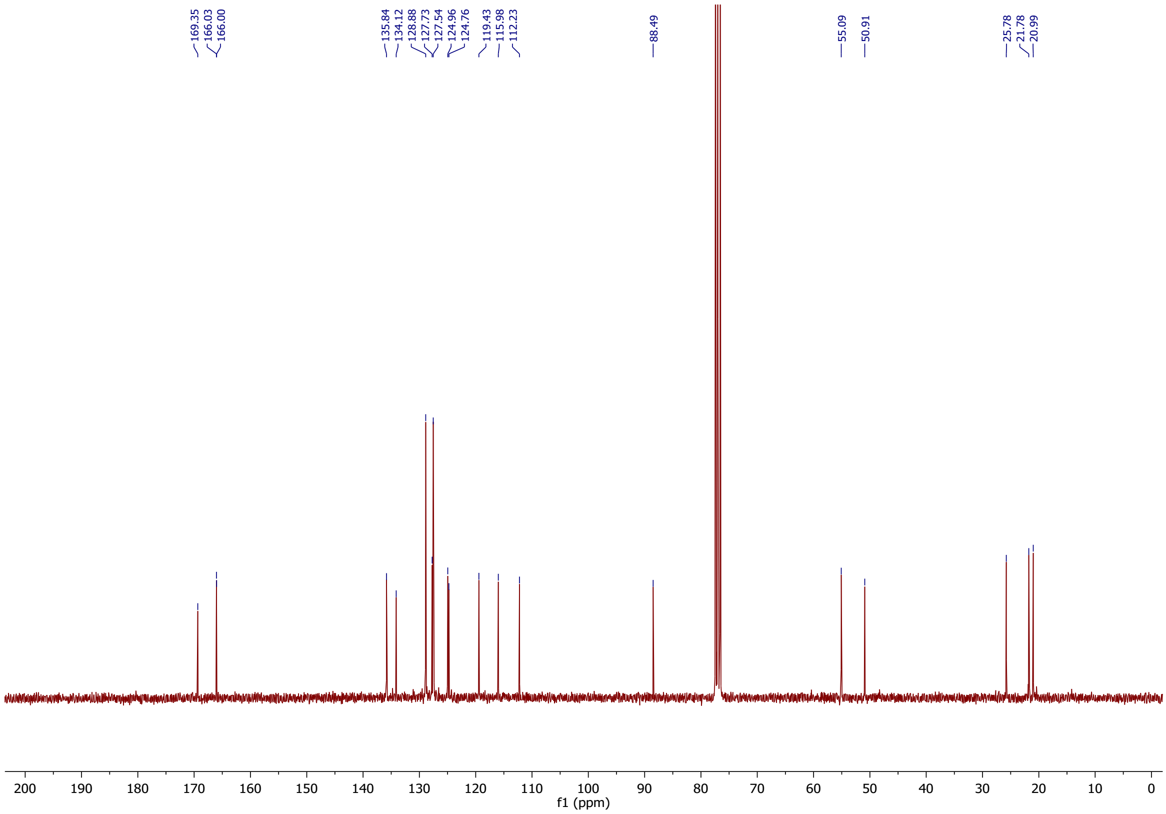

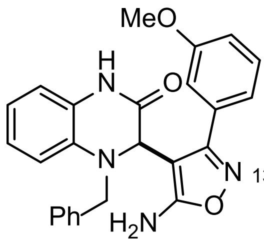

3af

^{1}H-NMR ($CDCl_3$, 300 MHz)

$^{13}C\{^{1}H\}$ NMR ($CDCl_3$, 75 MHz)

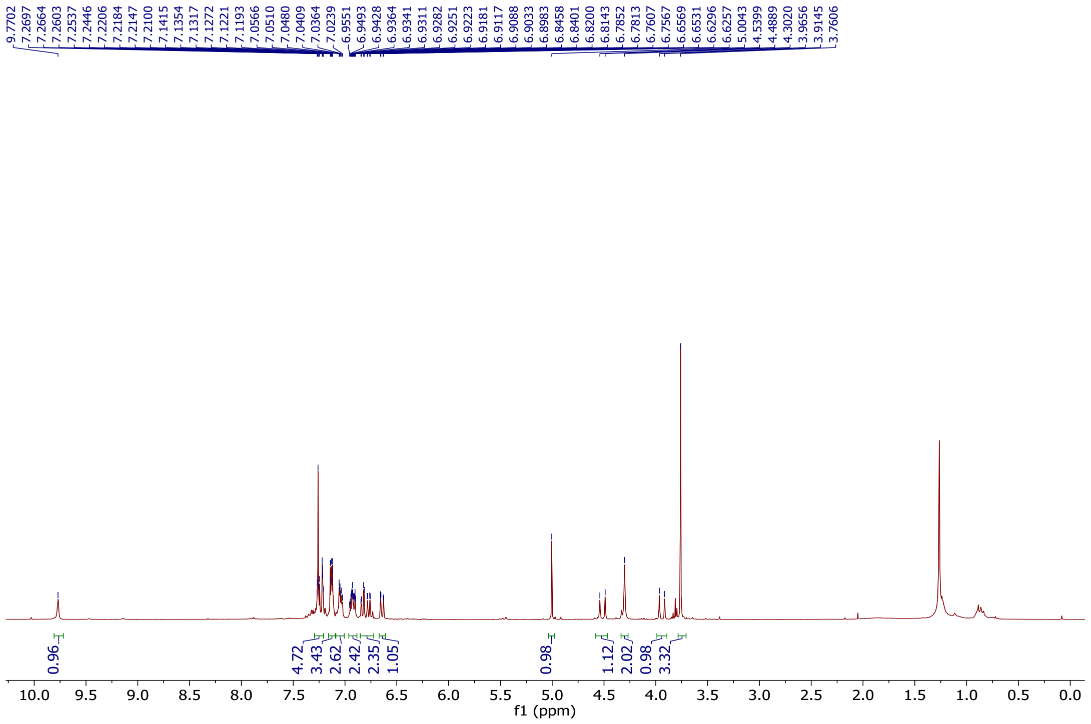

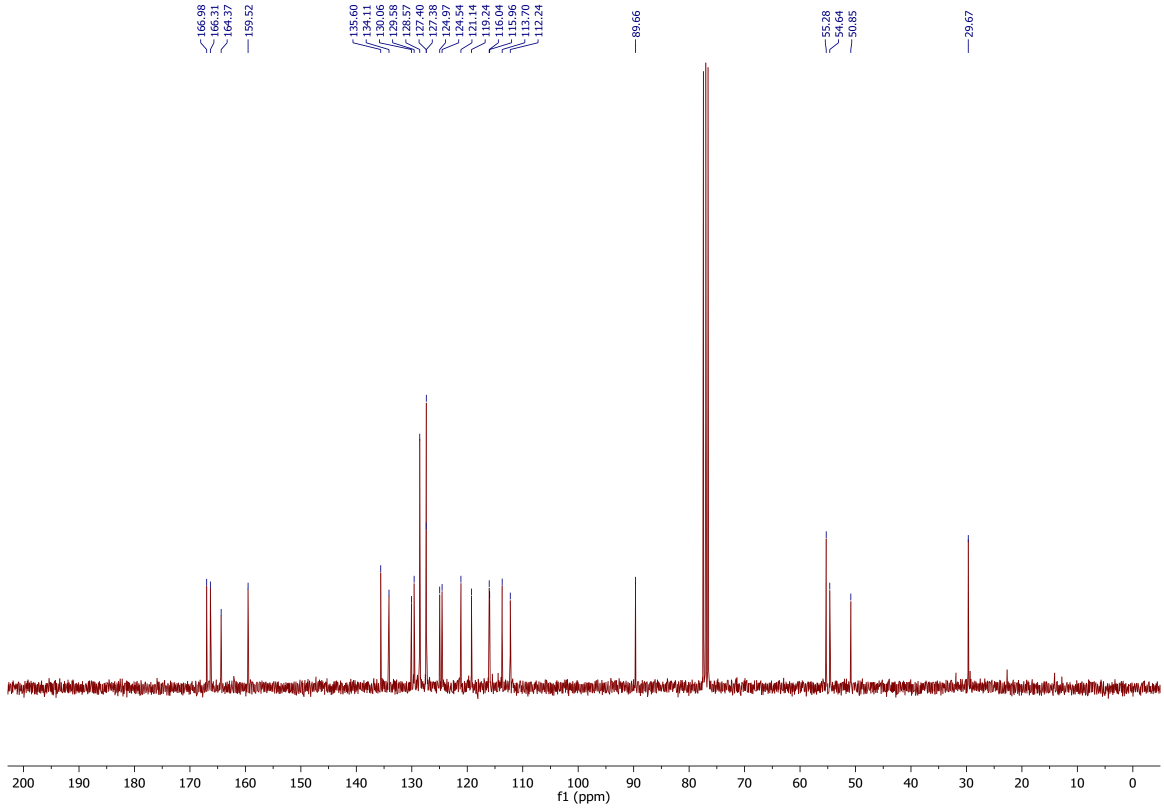

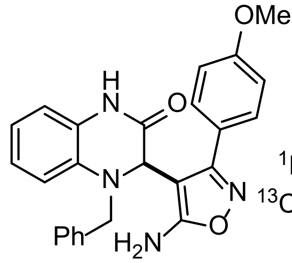

3ag

^{1}H-NMR (DMSO-d_6, 300 MHz)

^{13}C{^{1}H} NMR (DMSO-d_6, 75 MHz)

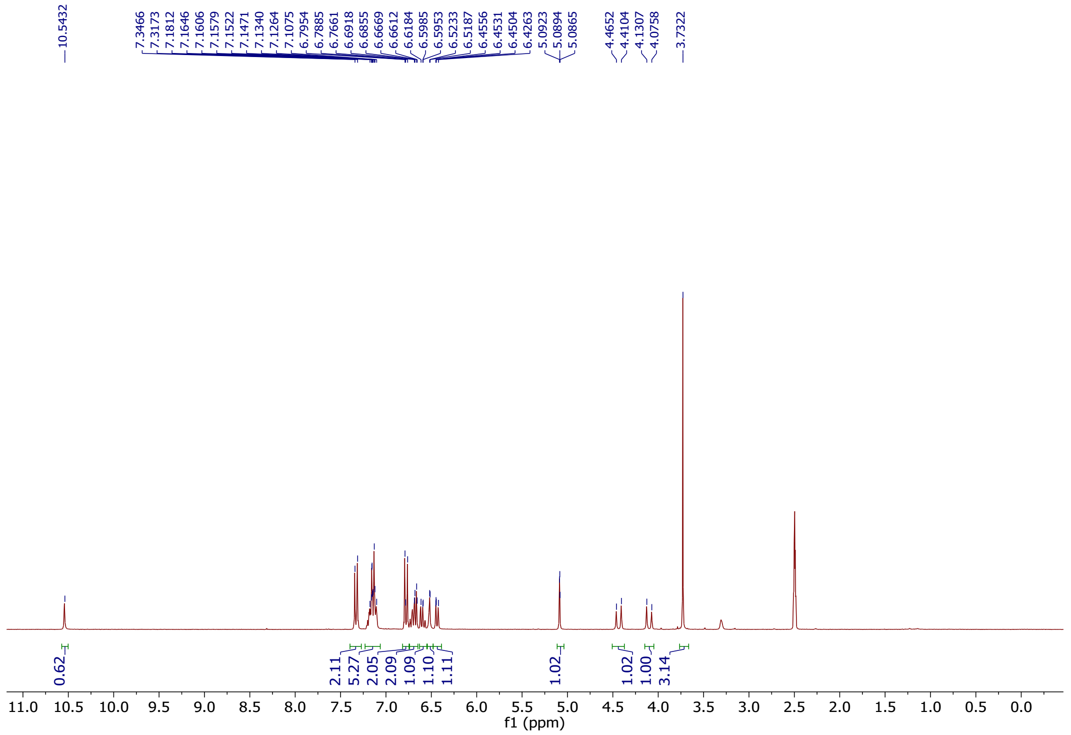

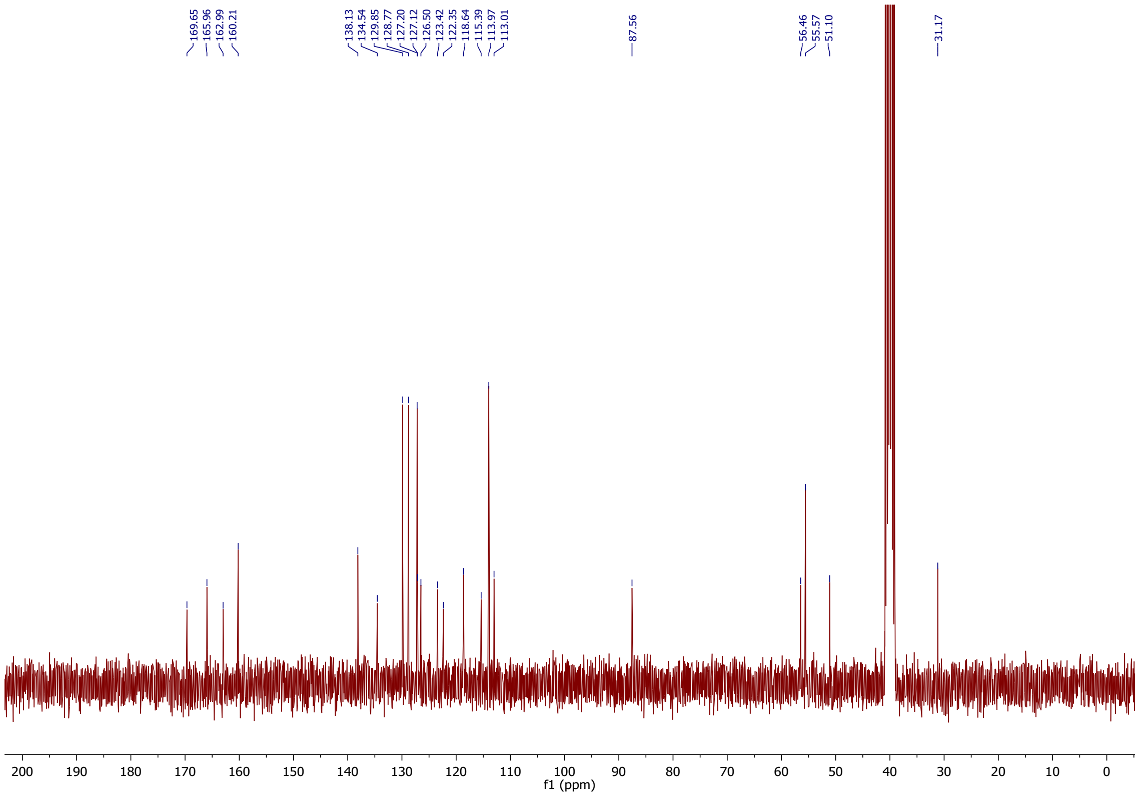

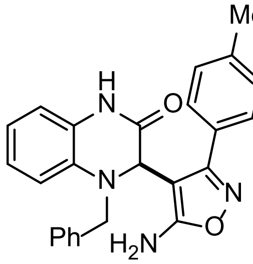

3ah

^{1}H-NMR ($CDCl_3$, 300 MHz)

^{13}C{^{1}H} NMR ($CDCl_3$, 75 MHz)

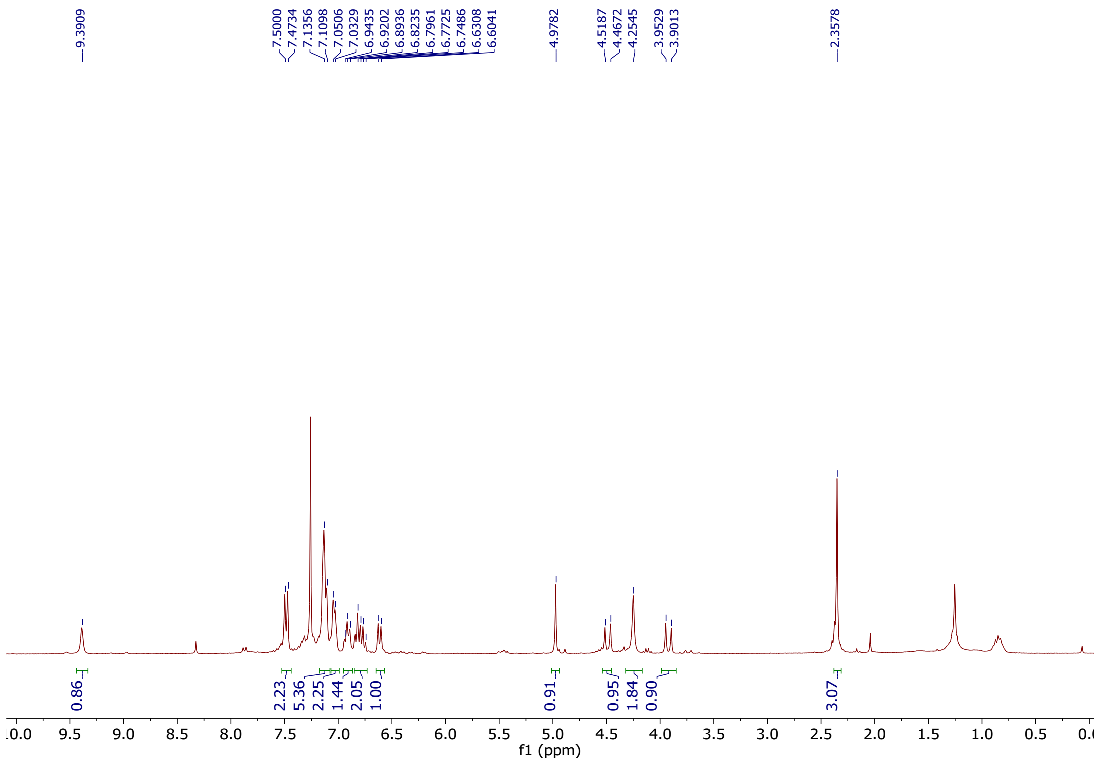

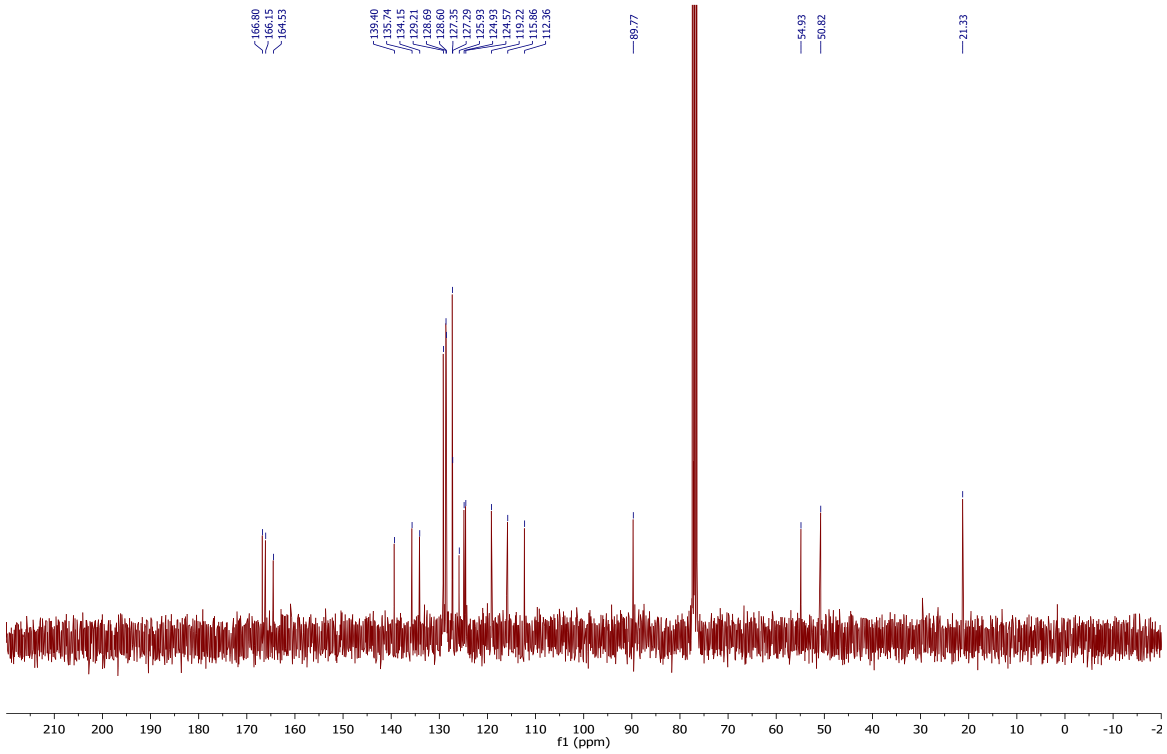

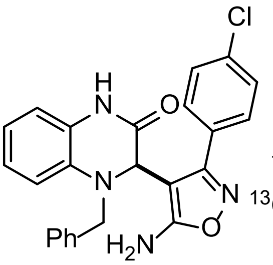

3ai

^{1}H-NMR (DMSO-d_6, 300 MHz)

^{13}C{^{1}H} NMR (DMSO-d_6, 75 MHz)

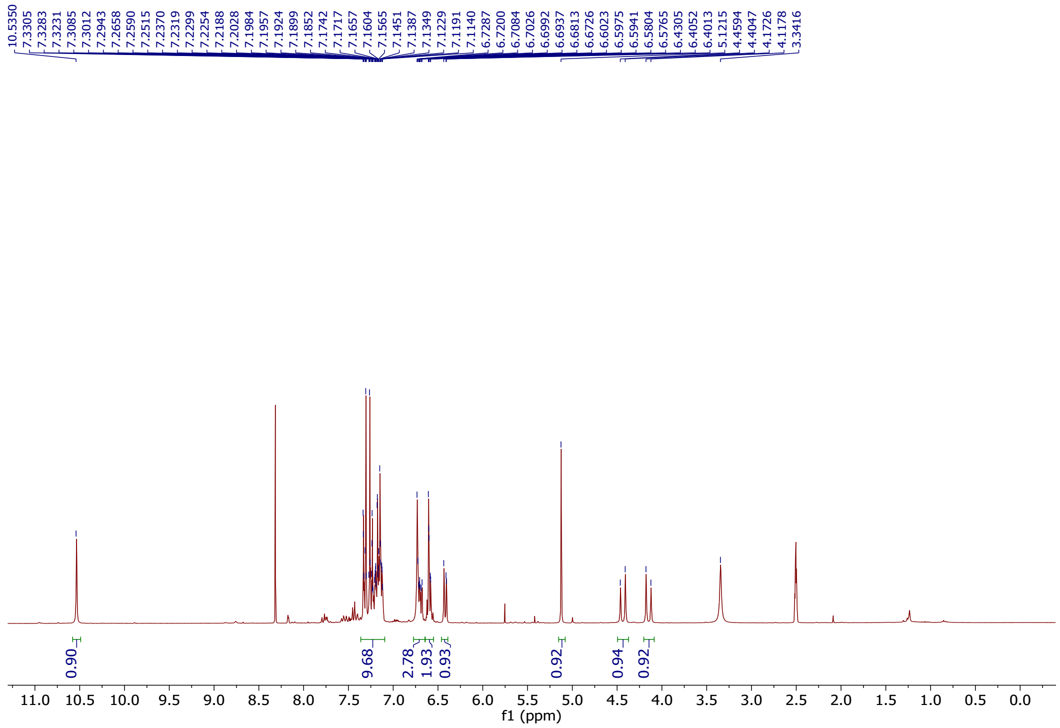

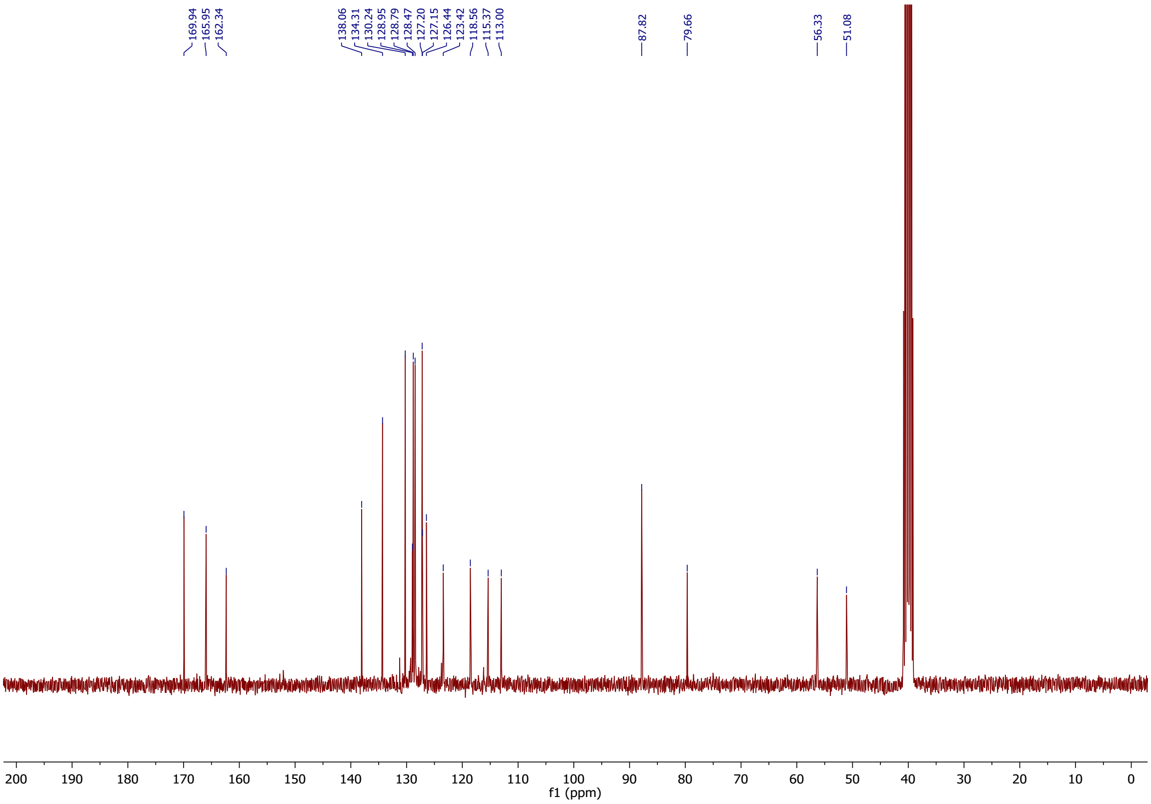

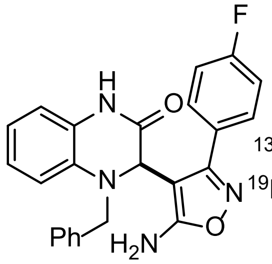

3aj

^{1}H-NMR (DMSO-d_6, 300 MHz)

^{13}C{^{1}H} NMR (DMSO-d_6, 75 MHz)

^{19}F{^{1}H} NMR (DMSO-d_6, 282 MHz)

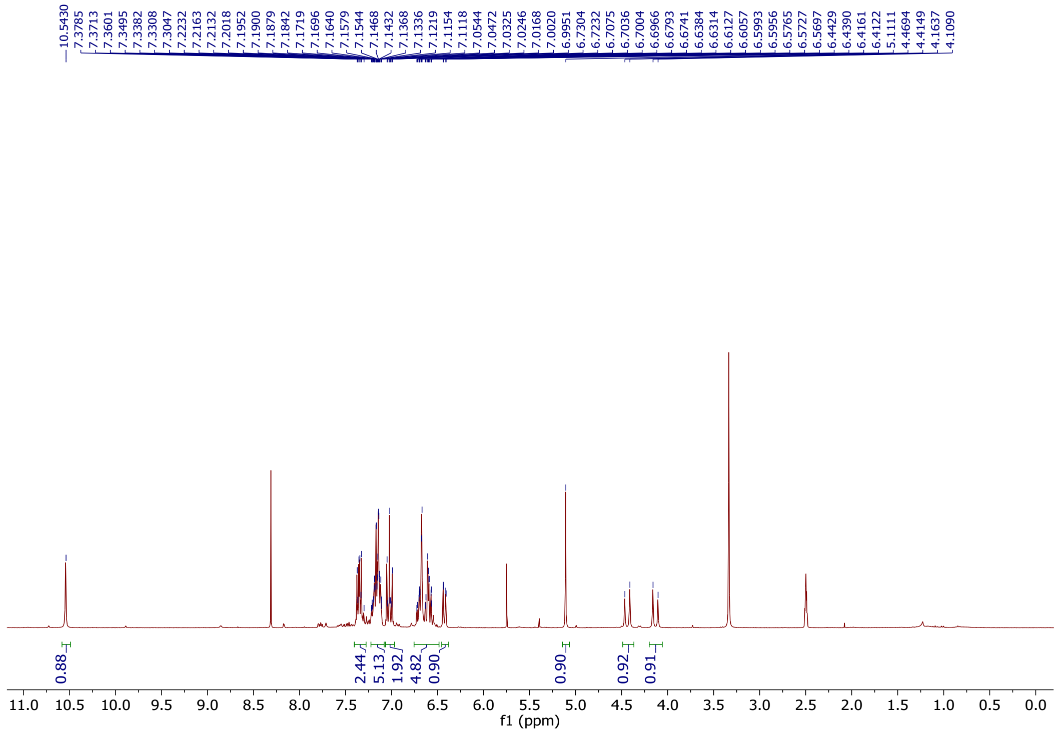

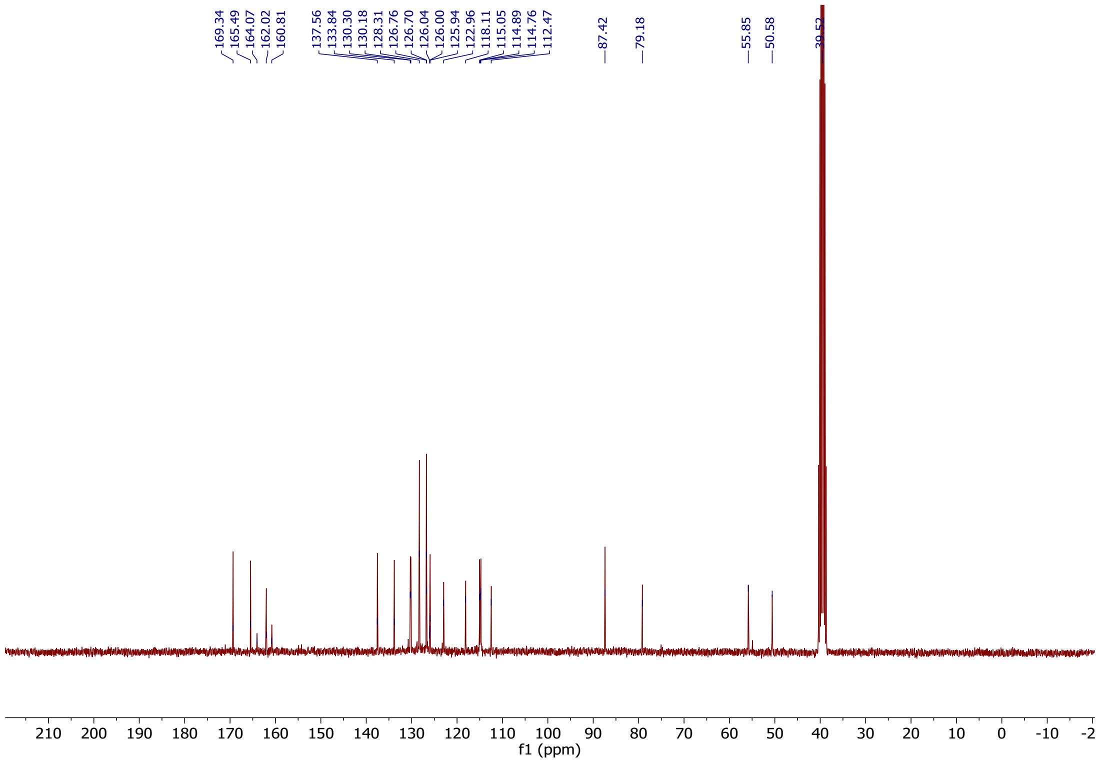

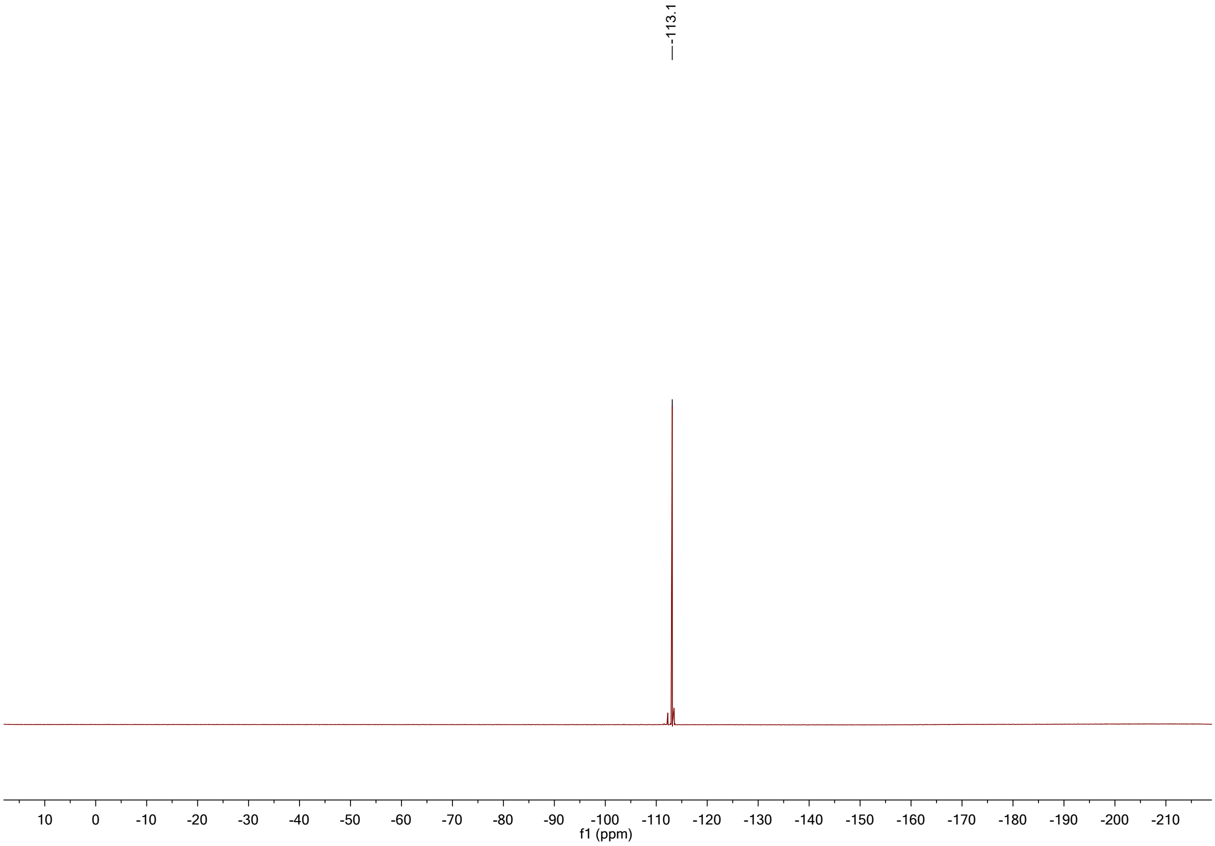
-113.1
10
0
-10
-20
-30
-40
-50
-60
-70
-80
-90
-100
-110
-120
-130
-140
-150
-160
-170
-180
-190
-200
-210
f1 (ppm)

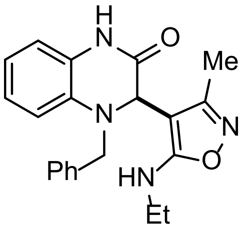

3ak

^{1}H-NMR ($CDCl_3$, 300 MHz)

^{13}C{^{1}H} NMR ($CDCl_3$, 75 MHz)

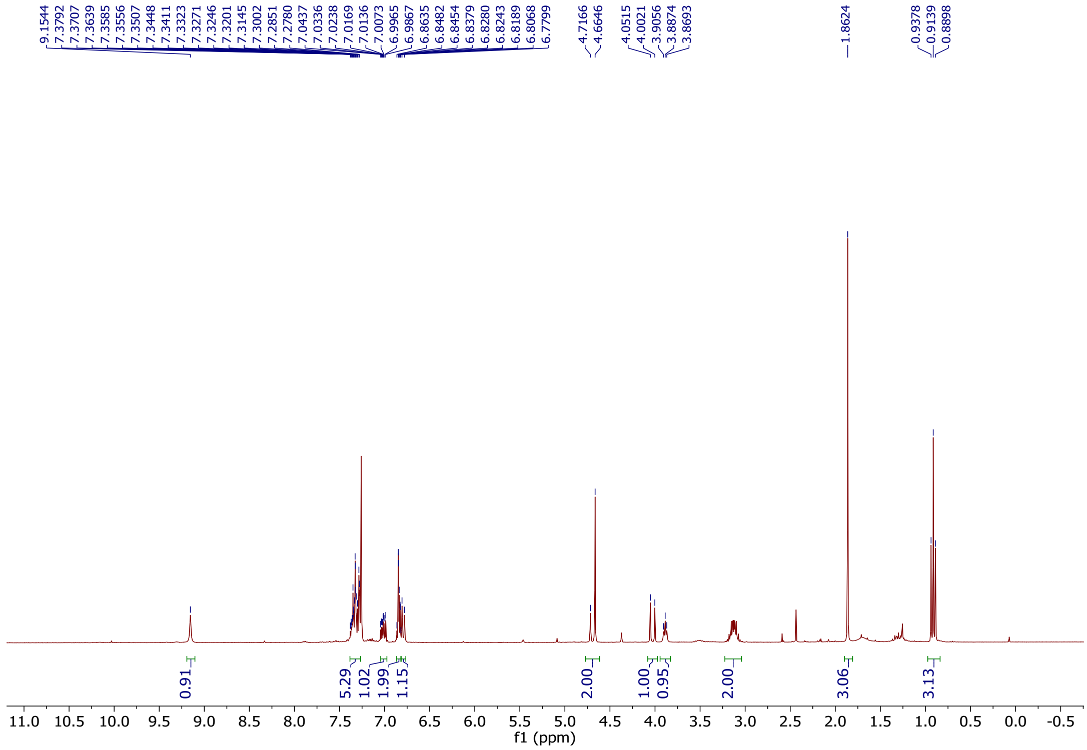

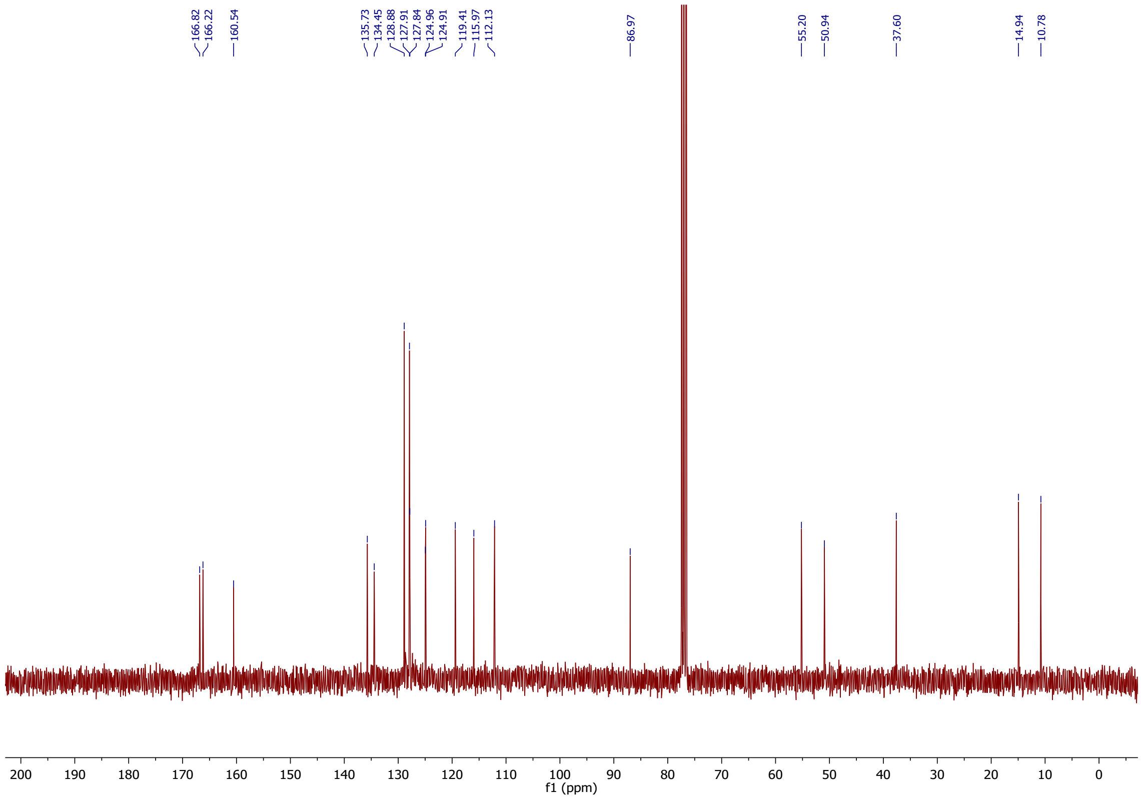

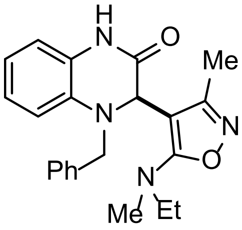

3al
^{1}H-NMR ($CDCl_3$, 300 MHz)
$^{13}C\{^{1}H\}$ NMR ($CDCl_3$, 75 MHz)

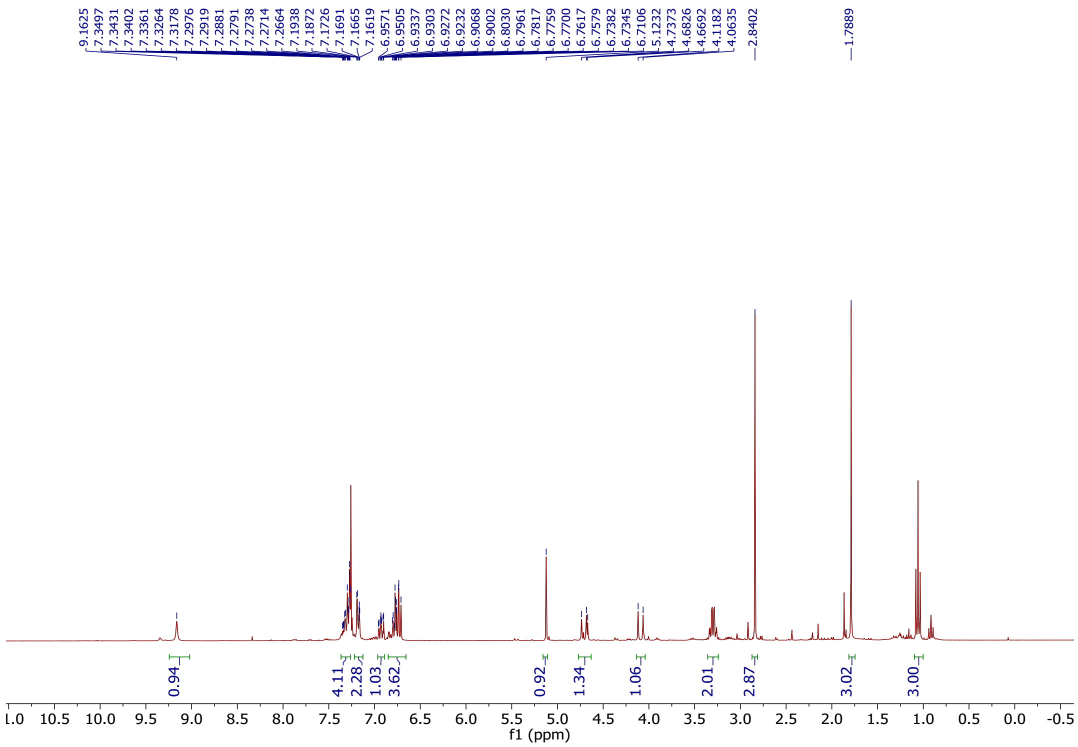

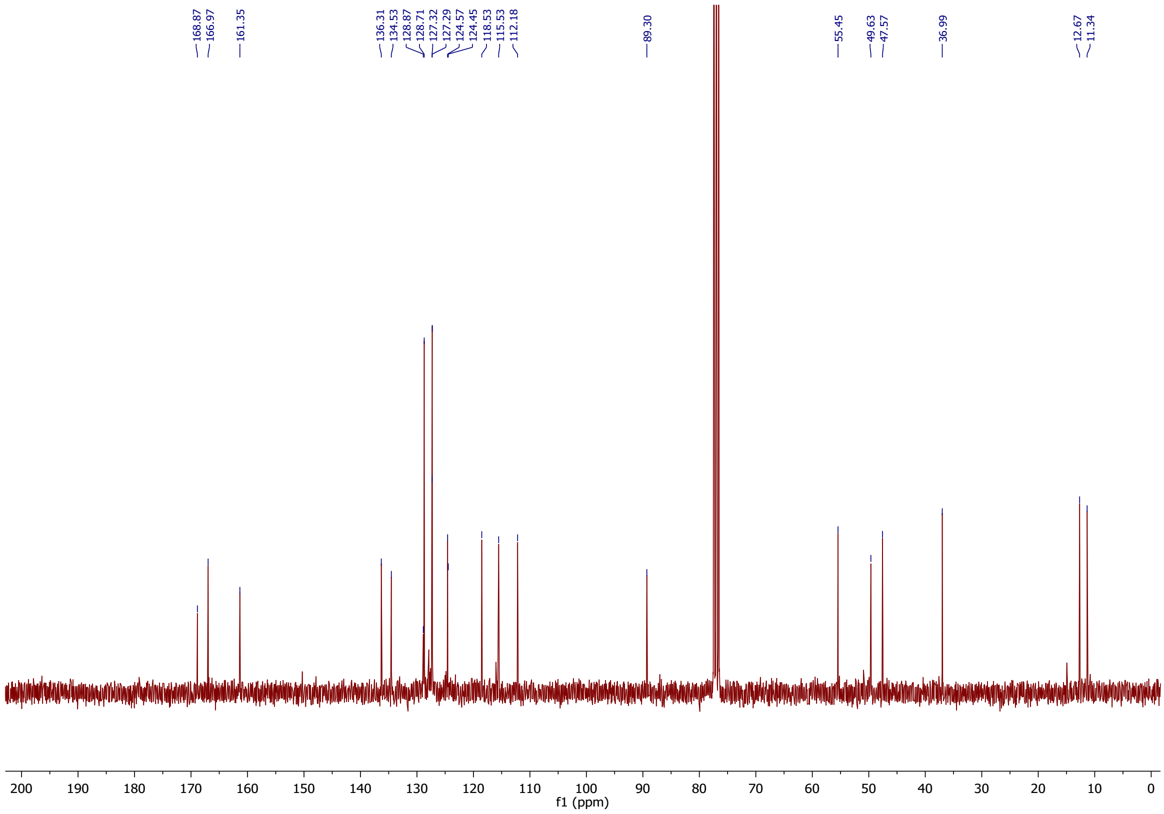

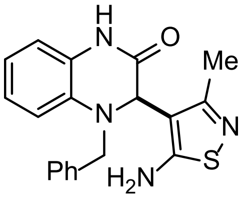

3am

^{1}H-NMR ($CDCl_3$, 300 MHz)

^{13}C{^{1}H} NMR ($CDCl_3$, 75 MHz)

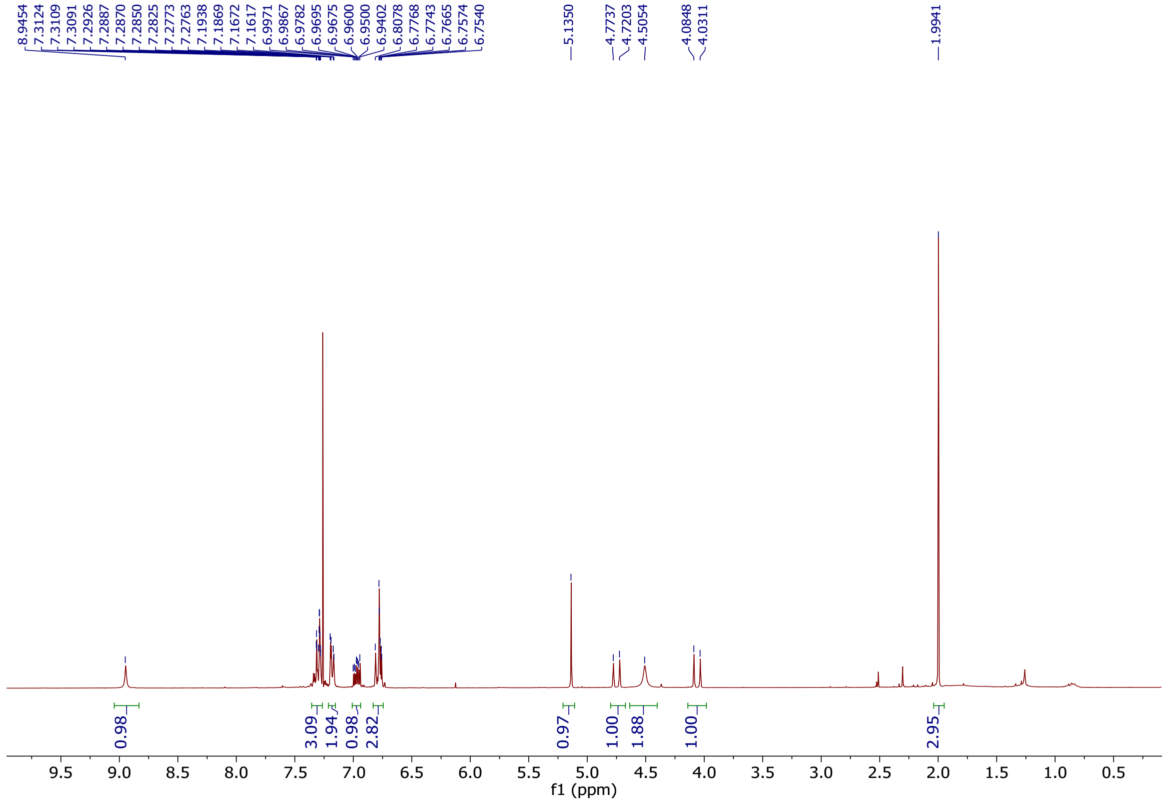

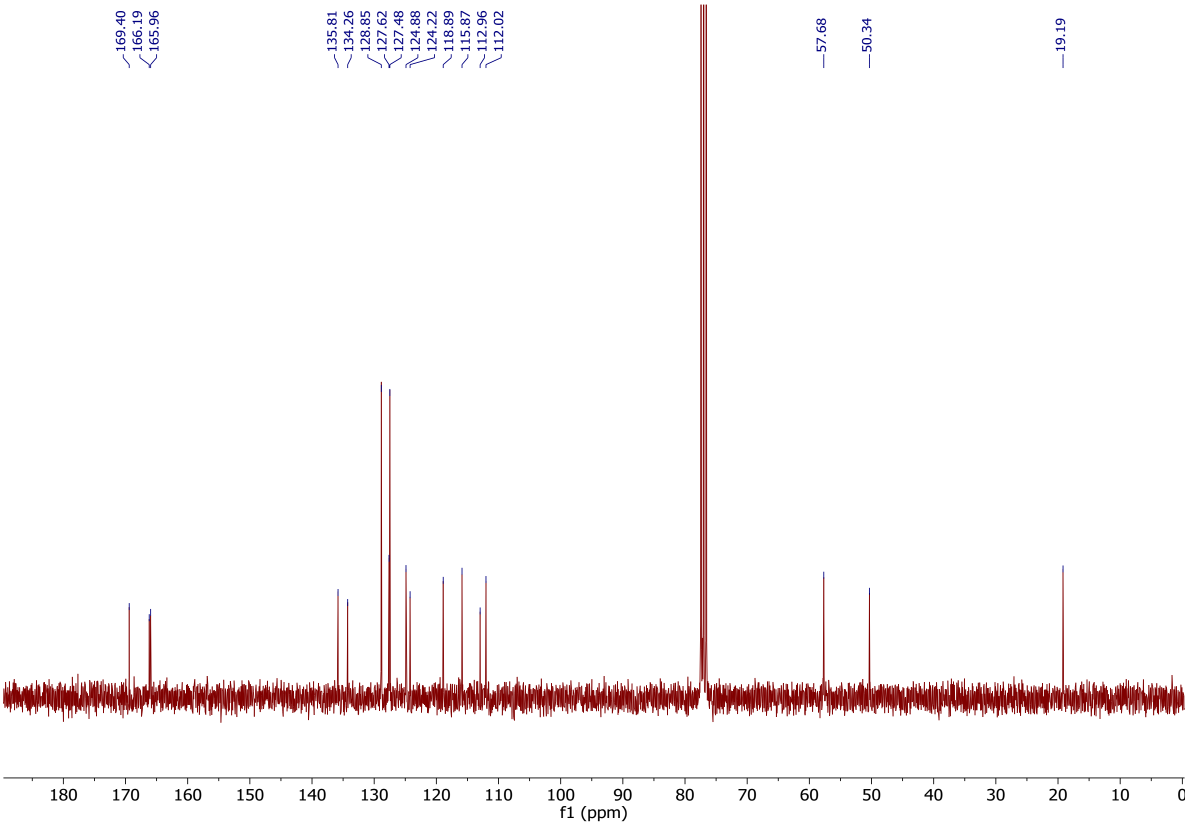

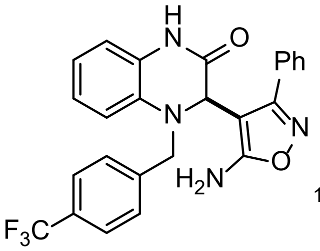

3bb

^{1}H-NMR (DMSO-d_6, 300 MHz)

^{13}C{^{1}H} NMR (DMSO-*d6*, 75 MHz)

^{19}F{^{1}H} NMR (DMSO-*d6*, 282 MHz)

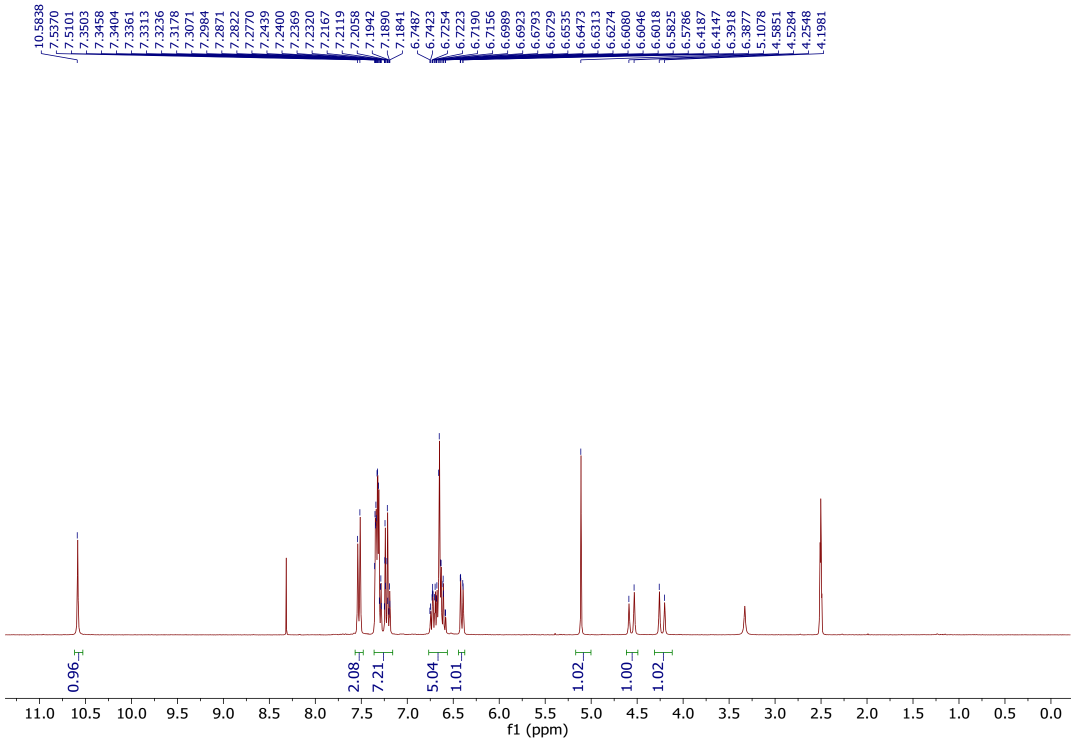

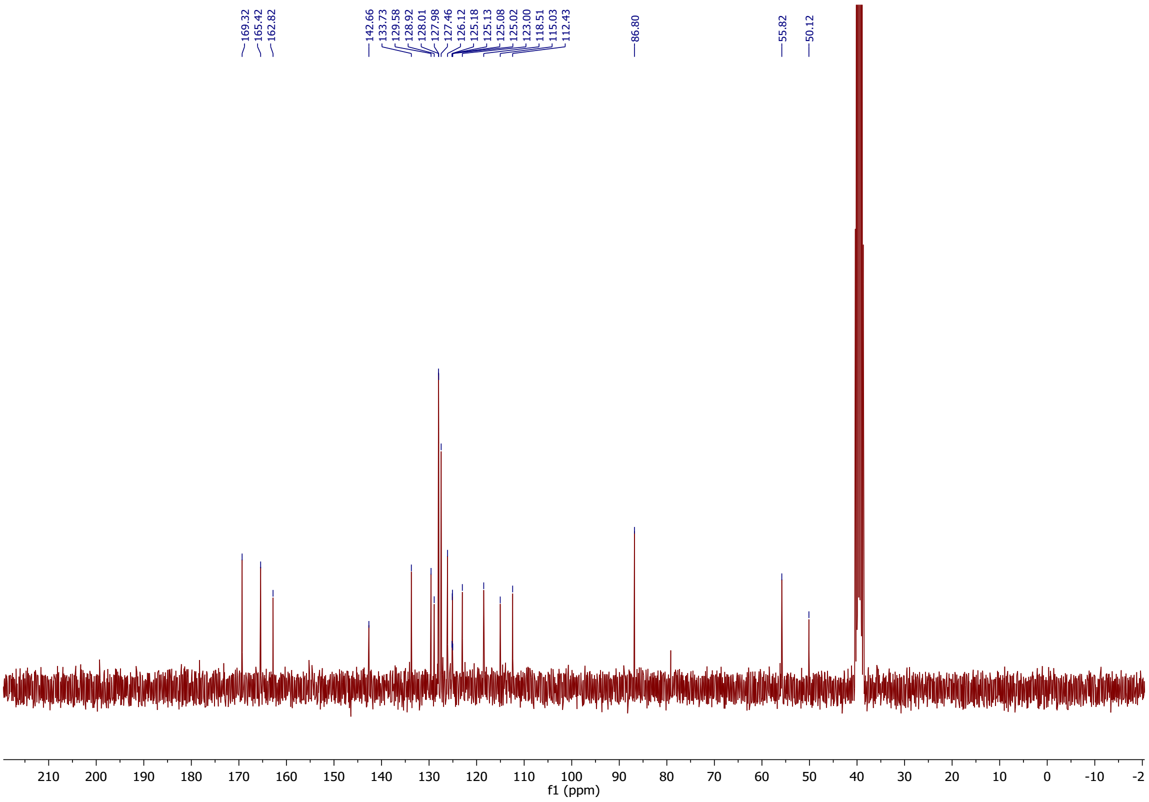

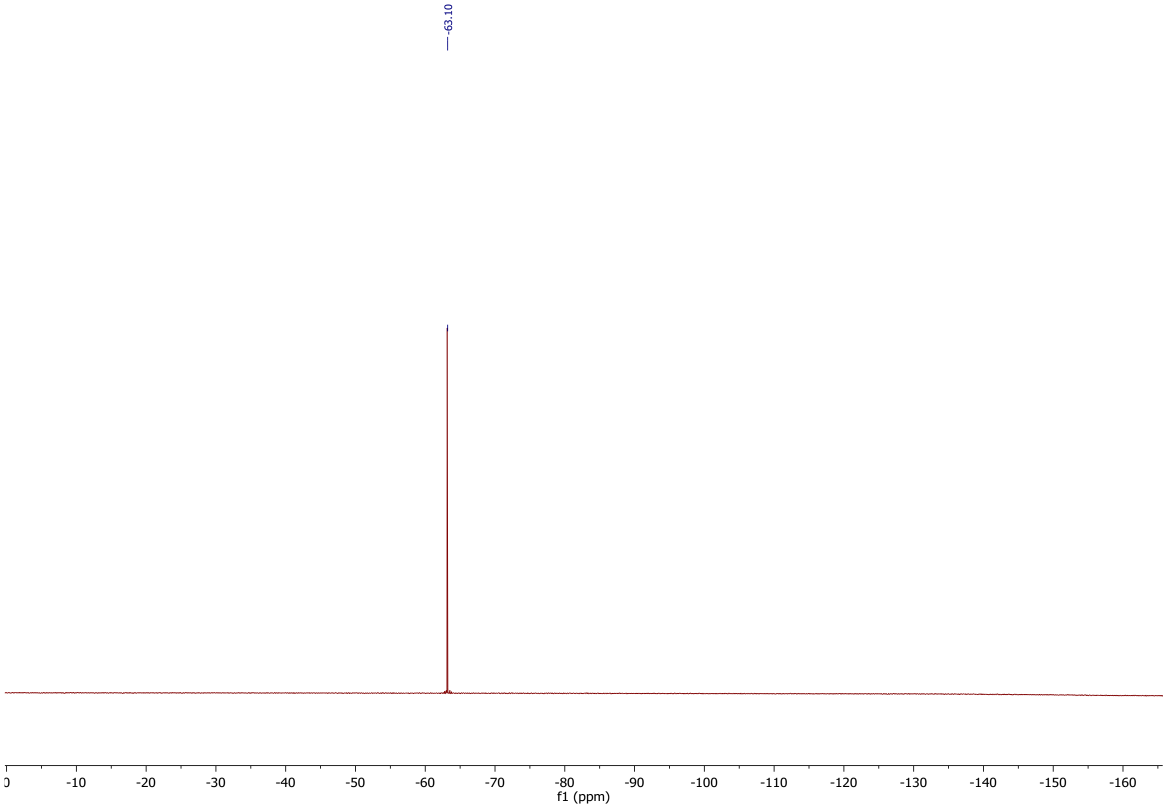

-63.10
0
-10
-20
-30
-40
-50
-60
-70
-80
-90
-100
-110
-120
-130
-140
-150
-160
f1 (ppm)

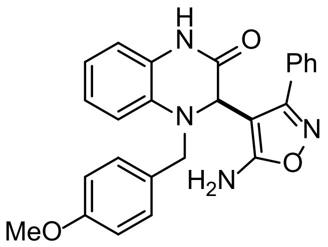

3cb

^{1}H-NMR ($CDCl_3$, 300 MHz)

^{13}C{^{1}H} NMR ($CDCl_3$, 75 MHz)

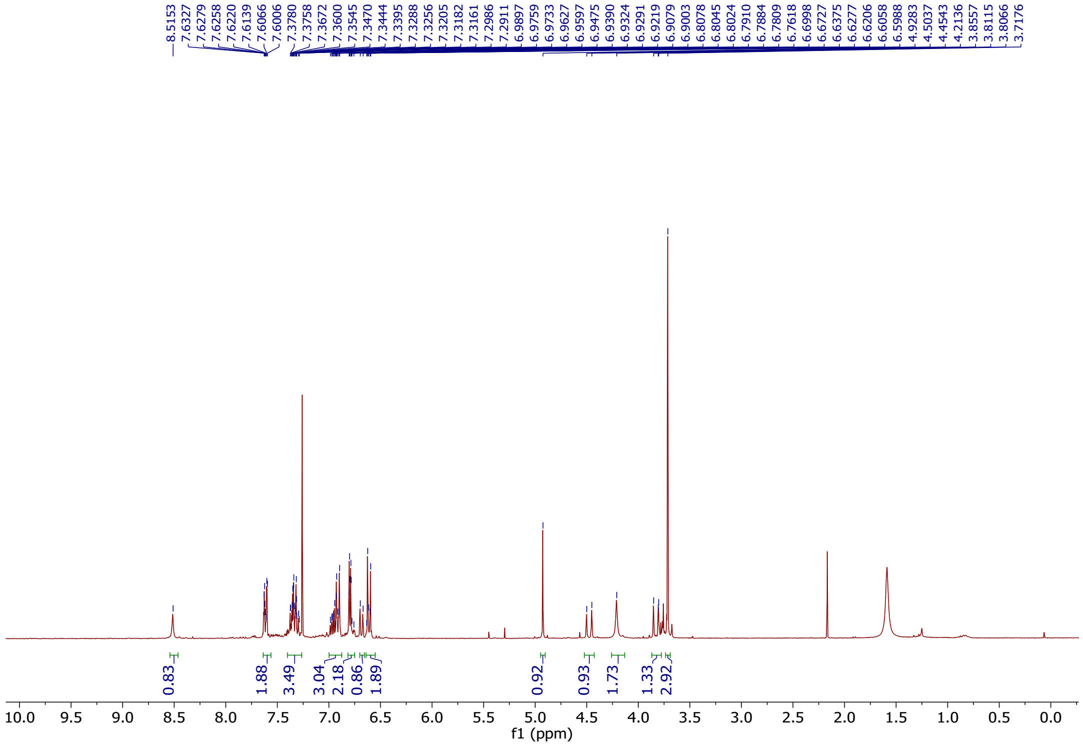

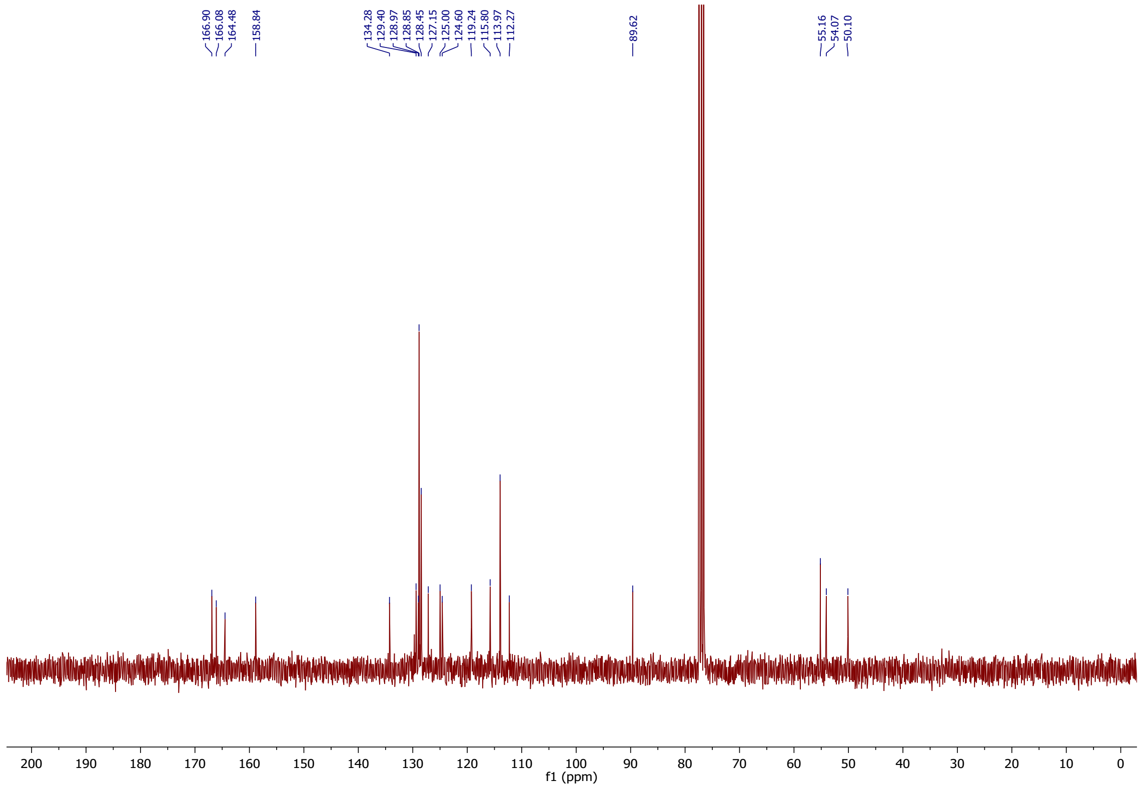

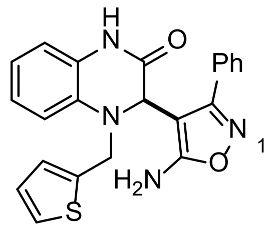

3db

^{1}H-NMR (DMSO-d_6, 300 MHz)

^{13}C{^{1}H} NMR (DMSO-d_6, 75 MHz)

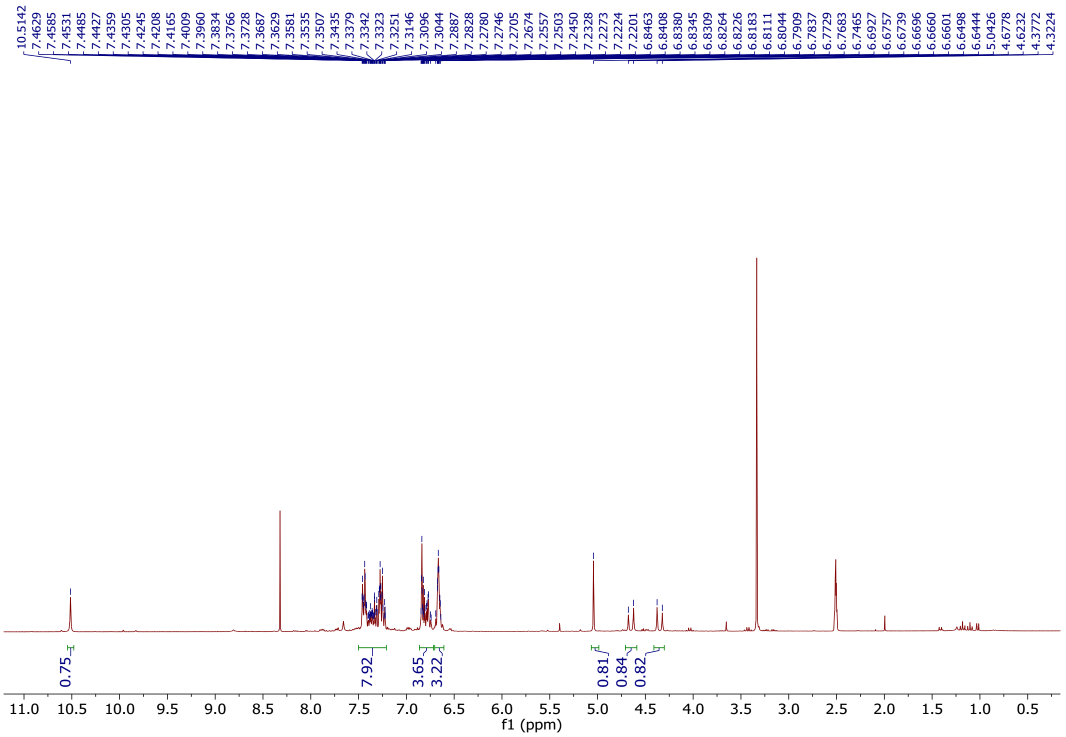

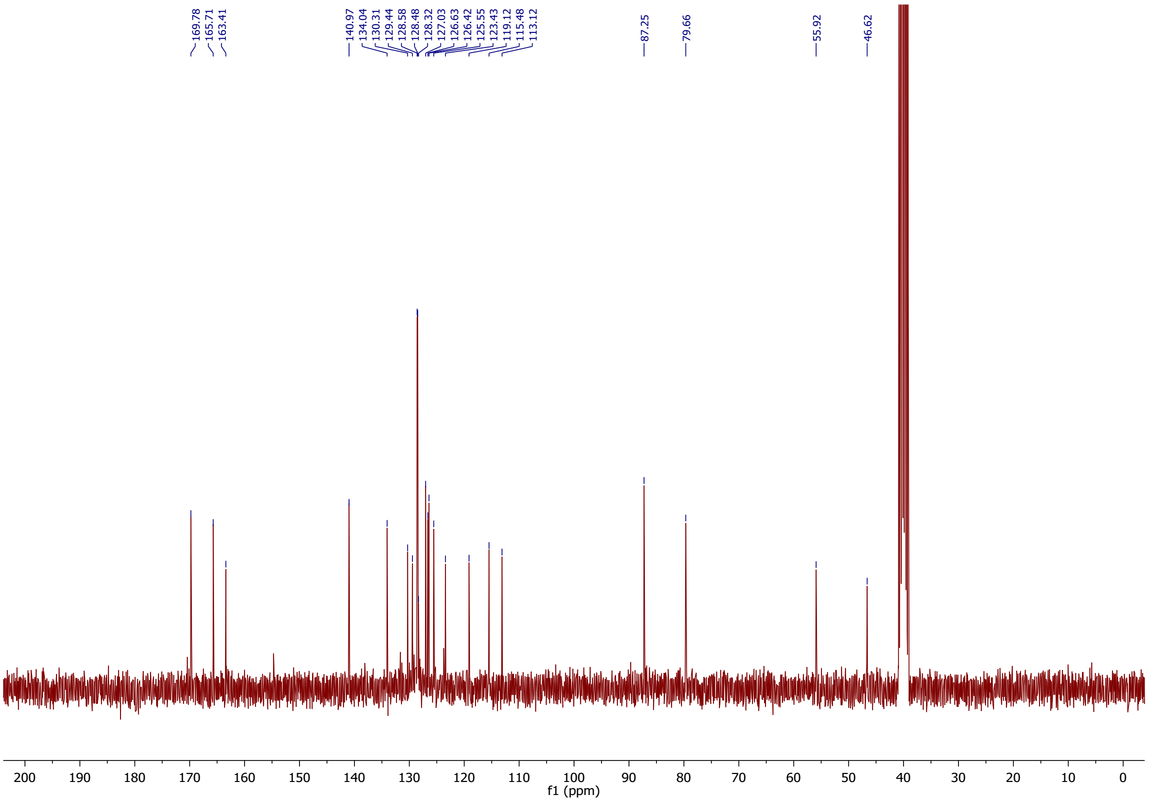

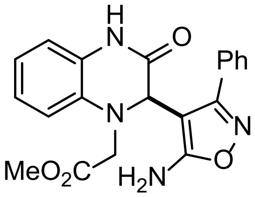

3eb

^{1}H-NMR (DMSO-d_6, 300 MHz)

^{13}C{^{1}H} NMR (DMSO-d_6, 75 MHz)

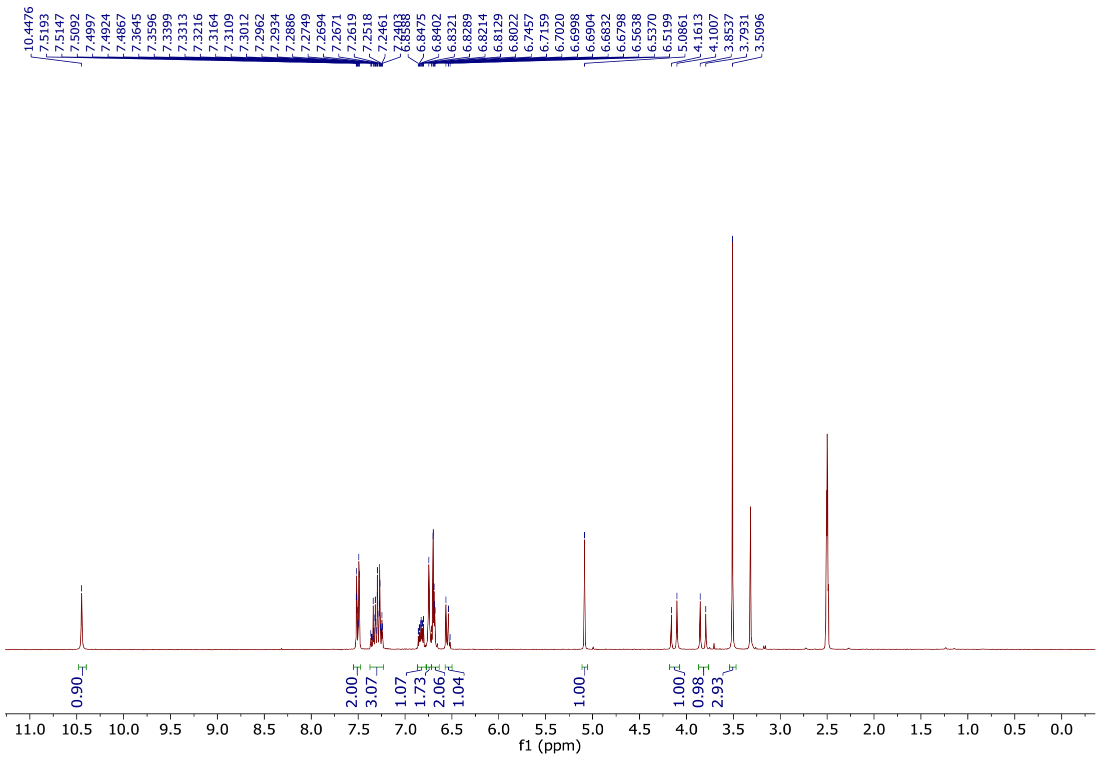

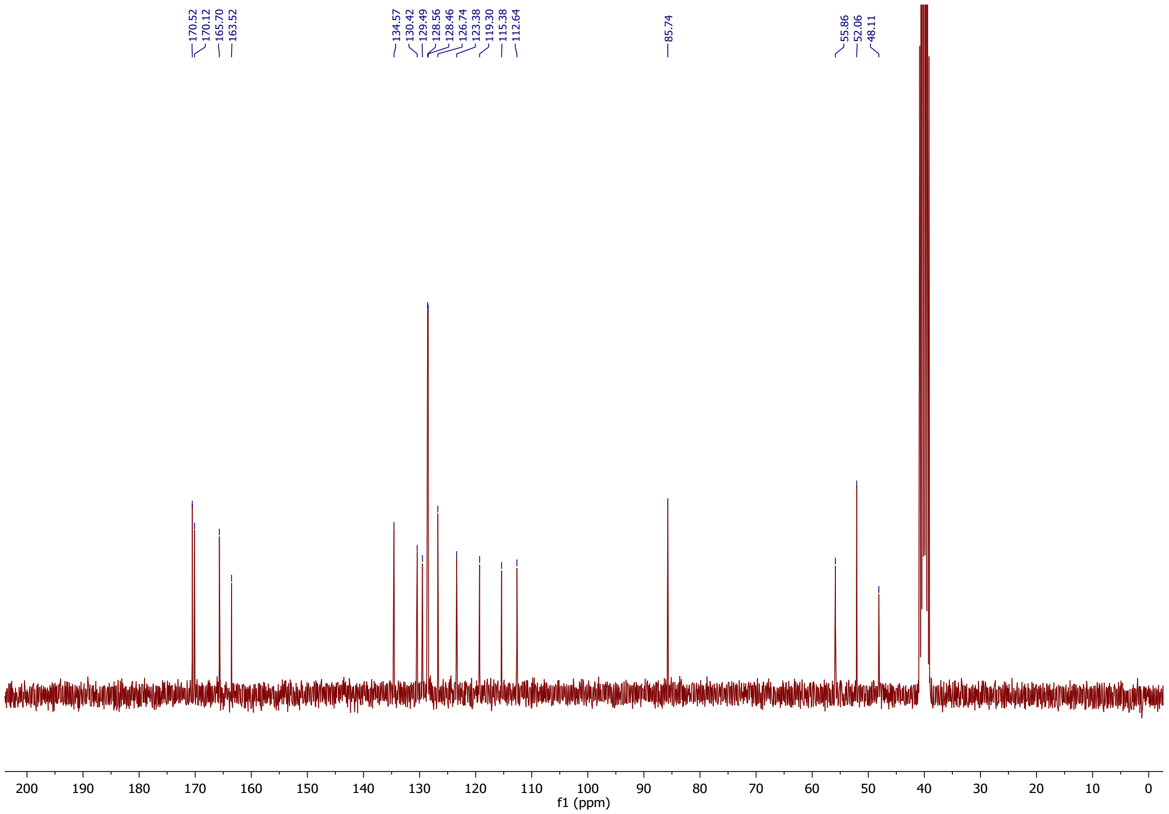

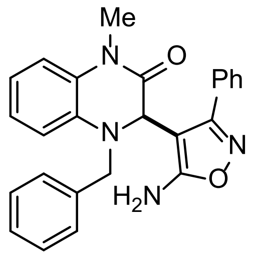

3fb

^{1}H-NMR ($CDCl_3$, 300 MHz)

^{13}C{^{1}H} NMR (DMSO-d_6, 75 MHz)

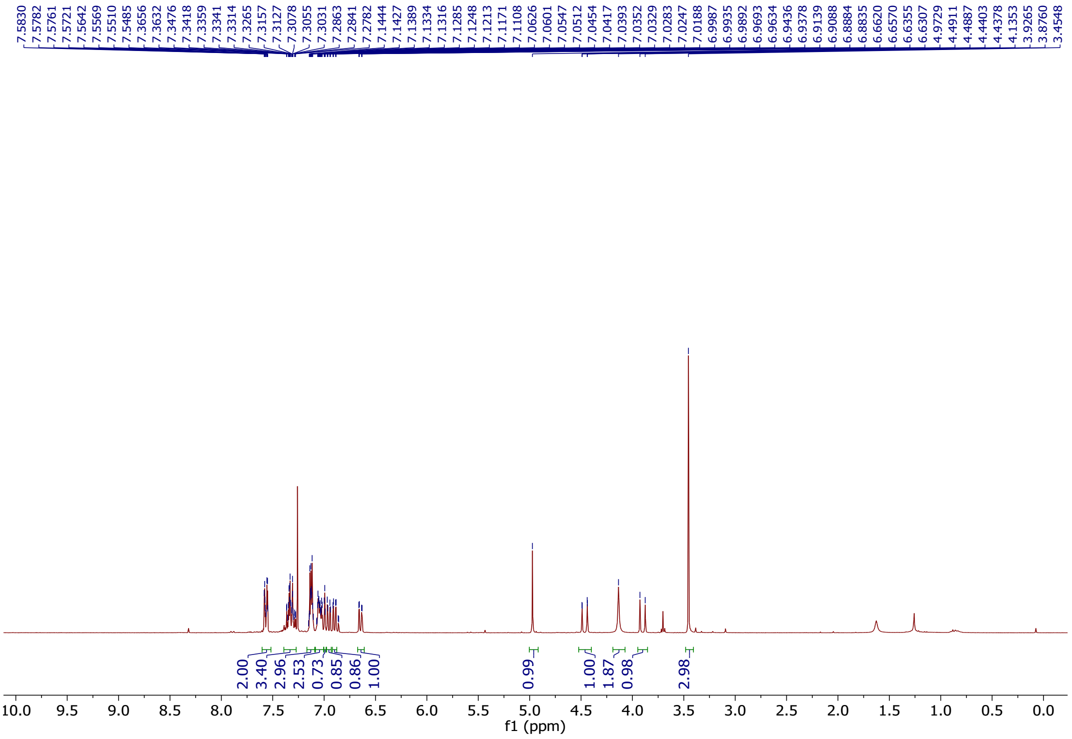

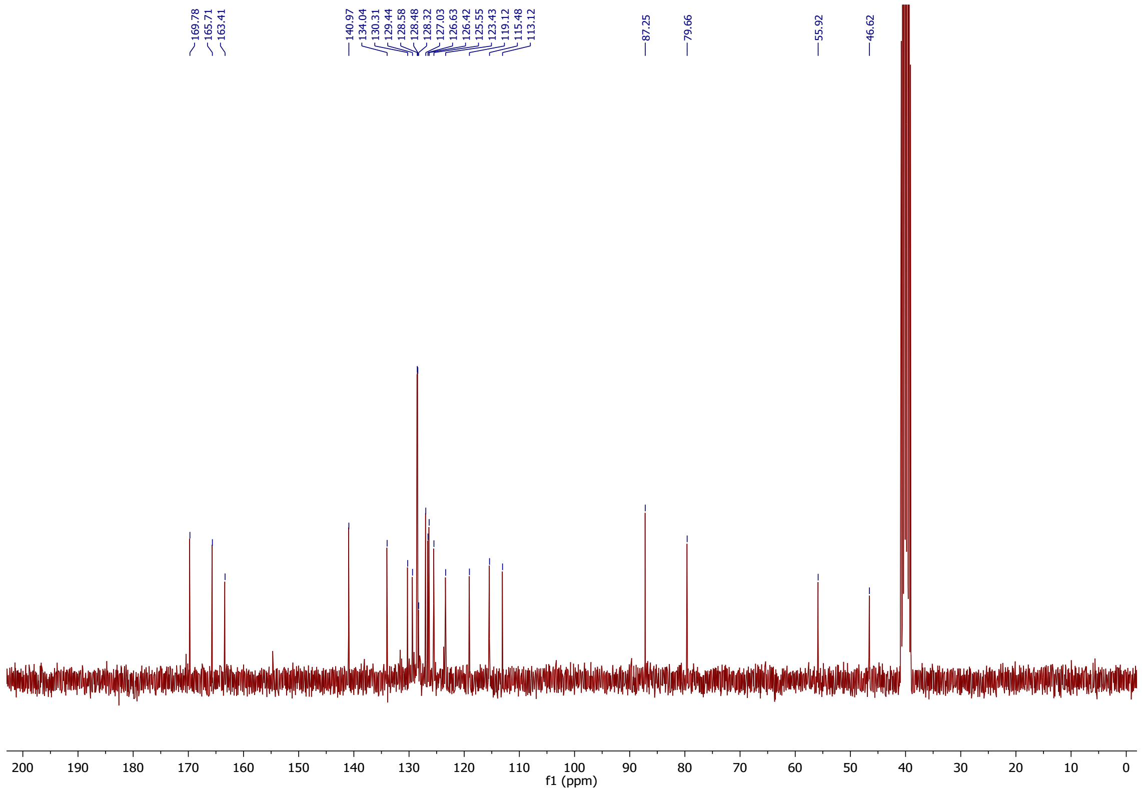

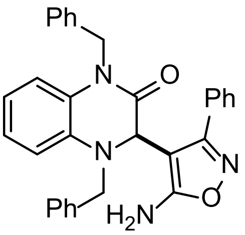

3gb

^{1}H-NMR ($CDCl_3$, 300 MHz)

^{13}C{^{1}H} NMR ($CDCl_3$, 75 MHz)

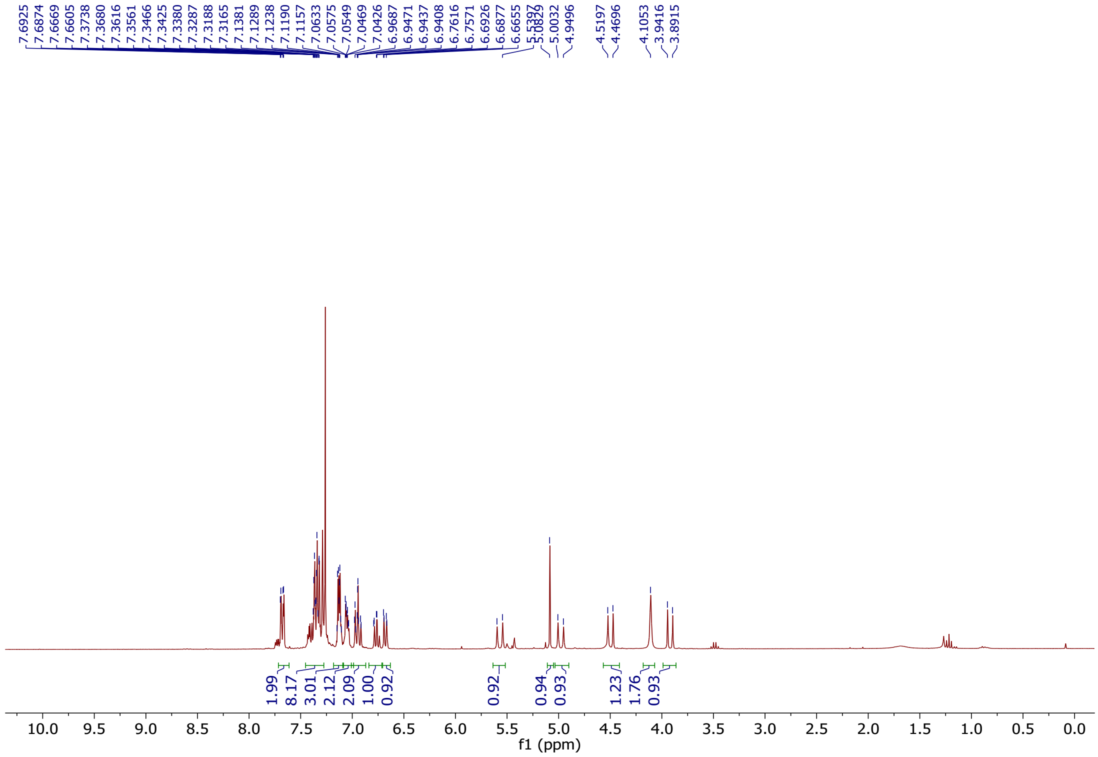

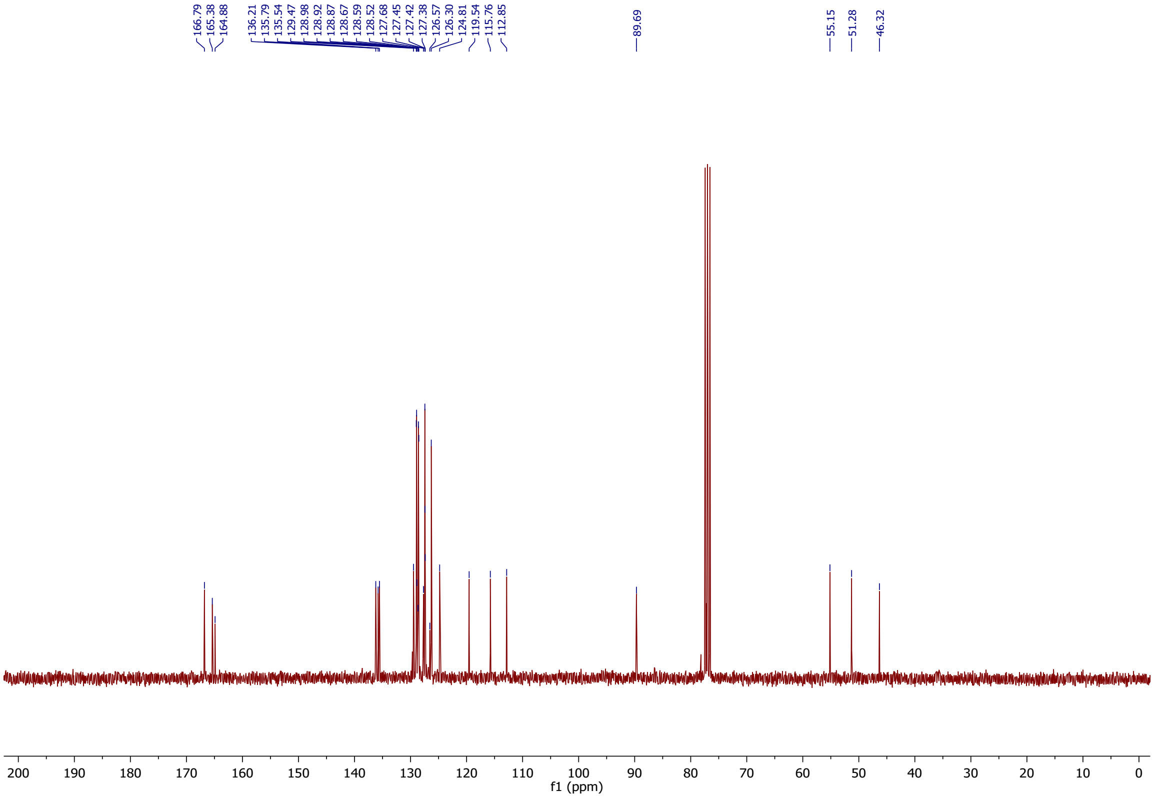

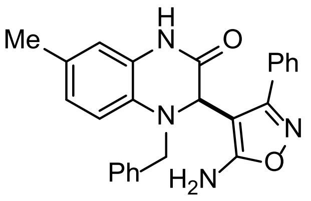

3hb

^{1}H-NMR ($CDCl_3$, 300 MHz)

^{13}C{^{1}H} NMR ($CDCl_3$, 75 MHz)

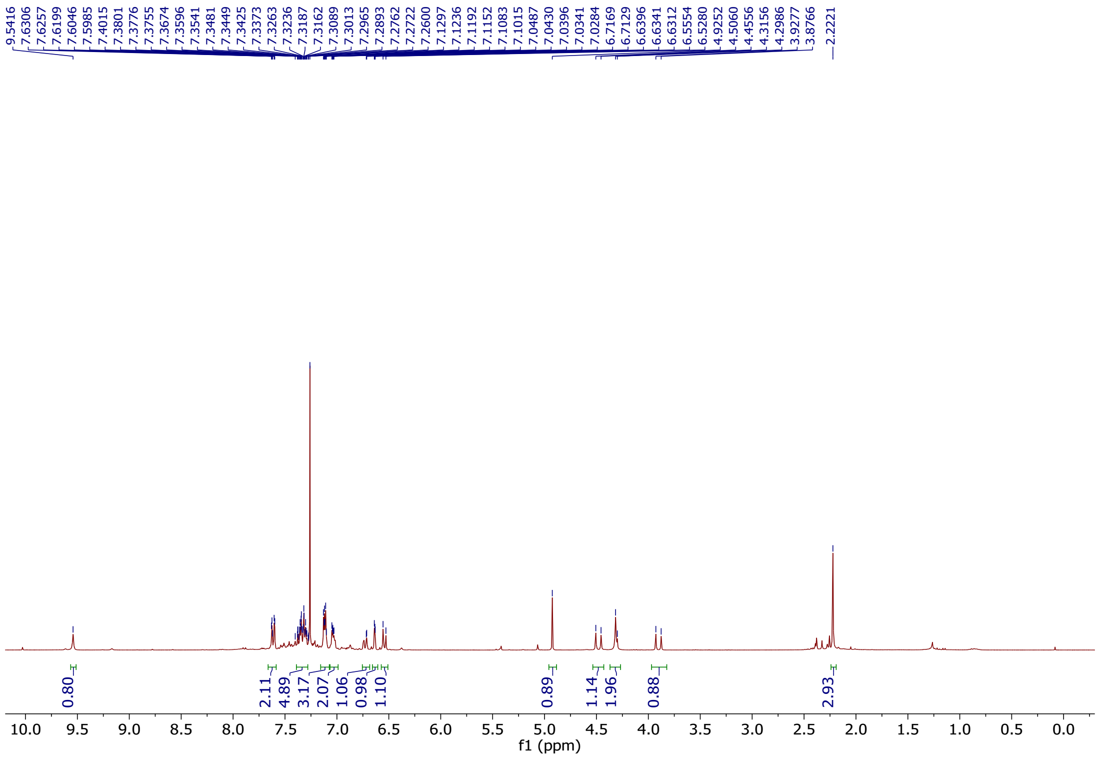

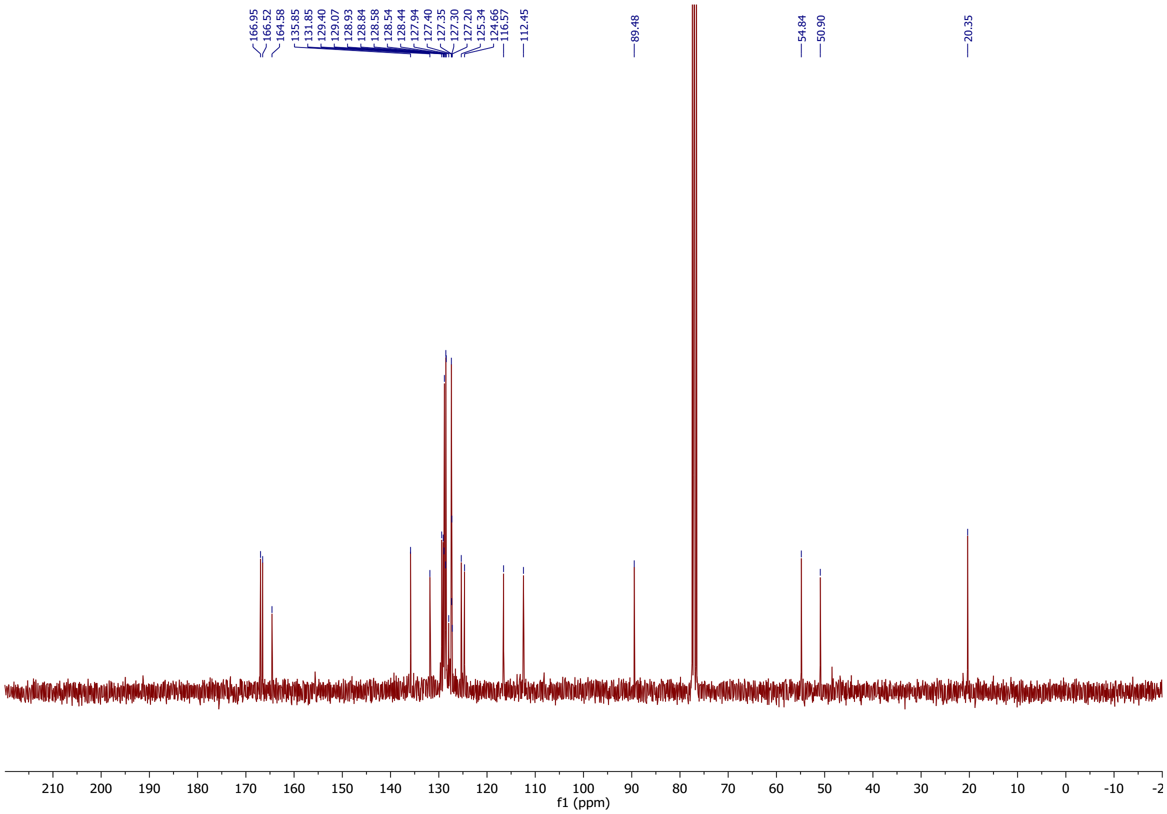

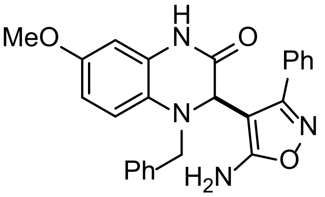

3ib

^{1}H-NMR (DMSO-d_6, 300 MHz)

^{13}C{^{1}H} NMR (DMSO-d_6, 75 MHz)

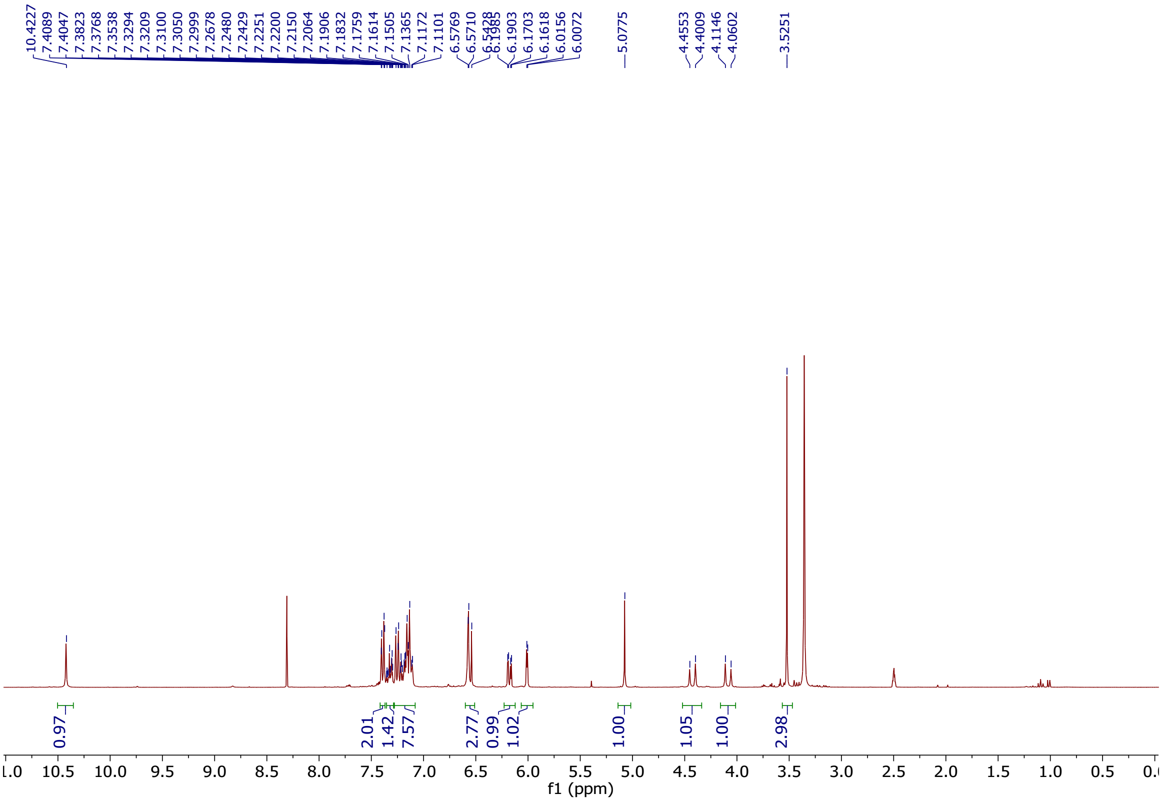

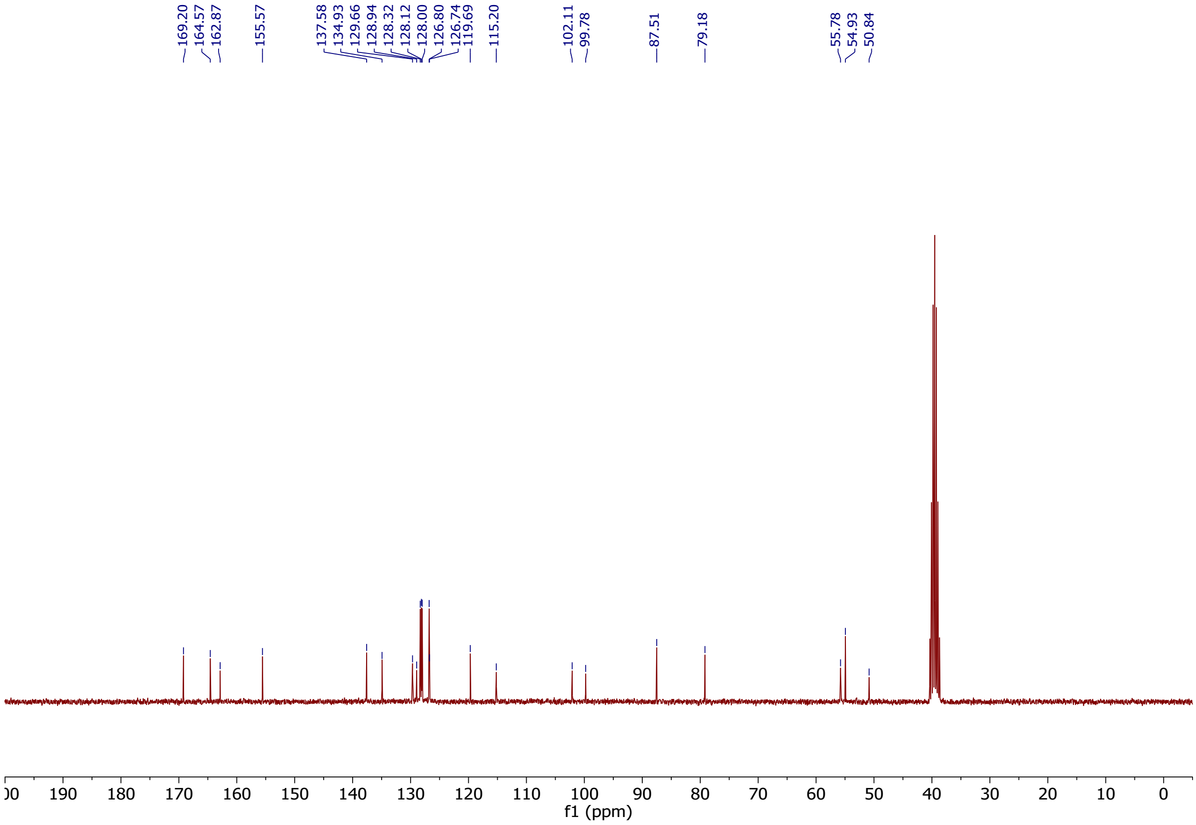

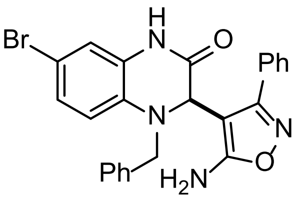

3jb
^{1}H-NMR (DMSO-d_6, 300 MHz)
^{13}C{^{1}H} NMR (DMSO-d_6, 75 MHz)

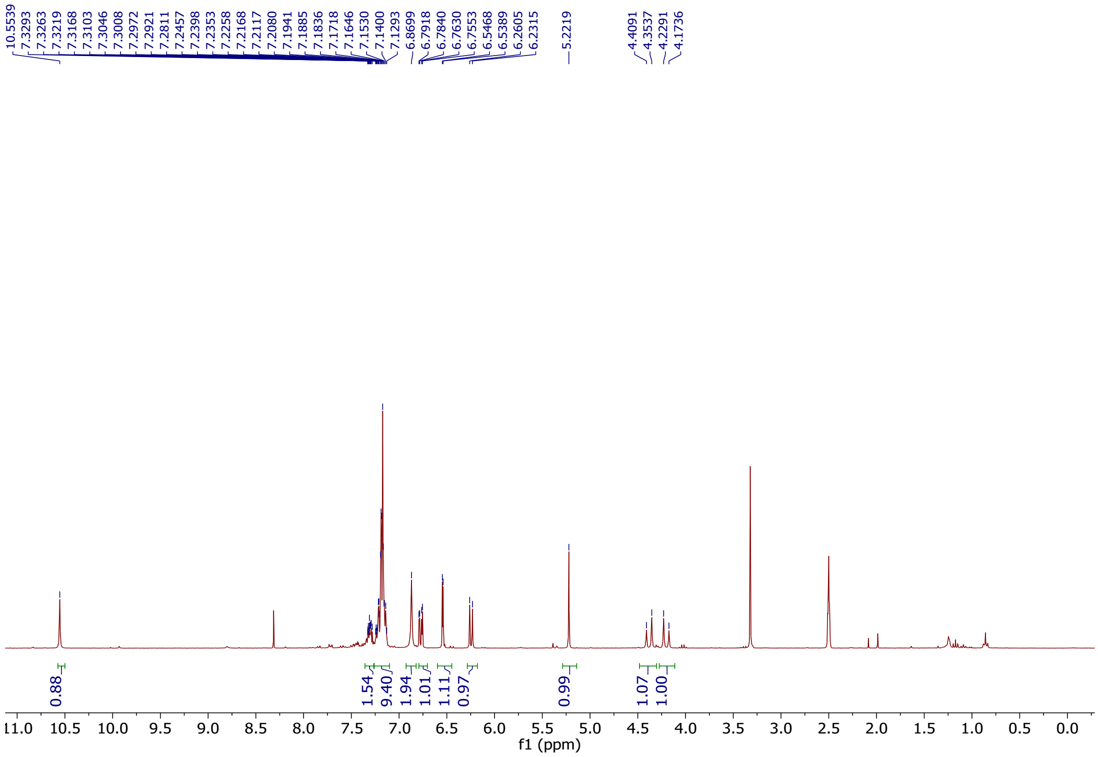

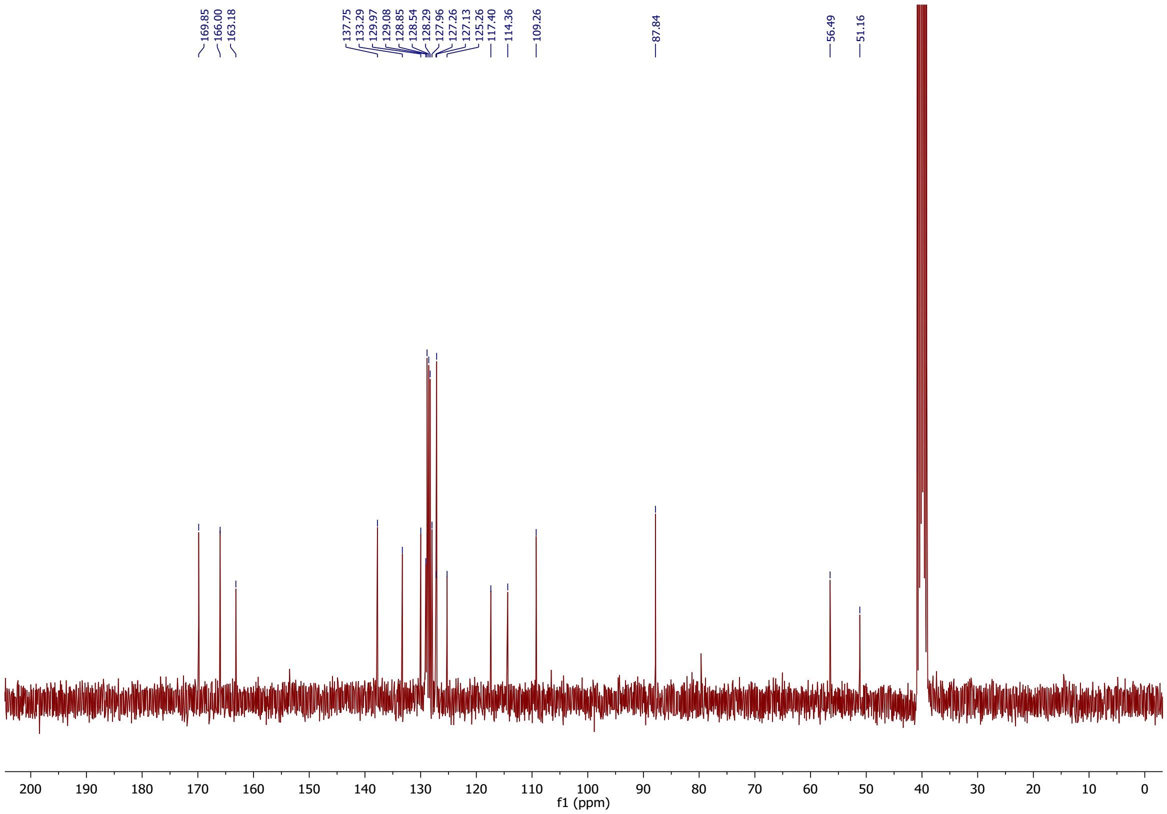

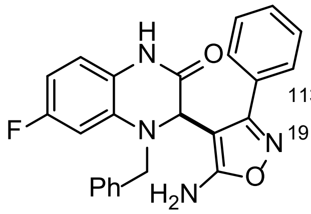

3kb

^{1}H-NMR (DMSO-*d*$_6$, 300 MHz)

[113]C{^{1}H} NMR (DMSO-*d6*, 75 MHz)

^{19}F{^{1}H} NMR (DMSO-*d6*, 282 MHz)

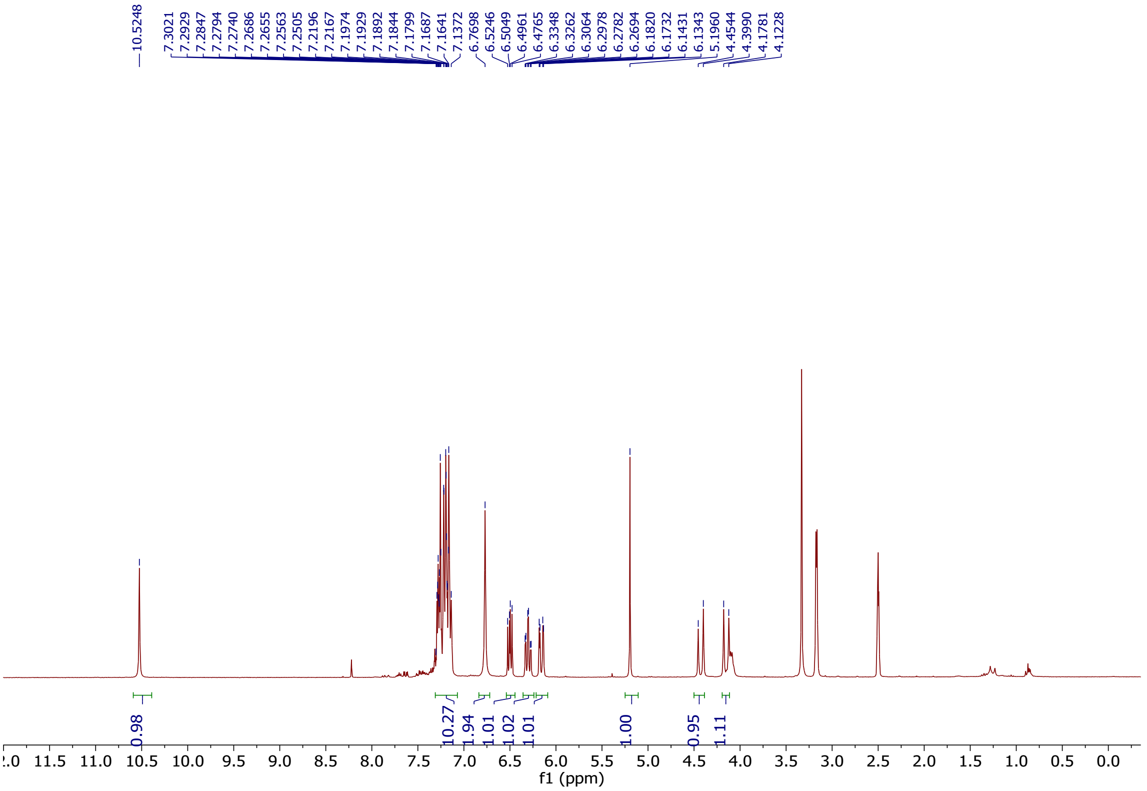

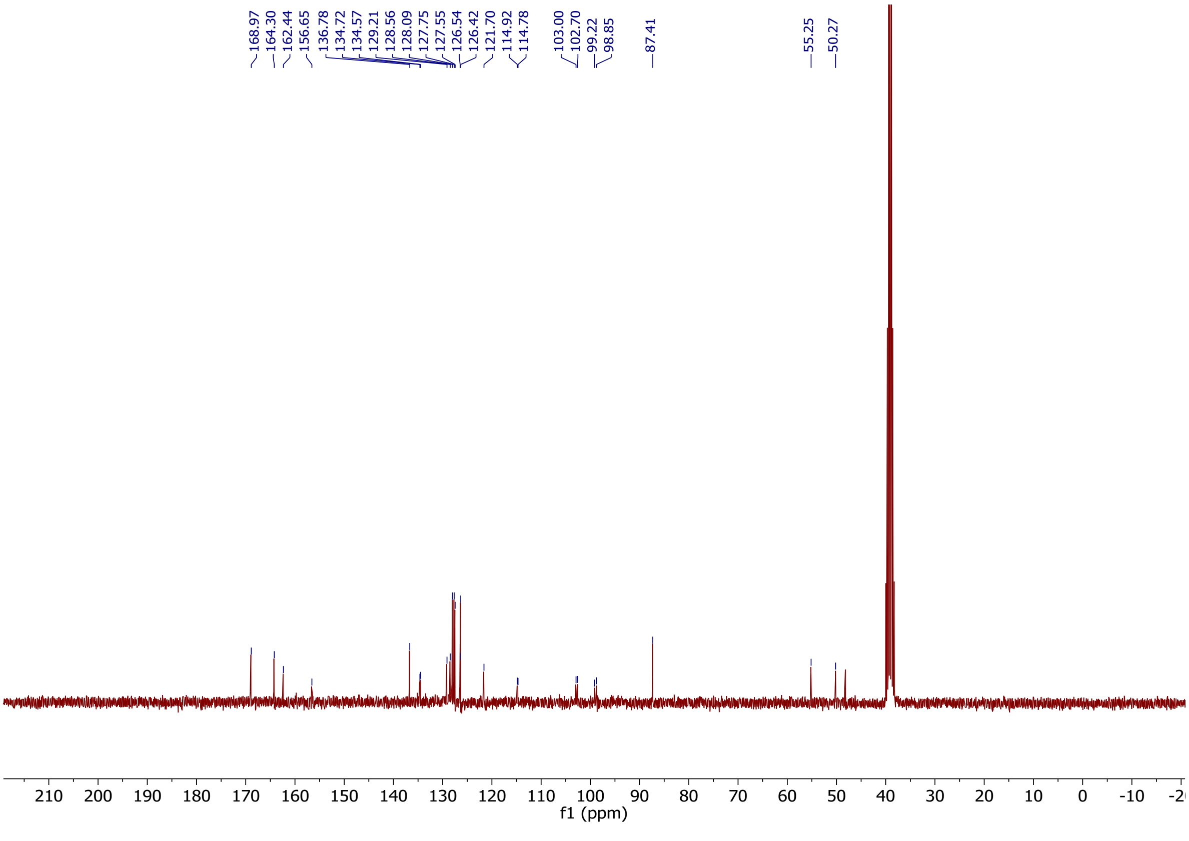

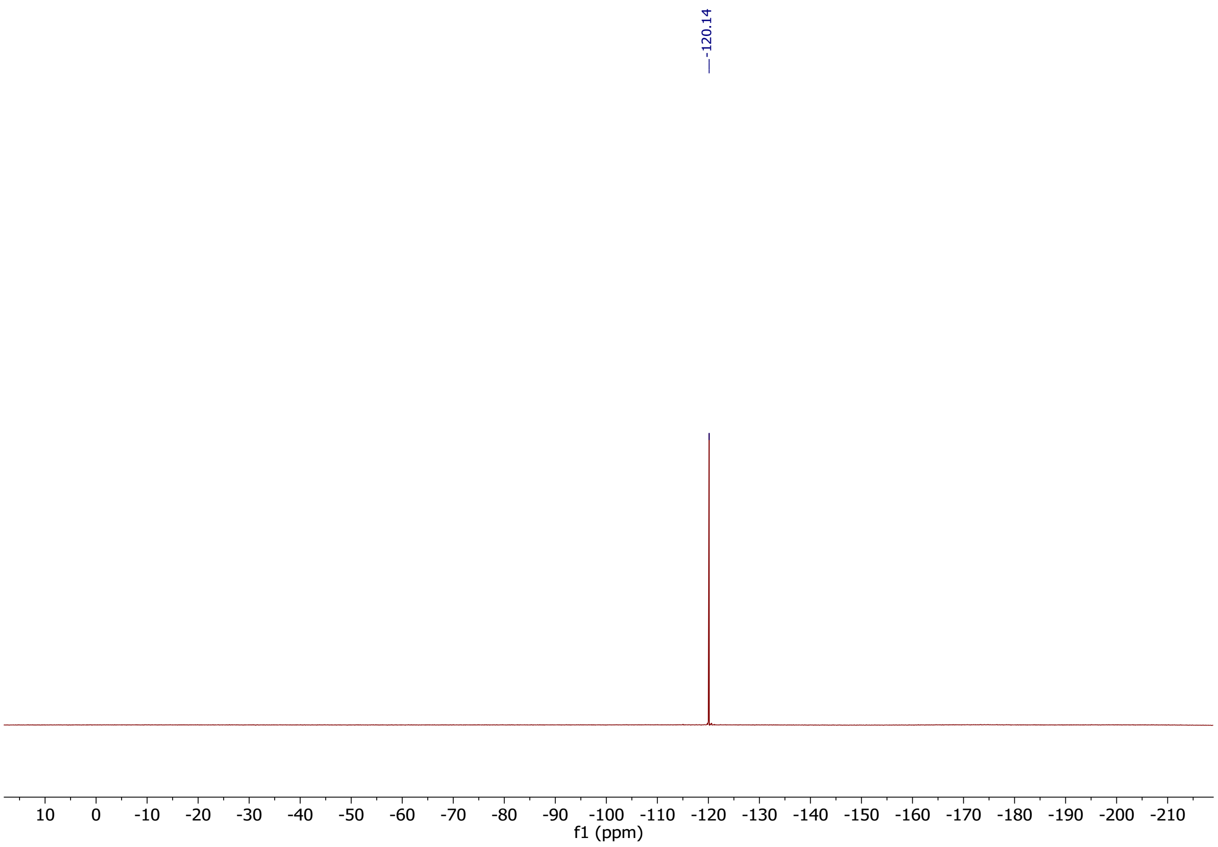

-120.14
10
0
-10
-20
-30
-40
-50
-60
-70
-80
-90
-100
-110
-120
-130
-140
-150
-160
-170
-180
-190
-200
-210
f1 (ppm)

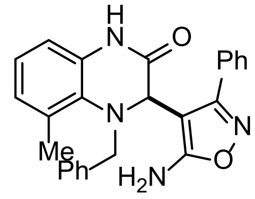

3lb

^{1}H-NMR (DMSO-d_6, 300 MHz)

^{13}C{^{1}H} NMR (DMSO-d_6, 75 MHz)

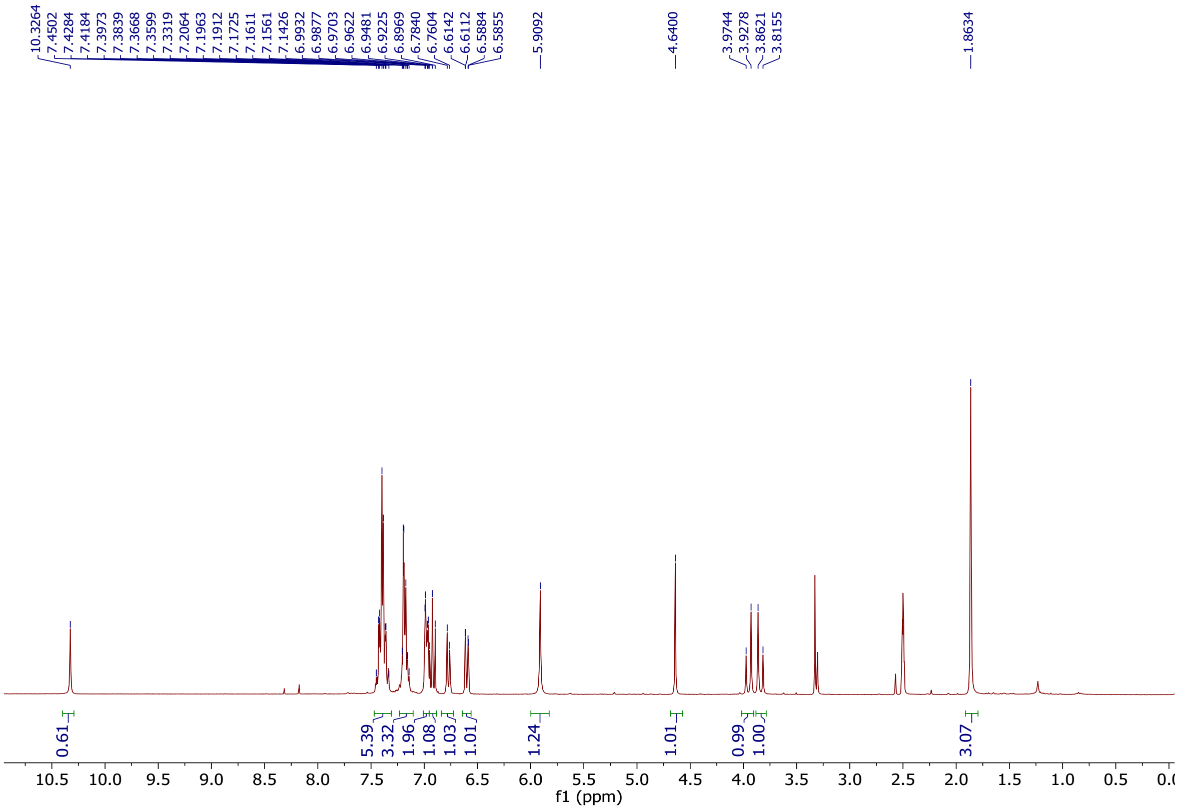

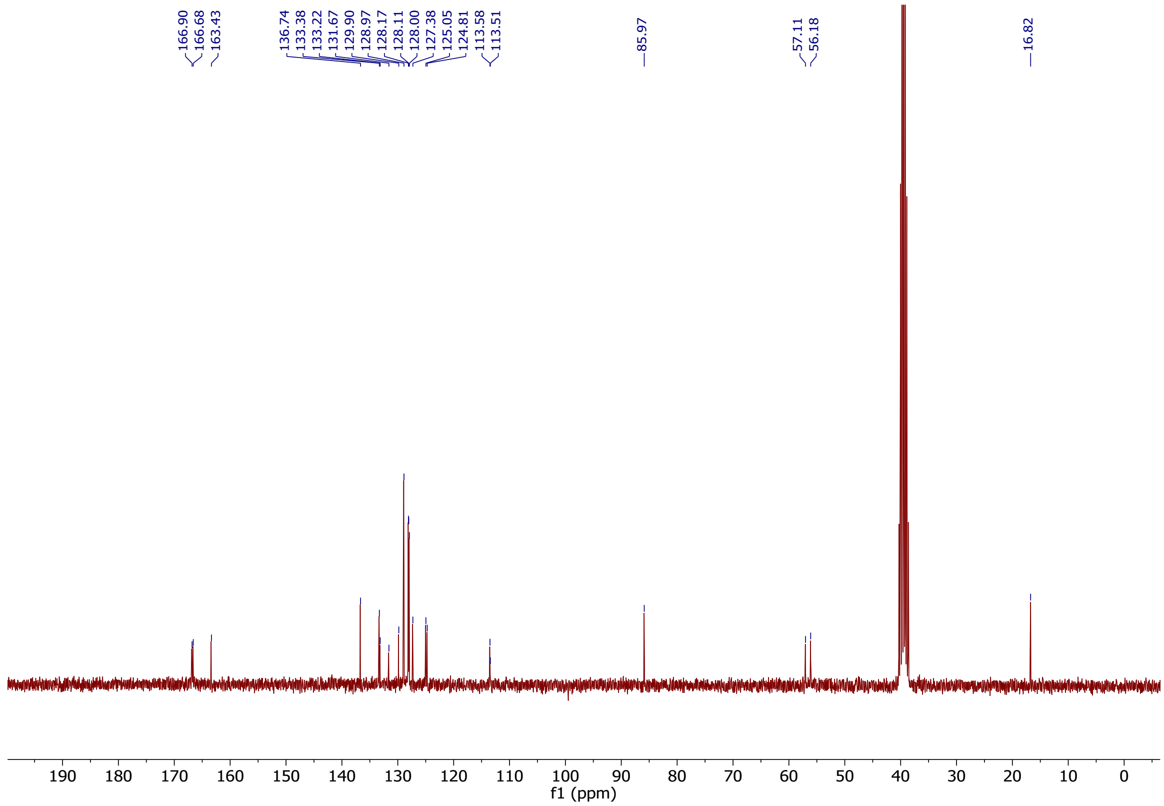

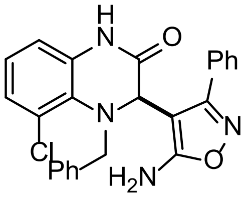

3mb

^{1}H-NMR ($CDCl_3$, 300 MHz)

^{13}C{^{1}H} NMR ($CDCl_3$, 75 MHz)

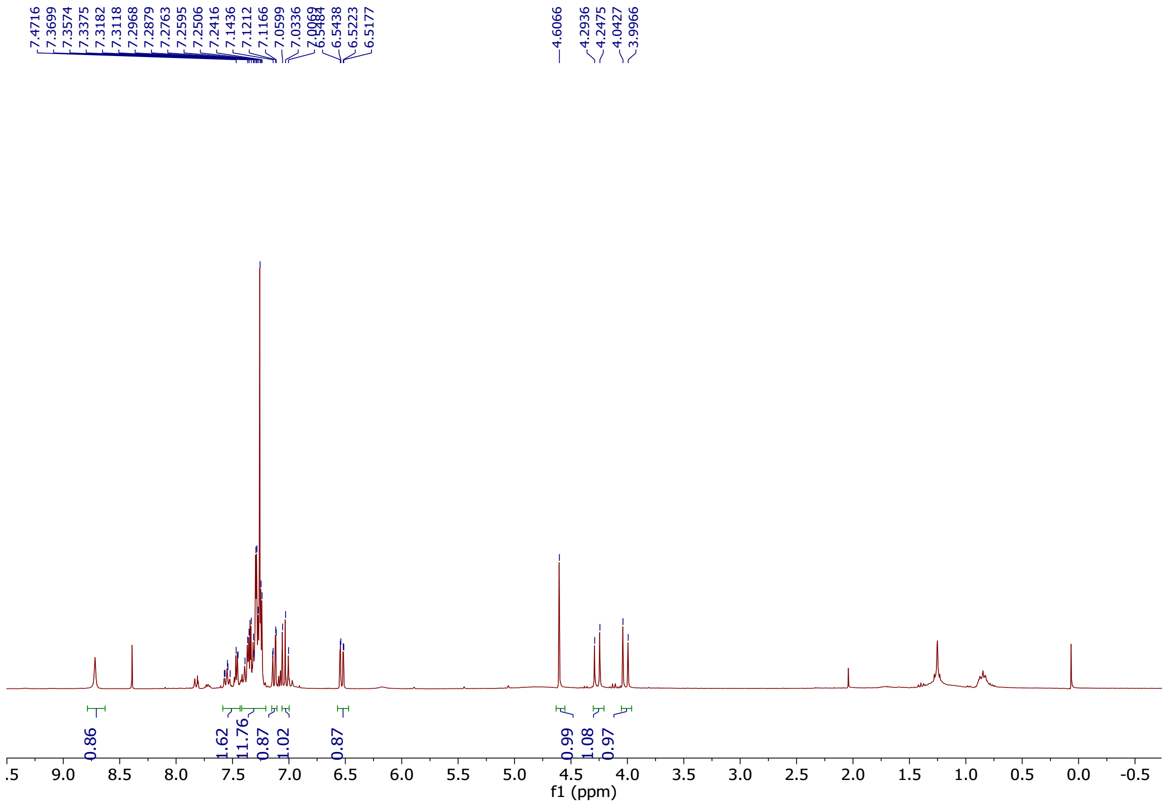

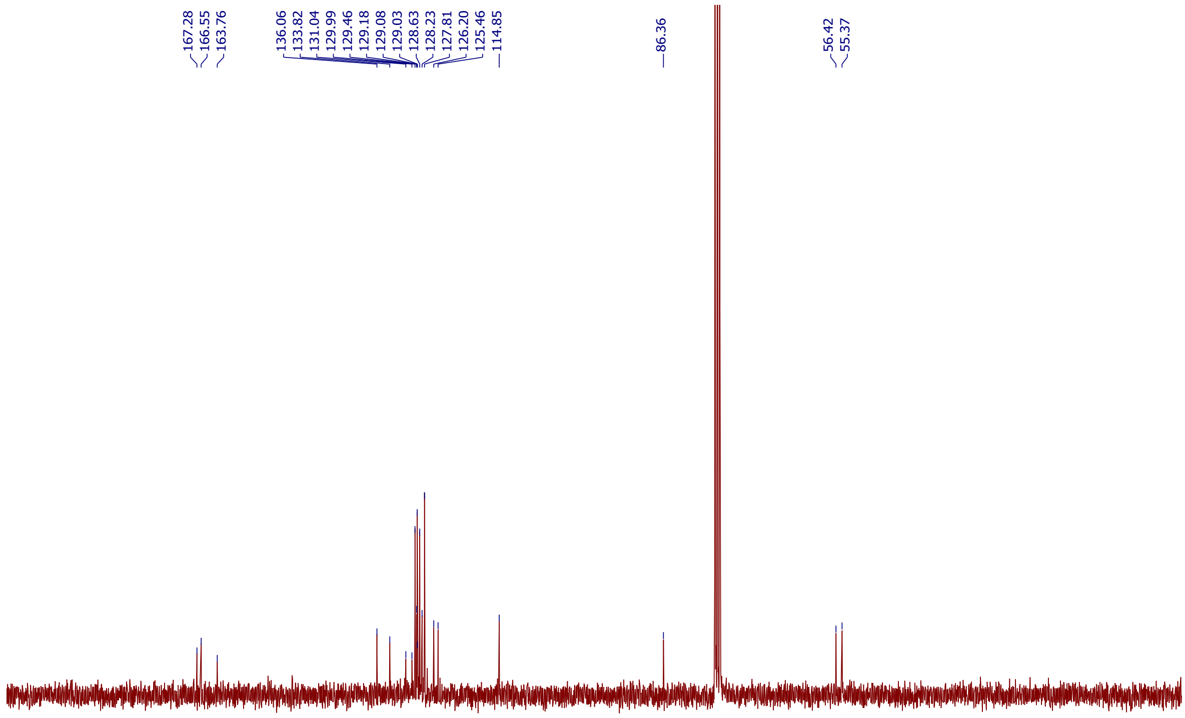

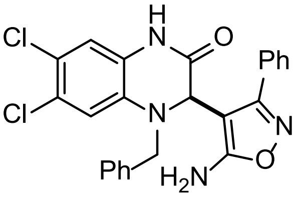

3nb

^{1}H-NMR (DMSO-d_6, 300 MHz)

^{13}C{^{1}H} NMR (DMSO-d_6, 75 MHz)

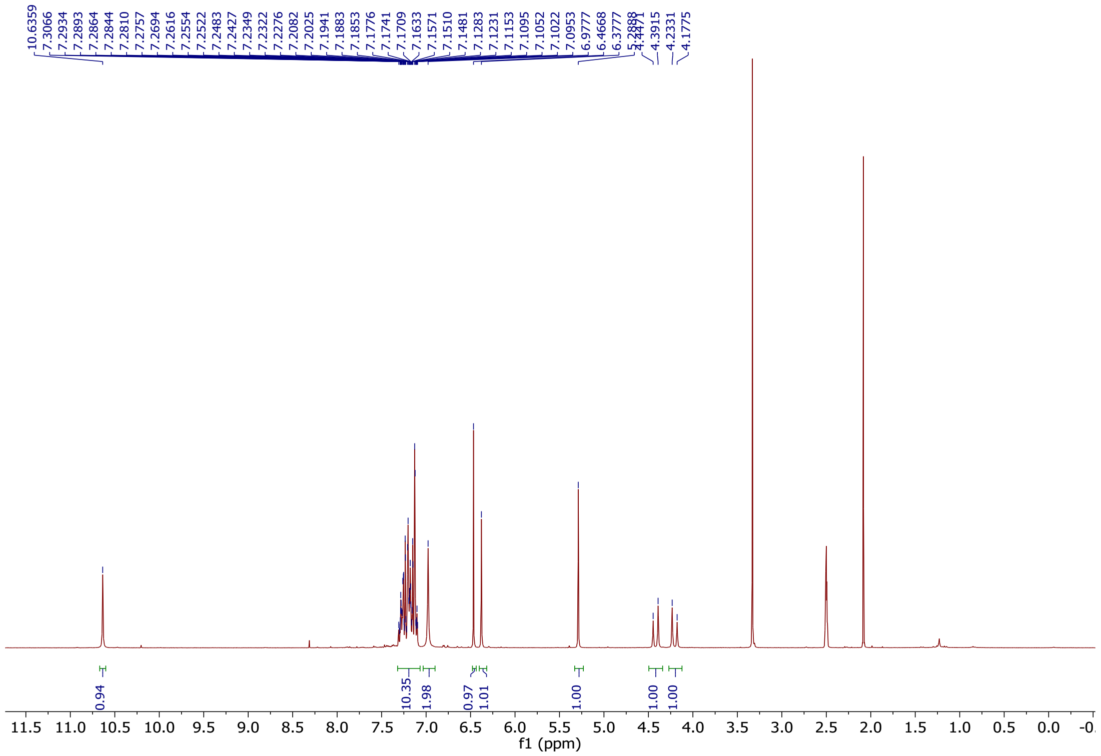

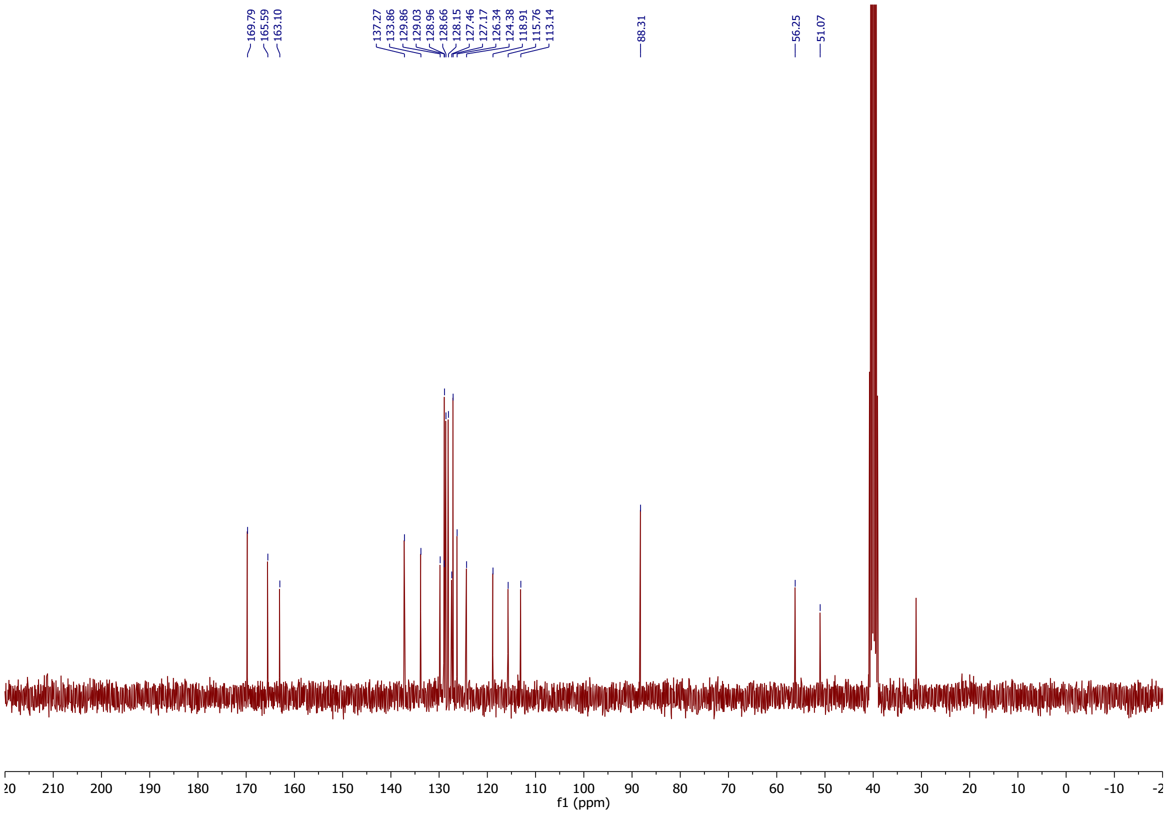

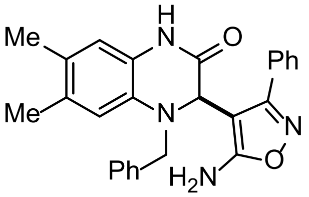

3ob
^{1}H-NMR ($CDCl_3$, 300 MHz)
^{13}C{^{1}H} NMR ($CDCl_3$, 75 MHz)

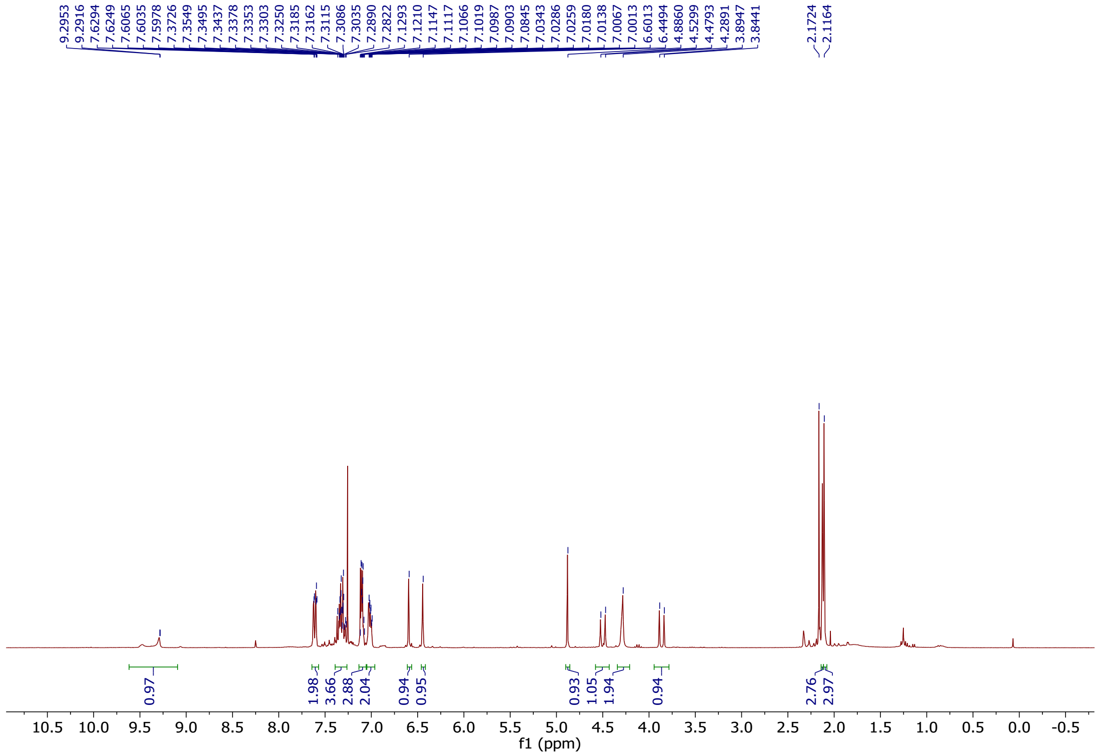

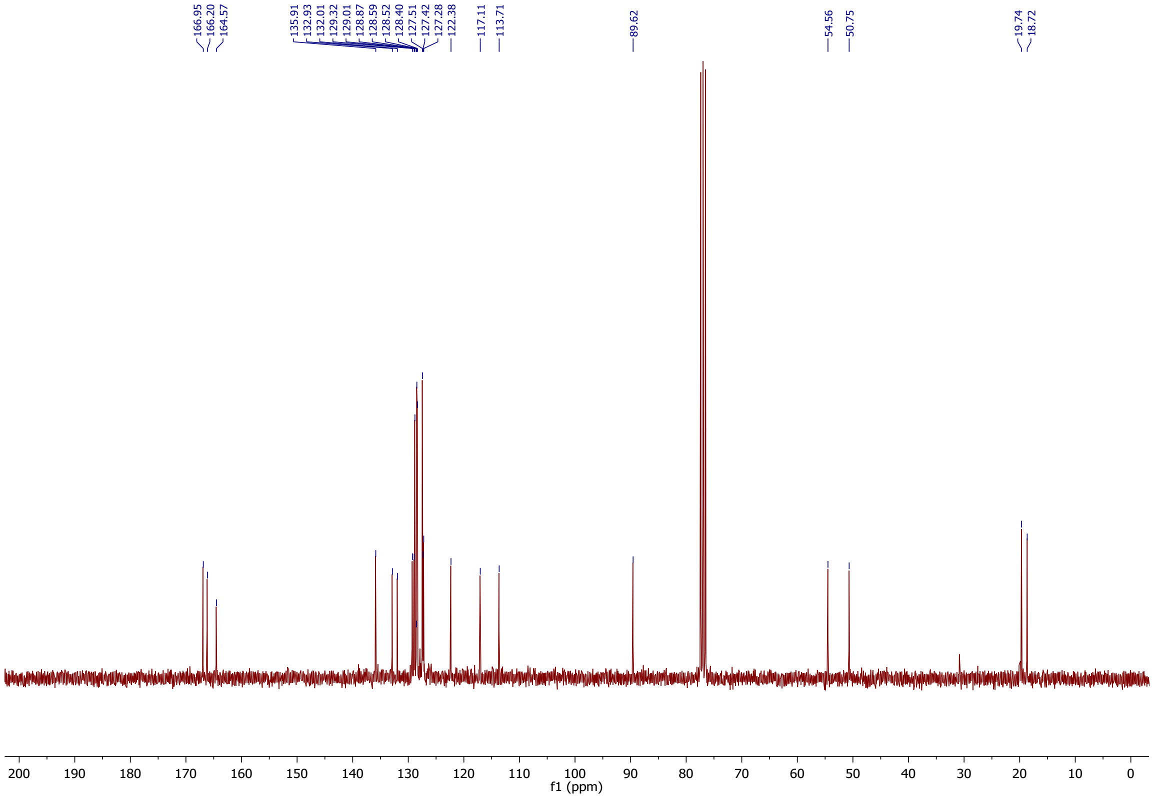

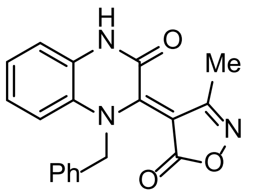

4
^{1}H-NMR ($CDCl_3$, 300 MHz)
$^{13}C\{^{1}H\}$ NMR ($CDCl_3$, 75 MHz)

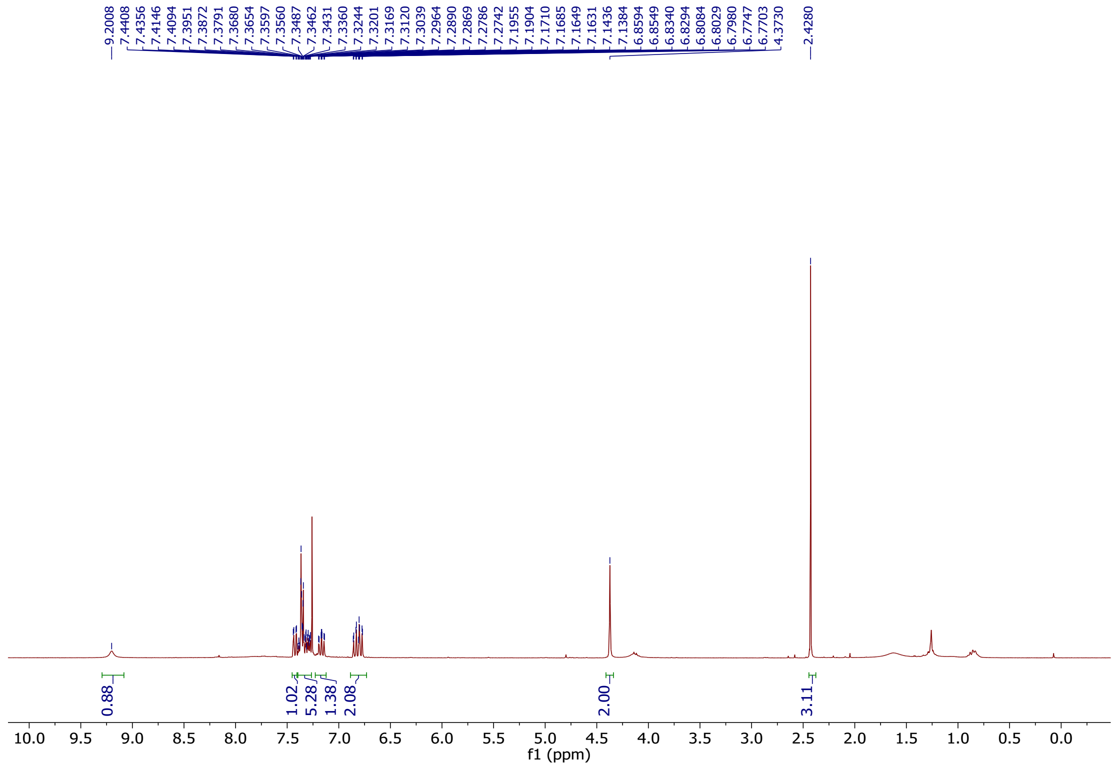

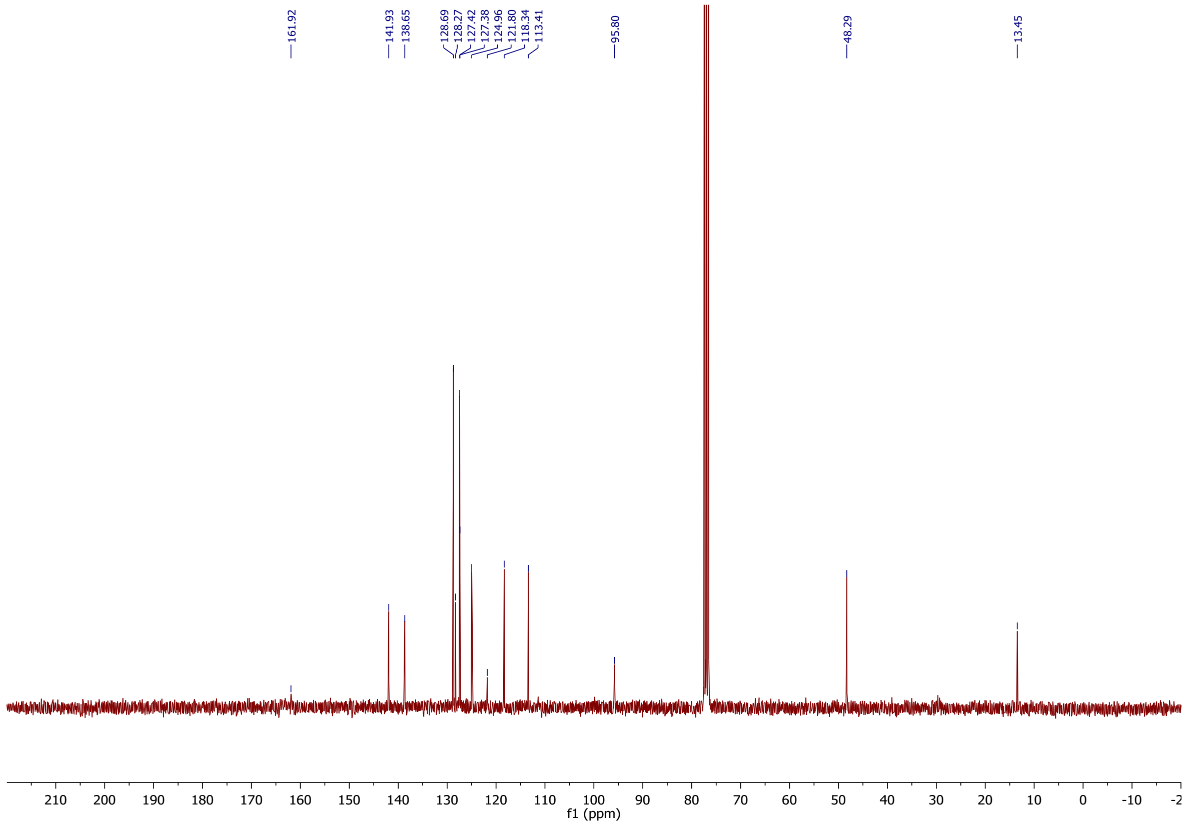

Supplement: Supplementary file 1 [file jo5c02592_si_001.pdf]
